# Comparative analysis of circular RNA enrichment methods

**DOI:** 10.1080/15476286.2021.2012632

**Published:** 2021-12-12

**Authors:** Huajuan Shi, Ying Zhou, Erteng Jia, Zhiyu Liu, Min Pan, Yunfei Bai, Xiangwei Zhao, Qinyu Ge

**Affiliations:** a State Key Laboratory of Bioelectronics, School of Biological Science and Medical Engineering, Southeast University, Nanjing, China; b School of Medicine, Southeast University, Nanjing, China

**Keywords:** CircRNA sequencing, enrichment methods, benchmark, sensitivity, precision

## Abstract

The circRNAs sequencing results vary due to the different enrichment methods and their performance is needed to systematic comparison. This study investigated the effects of different circRNA enrichment methods on sequencing results, including abundance and species of circRNAs, as well as the sensitivity and precision. This experiment was carried out by following four common circRNA enrichment methods: including ribosomal RNA depletion (rRNA**^–^**), polyadenylation and poly (A^+^) RNA depletion followed by RNase R treatment (polyA+RNase R), rRNA**^–^**+polyA+RNase R and polyA+RNase R+ rRNA**^–^**. The results showed that polyA+RNase R+ rRNA **^–^** enrichment method obtained more circRNA number, higher sensitivity and abundance among them; polyA+RNase R method obtained higher precision. The linear RNAs can be thoroughly removed in all enrichment methods except rRNA depletion method. Overall, our results helps researchers to quickly selection a circRNA enrichment of suitable for own study among many enrichment methods, and it provides a benchmark framework for future improvements circRNA enrichment methods.

**Figure:**
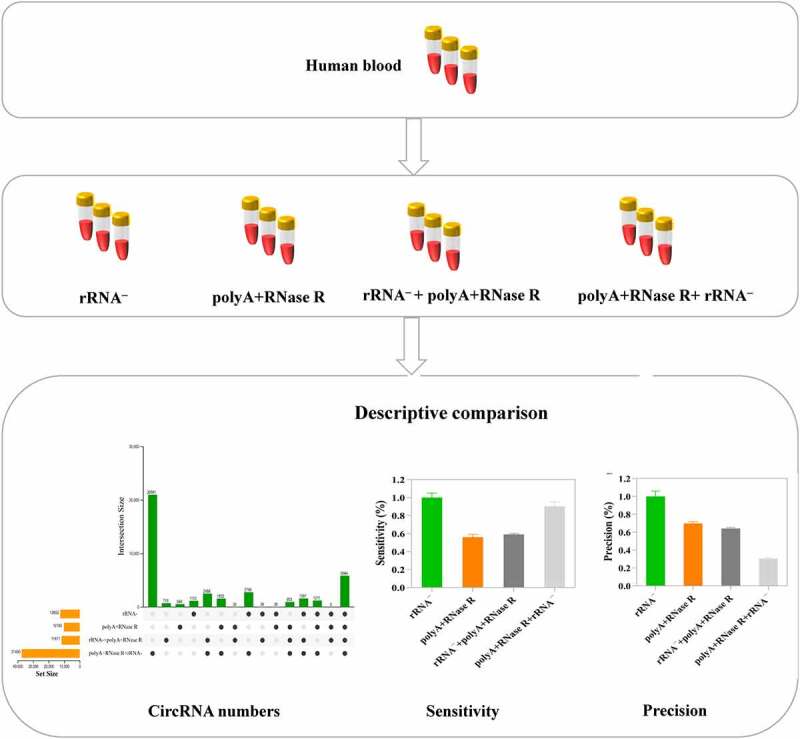

## Background

Circular RNA (circRNA) is a large class of non-coding RNA (ncRNA) with a length of about 100 (nt) ~ 4 kb [1]. Their 5ʹ – or 3ʹ-ends are jointed together, forming covalently closed loop structures. Unlike linear RNA, circRNA, without cap and poly (A) tails, is stable and not easily degraded by exoribonuclease R (RNase R) [2,3]. In addition, circRNAs have a relative low abundance compared with canonical linear mRNA transcripts [4]. Furthermore, previous studies have been shown that circRNA may possess many important functions, including acting as sponges to sequester microRNA (miRNA) or RNA binding proteins (RBPs) [5–7], regulating transcription and splicing [8,9]. More recently, several studies reported that circRNAs acted as miRNAs sponges and involved in many diseases, such as cardiovascular [10], glioblastoma [11], hepatocellular [12], gastric cancer [13] and tumorigenesis [14]. Thus, circRNAs have great potential to play important roles in the process of cellular metabolism and disease. Further explorations on circRNAs are strongly suggested, which will be of far-reaching significance especially on disease occurrence, development, and accurate diagnosis and treatments.

Currently, the characterization and quantification of circRNAs by using high-throughput RNA sequencing (RNA-seq) data has become an emerging problem in the research of circRNA [15]. Although short reading paired-end RNA-seq technology does not fully reveal the whole length of circRNAs, the backspliced junctions could be identified reliably and the circRNAs could be quantified. However, the detection of circRNAs through RNA-seq technologies requires a protocol that can profile non-polyadenylated (non-poly (A)) transcripts in library preparation [16,17]. There are two types of enrichment methods currently that can be used to identify circular RNA by sequencing [18]. The first one is rRNA depletion-based method, which all the mRNA and non-coding RNAs including circRNAs will be retained because of the sequence-specific hybridization during rRNA depletion. The other one is linear RNAs removed method based on the specific structure of circRNAs. CircRNAs can be enriched when the linear RNAs were treated with RNase R, this make it easier to detect lowly expressed circRNA [17]. However, it requires more input total RNA than rRNA depletion method in library preparation [19]. Furthermore, some linear non-polyadenylated and RNase R-resistant RNAs with short 3ʹ overhangs, such as snRNAs, escape this enrichment step, and can interfere with downstream analyses [20]. Therefore, to solve this problem, researchers proposed a new method called RPAD (RNase R treatment followed by polyadenylation and poly(A)^+^ RNA depletion) were proposed to increase the purity of the circRNA enriched prior to sequencing, which enhancing the chances of detecting novel circRNAs and contributing to study the sequence and function [21,22]. Although novel enrichment methods have improved detection of circRNAs, the relative efficiency and accuracy of these methods to enrich for circRNA has not been assessed.

Furthermore, due to the complexity of each circRNA enrichment method, a substantial of technical variation are usually introduced in the enrichment process. One type of technical variable is the change of the amount of circRNA enrichment (i.e. the expression of identified circRNAs by using different enrichment methods). Another variable factor interested is sensitivity (i.e. the percentage of identified circRNAs by using diverse enrichment methods). The third type is the precision by using different enrichment methods. The combination of sensitivity and precision determines the ability to detect relative differences in circRNA expression levels. In order to make a well-informed choice among the available circRNA enrichment methods, it is very important to investigate these different parameters comparably.

In this present study, we compare four commonly used circRNAs enrichment methods (ribosomal RNA depletion (rRNA**^–^**), polyadenylation and poly (A^+^) RNA depletion followed by RNase R treatment (polyA+RNase R), rRNA**^–^**+polyA+RNase R and polyA+RNase R+ rRNA**^–^**) by using a comprehensive set of metrics. Starting from one sample of total RNA from human blood, we constructed a set of libraries for each method, and sequenced them to deep coverage (the sequencing depth and coverage were 3.3 X and 95%, respectively). Then, we compared their linear RNAs removal effect, the number of identified circRNAs, sensitivity and precision. This result indicated that the linear RNAs can be significantly removed in polyA+RNase R, rRNA**^–^**+polyA+RNase R and polyA+RNase R+ rRNA ^–^ enrichment methods compared to rRNA depletion group. PolyA+RNase R+ rRNA ^–^ enrichment method obtained a more circRNA numbers, higher sensitivity and abundance. PolyA+RNase R enrichment methods have a higher precision. Overall, our results helps researchers to quickly selection a circRNA enrichment of suitable for own study among many enrichment methods, and it provides a benchmark framework for future improvements circRNA enrichment methods.

## Materials and methods

### Sample collection and ethics statement

We collected 5 mL of peripheral blood from healthy subject via venipuncture and then stored in EDTA anticoagulant vacutainers. All procedures were reviewed and approved by the Committee on the Ethics of Southeast University before the study began, and performed in strict accordance with the Declaration of Helsinki of the World Medical Association. Written informed consent was obtained from all subjects in this study.

### RNA extraction and degradation assessment

Total RNA of whole blood samples was extracted within 4 hours using Trizol® reagent (Thermo Fisher Scientific, #1559608) following the manufacturer’s instructions, and was treated with RQ1 RNase-free DNase (Takara Co. Ltd., Japan) to eliminate genomic DNA contamination. Briefly, 200 μL of each blood sample was incubated with 1 mL Trizol® reagent for 20 min at room temperature, followed by supplementation with 200 μL of chloroform. After vigorous mixing and centrifugation at 12,000 *g* for 20 min at 4°C, the supernatant was transferred to a new 1.5 mL tube. Add 600 μL of isopropanol to the supernatant in the 1.5 mL tube and invert or vortex to mix. Incubate at −20°C for 1 h, followed by centrifugation at 12,000 *g* for 20 min at 4°C to pellet RNA. The pellet was washed with 1 mL 75% ethanol and air-dried for 30 min. RNA was dissolved in 10 μL RNase-free water. Then, the quality and quantity of the total RNA were measured by Agilent 2100 Bioanalyzer (Agilent Technologies, Palo Alto, CA, USA). The OD260/OD280 ratio was used as the RNA purity index. The RNA purity is qualified if the OD260/OD280 ratio ranges from 1.8 to 2.1. Its integrity was further measured by electrophoresis in 1.5% formaldehyde denaturing agarose gels.

### Targets and PCR primers

In order to study the influence of different treatment methods on the effectiveness of linear RNA depletion, we selected two circRNAs from the circular RNA database circBase (http://www.circbase.org/) [23] and evaluated by quantitative real-time PCR. All the divergent primers for circRNA detection were designed using the CircInteractome web tool (http://circinteractome.nia.nih.gov/Divergent_Primers/divergent_primers.html) [24]. Convergent primers for detection of linear RNAs were designed using the NCBI primer tool. The primer sequences are presented in Table 1.

**Table 1.** The list of primers used in this research

CircRNA ID	Forward Primer (5`>3`)	Reverse Primer (5`>3`)	PCR Product Size (bp)
18S	CAGGTCTGTGATGCCCTTAGA	GCTTATGACCCGCACTTACTG	89 bp
NRIP1	ACAGCCAGAAGATGCACACT	CAAGCTCTGAGCCTCTGCTT	124 bp
GAPDH	CCCTTCATTGACCTCAACTACATG	TGGGATTTCCATTGATGACAAGC	112 bp
hsa_circ_0001445	CAAGATGGGCGAAAGTTCA	GCACCTCTTTCCAAAATACCA	101 bp
hsa_circ_0004771	TCCGGATGACATCAGAGCTA	GGCTGTGTTTCTCCCAAATG	159 bp
hsa_circ_0136151	CAATCACACGGGTGCTCCA	GTCGGCGGTACAGCTTAGAG	159 bp
hsa_circ_0133524	CCAAAGTAAAGCATTGAGTTACAGC	AGGTGGGAGTAGACACCACT	71 bp
hsa_circ_0029703	ACCGCTTGTTGGACAGTGAA	CAGTTCATTCTGATTTGACGATGC	75 bp
hsa_circ_0109315	AAGTGTAATTACTGTCAAACGACTG	TTTGCTCTGGGCAGTTGTGAG	121 bp
hsa_circ_0024169	ACGTTATTTAGAAACAAGACGAGAATGT	TCATCCCAAAGACAGACTGCAT	96 bp

### Depletion of rRNA or linear RNA

Total RNA was purified using 1.8 × Magnetic Beads (VAHTATM RNA Clean Beads, N412) after it was treated with RNase-free DNaes I (Qiagen, Hilden, Germany). Then, the total RNA of met the following requirements were submitted to transcriptome sequence libraries preparation and downstream experiments: the RIN (RNA integrity number) > 7 and the 28S: 18S rRNA Ratio > 1.5. Subsequently, the purification of RNA were equally divided into four groups, namely the rRNA ^–^ depletion, the polyA+RNase R, the rRNA^–^+polyA+RNase R and the polyA+RNase R+ rRNA^–^. There are three replicates per treatment group. A total amount of 2 μg total RNAs was used as input material for each group to enrich the circRNAs. Firstly, ribosomal RNA (rRNA) was removed by Illumina Ribo-zero rRNA Removal Kit (Illumina, USA), and rRNA-free residue was cleaned up by ethanol precipitation (rRNA ^–^ depletion group). Secondly, rRNA^–^+polyA+RNase R group was prepared as follows: the rRNA were removed by Illumina Ribo-zero rRNA Removal Kit, then remove rRNA samples were subjected to poly (A) tailing in 20 μL reaction using the poly (A) Tailing Kit (Thermo Fisher Scientific, #AM1350) following the manufacturer’s instructions. In short, the 20 μL reaction contained 4 μL 5 × E-PAP buffer, 4 μL 25 mM MnCl_2_, 4 μL 10 mM ATP solution, 0.5 μL RiboLock RNase inhibitor (Thermo Fisher Scientific, #N8080119), 2 U E-PAP (2 U/μL), and incubated for 30 min at 37°C. After incubation, RNA was cleaned using 1.8× Magnetic RNA Beads and dissolved in nuclease-free water. The RNA were then incubated at 37°C for 30 min in a 20-μl reaction, including 2 μL 10× Reaction Buffer, 0.5 μL RiboLock RNase inhibitor, 2 U RNase R (2 U/μL) (Epicentre, #RNR07250). Thirdly, the polyA+RNase R treatment group was performed similarly except without rRNA depletion. And lastly, the polyA+RNase R+ rRNA ^–^ group were treated as the above ones just removing the rRNA by using the Illumina Ribo-zero rRNA Removal Kit. In addition, it is worth noting that in the process of experimental design, the samples of each treatment group were equally divided into two parts, one without any pretreatment was used as the negative control, and the other with linear RNA removal was used as the experimental group, which were directly compared quantitatively with conventional internal reference. Then, all the treated RNAs of pretreatment were submitted to library preparation and quantitative PCR (qPCR).

### cDNA synthesis and quantitative PCR (qPCR)

Processed RNAs were reversed transcription to synthesize the first-strand cDNA using a RT-PCR kit (Takara Co. Ltd., Japan) according to the manufacturer`s instructions. The reaction volume was 10 µL, including 2 µL buffer (5×), 0.5 µL dNTP mixture (10 mM each), 0.25 µL RNase inhibitor (40 U/µL), 0.5 µL dT-AP primer (50 mM), 0.25 µL ExScript^TM^ RTase (200 U/µL) and 6.5 µL DEPC water. Thermal cycling conditions were 42°C for 40 min, 90°C for 2 min, and 4°C forever.

After reverse transcription, quantitative PCR (qPCR) was employed with the SYBR Green II Fluorescence Kit (Takara Bio. Inc., Japan), containing 2 µL cDNA template (equivalent to 100 ng cDNA), 0.4 µL of each primer (10 µmol/L), 10 µL 2 × SYBR premix ExTaq^TM^ mix (TaKaRa), 6.8 µL dH_2_O and 0.4 µL ROX Reference DyeII (TaKaRa). The reaction programme was set as follows: initial denaturation at 95°C at 30 s followed by 40 cycles, denaturing at 95°C for 10 s, annealing and extension at 60°C for 40 s. The glyceraldehyde-3-phosphate dehydrogenase (GAPDH) gene was used as an internal control. Three repeats were analysed from each treatment group. The percentage (%) of linear RNA left were determined following the method described by Panda AC, et al. [25] after diverse treatment methods. The relative expression levels of each gene were calculated using the 2^−∆∆Ct^ method. The ∆Ct value was measured by subtracting the Ct value of GAPDH mRNA from the Ct value of the targets. Melting curve (single peak), reproducible correlation (0.998 > R^2^ > 0.983) and the amplification eciencies (from 0.89 to 1.14) were used to evaluate the validation of the reaction.

### Library preparation and sequencing

The cDNA libraries were prepared by NEBNext® Ultra™ II Directional RNA Library Prep Kit for Illumina® (NEB, USA, #E7760S) following manufacturer’s recommendations. Briefly, the first strand cDNA was synthesized using random primers and M‐MuLV Reverse Transcriptase (RNaseH^‐^) after fragmentation of the RNAs. Then, the second strand cDNA was synthesized with DNA polymerase I and RNase H, and also, dUTP was introduced in this step. Subsequently, remaining overhangs were converted into blunt ends via exonuclease/polymerase activities. After adenylation of 3ʹ ends of DNA fragments, NEBNext Adaptor with hairpin loop structure were ligated to prepare for hybridization. 150–200 bp of cDNA fragments were enriched in the following size selection step by using AMPure XP beads system (Beckman Coulter, Beverly, USA). Then, the libraries were digested with 3 μL USER Enzyme (NEB, USA) at 37°C for 15 minutes. Then, preamplification was performed with Phusion High‐Fidelity DNA polymerase, and Index was introduced in this step. Finally, the PCR products were purified and cDNA library concentration was assessed using a Qubit® 2.0 fluorometer. The library was sequenced by Illumina HiSeq X-10 (Illumina Inc., San Diego, CA, USA) sequencer using a 2 × 150 bp paired-end pattern (PE150).

### Data filtering and quality control

Prior to alignment and assembling, clean data were obtained by removing the raw reads with adaptors, unknown nucleotides greater than 5% and low-quality reads (the bases with quality value Q < 20 accounted for more than 50% of total bases). The Q20, Q30, and GC content of the filtered clean data were calculated. Only the high-quality clean data could be used for downstream analysis.

### CircRNA prediction and transcriptome analysis

The clean reads were mapped to human reference genome (UCSC hg19) with BWA firstly [26]. The circRNAs were then identified and quantified by using CIRI2 [27]. The expression level of circRNAs was determined by the number of reads that support the splicing junction sites. The data of circBase was combined with the identification results of circRNAs. The counts of reads that spanned over back-splice junction sites were normalized as the number of per transcripts per million (TPM). The differential circRNAs expression analysis was performed using DESeq2 R package (1.16.1). Log_2_ |fold change| ≥ 1 and *p* < 0.05 were considered as differentially expressed circRNAs (DE-circRNAs).

### The calculation methods of sensitivity and precision

The sensitivity and precision are defined as follows [28,29]: Sensitivity = TP/(TP+FN); Precision = TP/(TP+FP). Where TP is the true positive; FP is the false positive; and FN is the false negative. To evaluate the performance on balancing sensitivity and precision, *F1*-score was also employed, which is calculated by the following formula: *F1*-score = (2× Sensitivity × Precision)/(Sensitivity + Precision). It is worth mentioning that sensitivity assessment refers to the ratio of true positive genes detected at the same sequencing depth. Precision is considered to be the reproducibility of gene expression level estimation. And the combination of sensitivity and precision determines the ability to detect relative differences in circRNA expression levels.

### Statistical analyses

Data for qPCRs were subjected to statistical computing using the SPSS 20.0 software package (SPSS Inc. Michigan Avenue, Chicago, IL, United States). Statistical significance was assessed by Student’s t-test. To calculate a *P*-value for linear RNA removal efficiency and mapped reads, the Fisher’s exact test was used. Data were shown as means ± standard error of the mean (means ± SEM). Figures were produced using GraphPad Prism 7.0 (GraphPad Software, Inc. La Jolla, CA, USA) and R package.

## Results

### Generation of circRNA enrichment libraries

**Figure 1.**
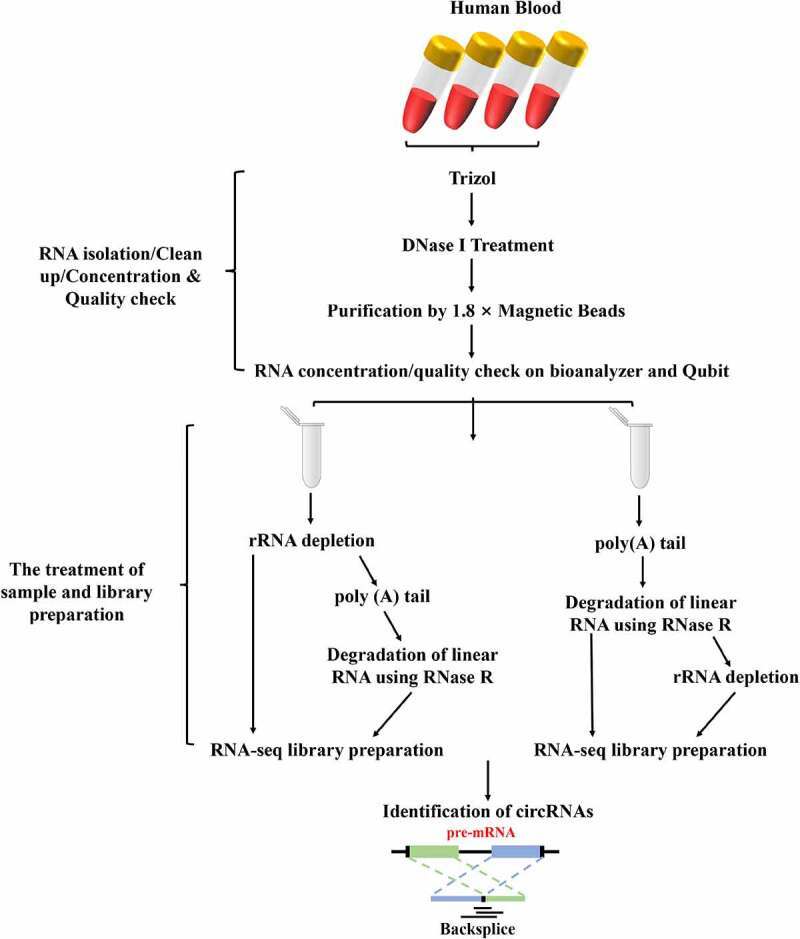
The workflow of total RNA isolation, circRNA library preparation and sequencing. Firstly, the circRNAs were enriched by using different enrichment methods. Subsequently, the libraries were sequenced on an Illumina Hiseq 4000 platform and circRNA identified.

In RNA sequencing, the variation of results is usually caused by biological and technical variation. In this present study, we mainly compared the technical variation because of the peripheral blood was collected from the same healthy subject which the biological variation was eliminated. There are three independent replicates in the comparison of the four tested circRNA enrichment methods (Figure 1). The sequencing data of detailed and quality control results are shown in Table S1.

### The removal efficiency of diverse circRNA enrichment methods on linear RNAs

**Figure 2.**
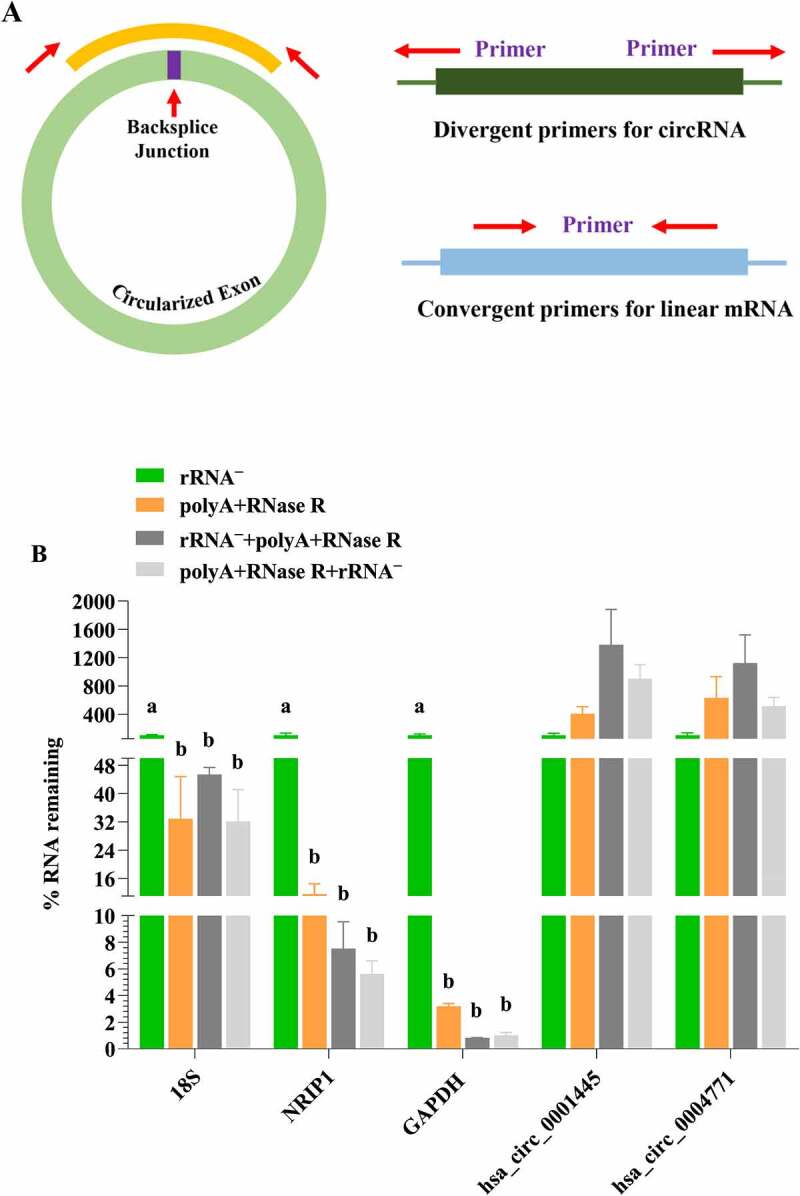
The validation of linear RNA removal efficiency using four diverse circRNA enrichment methods. (a) A schematic illustration for the design of the primers used to enrich for circRNA and their corresponding mRNA. circRNA: primers facing outwards; linear RNAs: primers facing inwards. (b) Subsets of blood circRNAs and mRNAs were quantified by qPCR by diverse enrichment methods. each data represents the mean of three replicates. statistical significance was assessed by student’s t-test. bars assigned with different letters are significantly different (*P* < 0.05).

In order to investigate the linear RNAs removal efficiency of different enrichment method (use the delta Ct method to calculate the percentage (%) of linear RNA left after different enrichment methods [25]), we randomly selected several different circRNAs (hsa_circ_0001445, hsa_circ_0004771) and linear RNAs (GAPDH, 18S, NRIP1) for validation. As shown in Figure 2(a), convergent and divergent primers were used to detect linear and circular transcripts by qPCR, respectively. As expected, all linear RNAs significantly decreased (*P* < 0.05) in the polyA+RNase R treatment group compared with rRNA ^–^ group, but most of the circRNAs were increased (but no statistical significance (*P* > 0.05)) (Figure 2(b)). In addition, the removal efficiency of linear RNA and the enrichment effect of circRNA by using rRNA^–^+polyA+RNase R and the polyA+RNase R+ rRNA ^–^ treatment group were much greater than one using polyA+RNase R treatment group, but no statistical significance (*P* > 0.05). And the polyA+RNase R+ rRNA ^–^ treatment group could obtain higher circRNA enrichment efficiency (except for hsa_circ_0004771) and lower linear RNA content.

### The percentage of reads mapped using diverse circRNA enrichment methods

**Figure 3.**
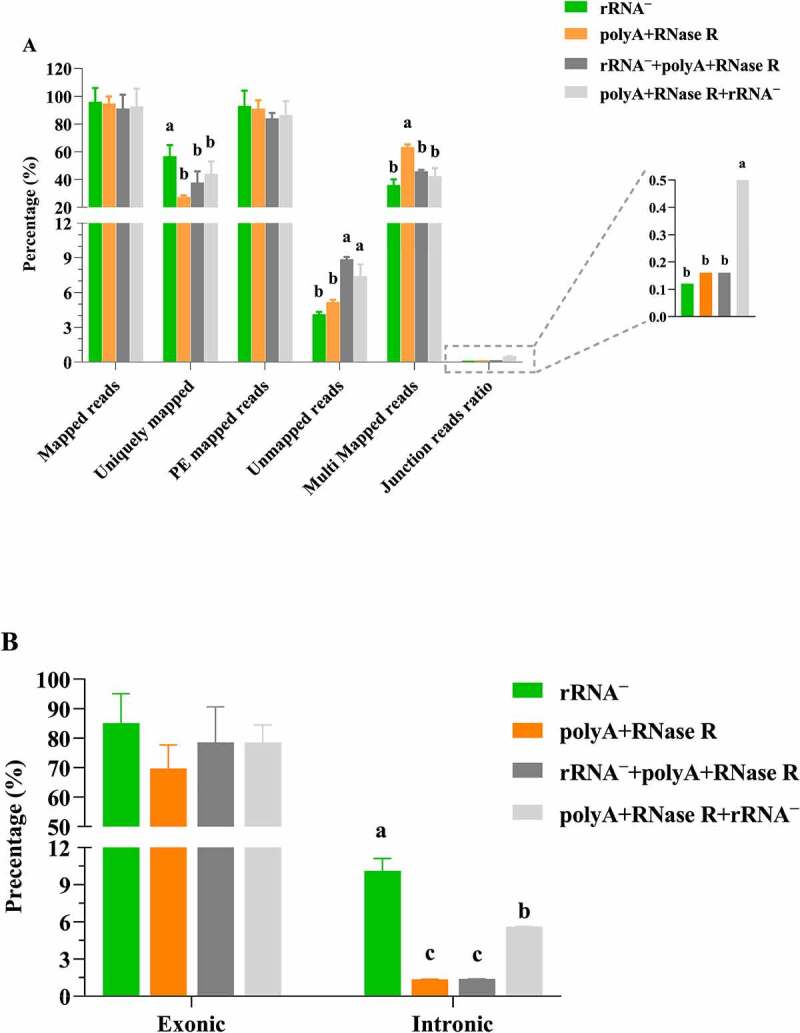
Descriptive characteristics of mapped reads. (a) read alignment and assignment rates per circRNA enrichment methods; (b) percentage of reads mapped to exonic and intronic regions per circRNA enrichment methods. each data represents the mean of three replicates. statistical significance was assessed by student’s t-test. bars assigned with different letters are significantly different (*P* < 0.05).

Note: Mapped reads: the number of reads mapped on the genome; PE mapped reads: the number of paired-end reads mapped to the genomic regions in pairs. Unmapped reads: the number of reads failed to linear mapped to the genomic regions; Multi mapped reads: the number of reads mapped to multi genomic regions.

In the present study, initial analyses focused on the basic parameters of mapped reads. All RNA-seq datasets were aligned to their reference genomes using Hisat2. Figure 3(a) and Table S2 presented the percent distributions of reads into those that aligned, reads that uniquely mapped, paired-end mapped (PE mapped), unmapped, multi mapped and junction reads ratio. Several results are observed. One is that the percent distribution of mapped reads and PE mapped are not greatly affected by circRNA enrichment methods. Another result is that the percent distribution of uniquely mapped reads is significantly higher in rRNA treatment group (*P* < 0.05) with compared to other enrichment methods, whereas the opposite trend was observed for unmapped reads. Finally, all enrichment methods produced multi mapping reads varied substantially, with polyA+RNase R group showing the largest percentage and significantly higher than other groups (*P* < 0.05). In addition, the junction reads ratio was evaluated by using diverse circRNA enrichment protocols. As was shown in Figure 3(a), the junction reads ratio was significantly lower (*P* < 0.05) in rRNA ^–^ depletion (0.12%) with compared to other enrichment protocols, and polyA+RNase R+ rRNA ^–^ (0.5%) enrichment method was higher than that to polyA+RNase R (0.16%) and rRNA^–^+polyA+RNase R (0.16%) protocols, but no statistical significance (*P* > 0.05). In Table 2, rRNA ^–^ treatment group obtained a significantly higher (*P* > 0.05) PCR duplication rate (57%), and the PCR duplication rate (44%) of polyA+RNase R+ rRNA ^–^ was higher than that the polyA+RNase R (28%) and rRNA^–^+ polyA+RNase R (38%) treatment groups.

**Table 2.** The complexity of libraries using different circRNA enrichment methods

Group	Percent_Duplication (%)	Mean ± SEM (%)
rRNA^–^_1	58.20%	(56.88 ± 1.19%)^a^
rRNA^–^_2	54.50%	
rRNA^–^_3	57.93%	
polyA+RNase R_1	28.45%	(27.70 ± 1.40%)^c^
polyA+RNase R_2	29.66%	
polyA+RNase R_3	25.00%	
rRNA^–^+polyA+RNase R_1	35.32%	(37.93 ± 1.34%)^b^
rRNA^–^+polyA+RNase R_2	38.75%	
rRNA^–^+polyA+RNase R_3	39.73%	
polyA+RNase R+ rRNA^–^_1	49.70%	(44.16 ± 3.09%)^b^
polyA+RNase R+ rRNA^–^_2	43.78%	
polyA+RNase R+ rRNA^–^_3	39.00%	

Statistical significance was assessed by Student’s t-test. Means in the different column with different letters were significantly different (*P* < 0.05).

As can be seen from Figure 3(b), rRNA ^–^ enrichment method showed the higher alignment to exonic regions, but no statistical significance (*P* > 0.05). The percentage of reads aligned to exonic regions was lower than 80% in samples prepared with polyA+RNase R (69%), rRNA^–^+ polyA+RNase R (78%) and polyA+RNase R+ rRNA ^–^ (78%). As expected, the overall percentage of reads aligning to intronic regions detected was less than 10% for diverse circRNA enrichment methods.

### The number of circRNA identification in diverse enrichment methods

**Figure 4.**
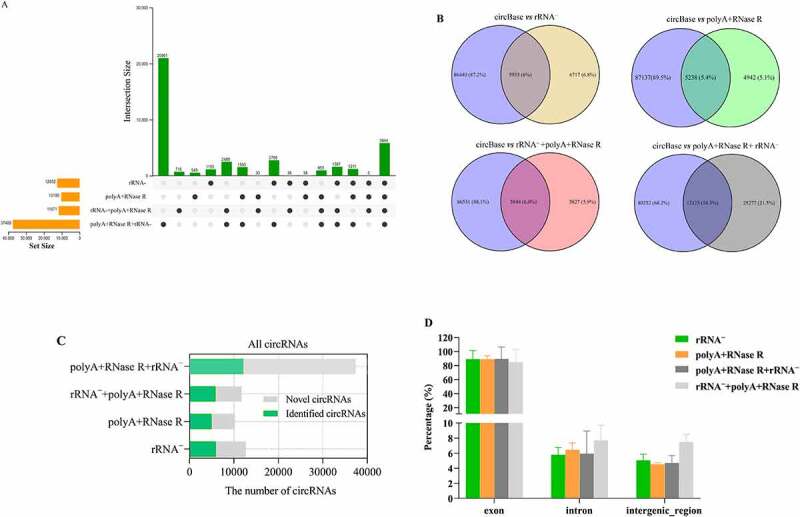
Identification and characterization of circRNAs by using four diverse enrichment methods. (a) the upset plot shows the distribution of identified circRNAs by using four diverse enrichment methods. The bar chart above represents the number of circRNAs contained in each enrichment method. The bar chart at the bottom left represents the number of identified circRNAs included in each enrichment method. The dotted line at the bottom right shows the number of identified circRNAs each enrichment method. One dot represents the circRNAs identified by one of the circRNA enrichment method. Two dots represents the circRNAs identified by two of the circRNA enrichment method. Three dots represents the circRNAs identified by three of the circRNA enrichment method. Four dots represents the circRNAs identified by four of the circRNA enrichment method; (b) The number of overlap circRNAs by comparing of diverse circRNA enrichment methods and circBase; (c) Number of identification circRNAs and novel circRNAs; (d) The distribution of circRNAs, intron circRNAs and intergenic_region circRNAs in each treatment sample. Data are shown as means ± SEM.

The clean reads were further aligned to the reference genome using the BWA-MEM algorithm, then the circRNAs were detected and quantified with at least two independent reads spanning over back-splice junction sites by using CIRI2. As presented in Figure 4(a), we identified the 12652, 10180, 11671 and 37400 circRNAs from rRNA^–^, polyA+RNase R, rRNA^–^+polyA+RNase R and polyA+RNase R+ rRNA ^–^ treatment group, respectively. The number of detected circRNAs by polyA+RNase R+ rRNA ^–^ treatment is threefold more than the circRNA number ever detected by rRNA^–^, polyA+RNase R and rRNA^–^+polyA+RNase R treatment. Of these, only a modest overlap of 5844 circRNAs was observed between all four enrichment methods.

Furthermore, to verify that we experiment identified bona fide circRNAs rather than false positives, we searched the previously published circRNAs deposited in database circBase (92,061 human circRNAs). This result found that 5935 (6%), 5238 (5.4%), 5844 (6%) and 12123 (10.3%) circRNAs have been included in the circBase after rRNA^–^, polyA+RNase R, rRNA^–^+polyA+RNase R and polyA+RNase R+ rRNA ^–^ treatment, respectively (Figure 4(b)). The 6717 (6.8%), 4842 (5.1%), 5827 (5.9%) and 25277 (21.5%) of novel circRNAs were discovered from rRNA^–^, polyA+RNase R, rRNA^–^+polyA+RNase R and polyA+RNase R+ rRNA ^–^ treatment, respectively (Figure 4(c)).

As is shown in Figure 4(d), all circRNA enrichment methods had a greater fraction of exon circRNA species, to a similar extent (except for polyA+RNase R+ rRNA ^–^ treatment group). The fraction of intergenic region circRNA species did not vary much among all the libraries. Nevertheless, the polyA+RNase R+ rRNA ^–^ treatment method had higher proportions of intron and intergenic regions circRNA species with compared to other treatment methods.

### Sensitivity, precision and reproducibility of diverse circRNA enrichment methods

**Figure 5.**
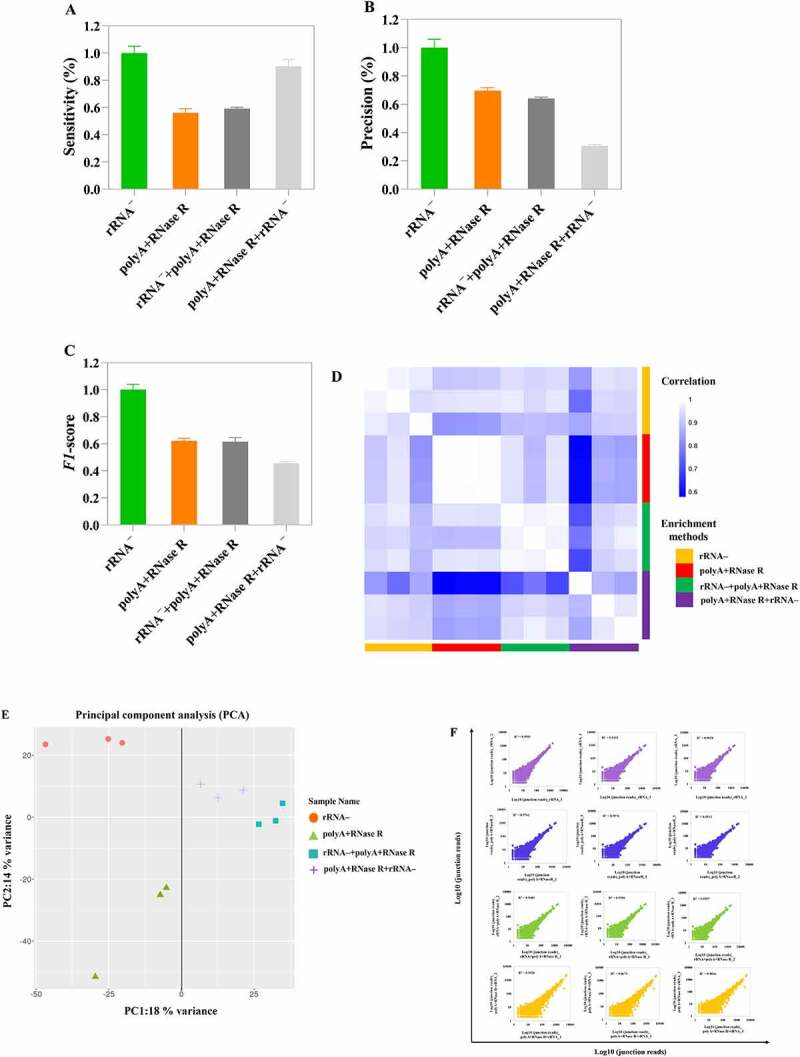
The analysis of circRNAs identification sensitivity, precision and correlation by using four diverse enrichment methods. (a) Sensitivity for detecting circRNAs at diverse enrichment methods; (b) Precision for detecting circRNAs at diverse enrichment methods; (c) *F1*-score for detecting circRNAs at diverse enrichment methods; (d) Heatmap showing Pearson correlation of log2 transformed count values (blue indicates low correlation and white indicates high correlation); (e) PCA plot show global expression pattern for each circRNA enrichment sample; (f) Scatter plots show correlation between the two replicates for each circRNA enrichment methods. *R^2^* indicates coefficient of determination.

Using the samples of rRNA ^–^ treatment as reference, we evaluated the sensitivity and precision at different circRNA enrichment methods. This results indicated that polyA+RNase R+ rRNA ^–^ circRNAs (92.4%) enrichment method had better sensitivity compared to polyA+RNase R (56.1%) and rRNA^–^+polyA+RNase R (59.15%) treatment groups (Figure 5(a)). In addition, based on the number of junction reads, polyA+RNase R+ rRNA and rRNA exhibit the highest and lowest level of sensitivity, i.e. number of reads per circRNA, respectively (18 versus 9 reads per circRNA on average, Figure S1A and S1B). However, the precision of polyA+RNase R+ rRNA ^–^ (59.15%) treatment groups was lower than that of other groups (Figure 5(b)). Furthermore, in Figure 5(c), the value of *F1* measure was lower than that of other groups in polyA+RNase R+ rRNA ^–^ treatment groups.

Additionally, we checked the reproducibility of different protocols and technical replicates. This result found that the high reproducibility was observed in different circRNA enrichment methods and technical replicates (*R^2^* > 0.8), except the polyA+RNase R+ rRNA^–^_1 (Figure 5(d-F)). Consistent with this observation, the PCA plot showed tight clustering of technical replicates (Figure 5(e)).

### The abundances of circRNAs in diverse enrichment methods

**Figure 6.**
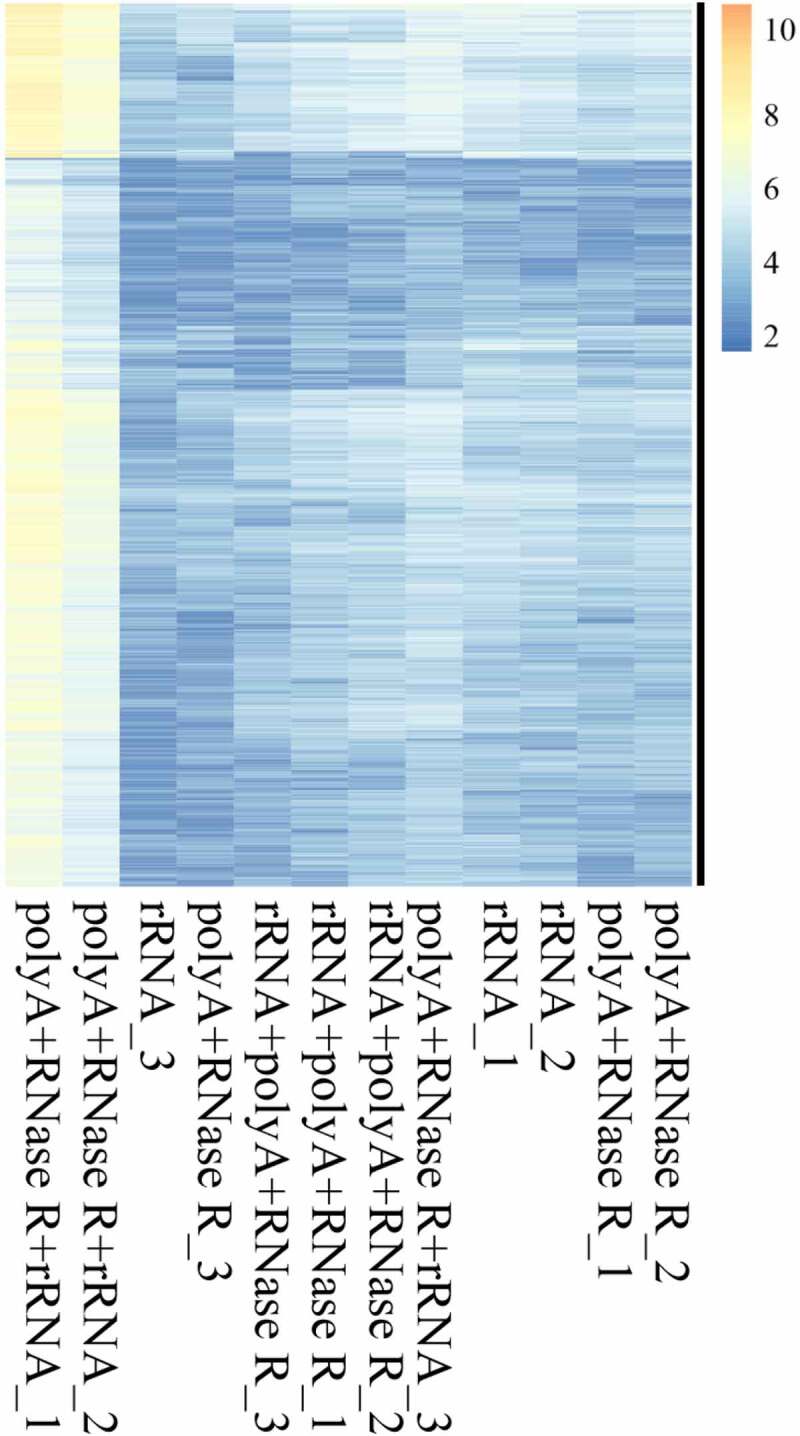
On diverse circRNA enrichment methods, the candidates circRNA of backspliced junction reads ≥ 2 were extracted. Then, these circRNAs were used in the cluster analysis by log2N processing, where N is the readcount. Each column represents detection of a sample specific methods, and each row corresponds to a circRNA.

In the present study, we normalized the junction reads (support for circRNAs) by read counts. The result indicated that the profiles differed for circRNA in different enrichment methods (Figure 6 and Table S3), with a higher abundance of circRNAs in polyA+RNase R+ rRNA ^–^ group with compared to other treatment group.

### qPCR validation of diverse circRNA enrichment methods

**Figure 7.**
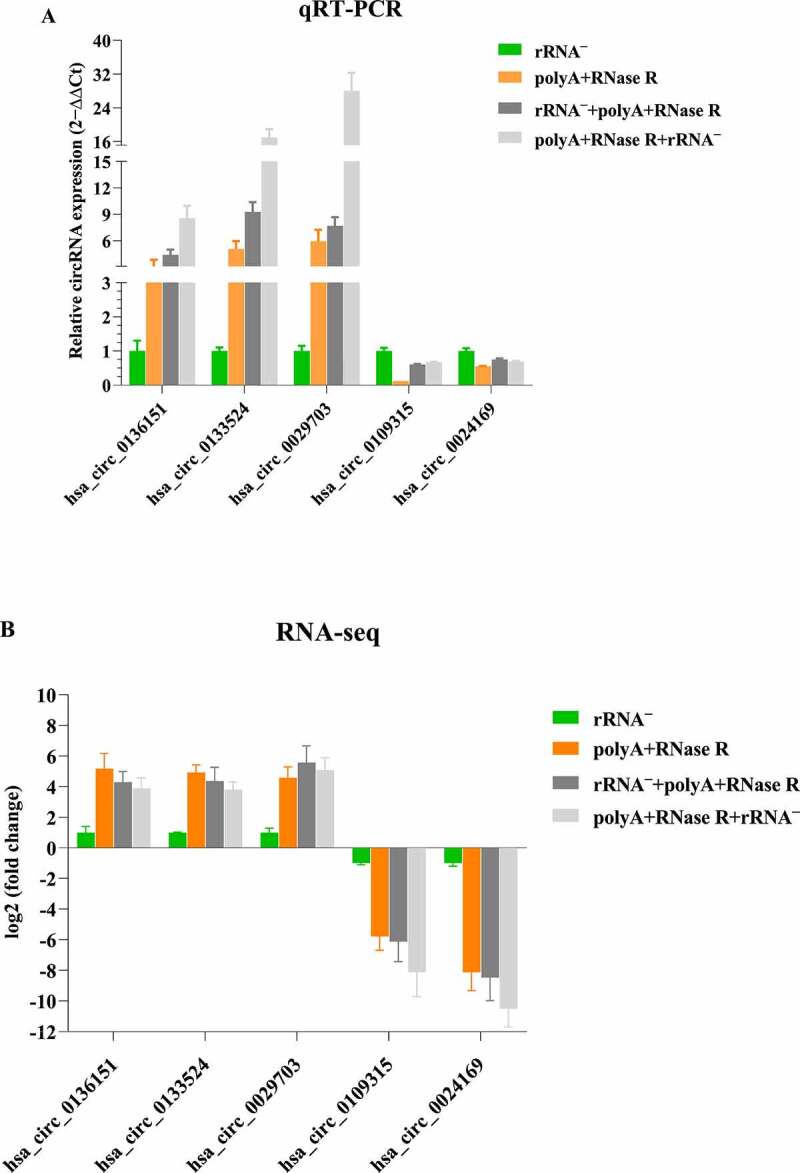
qPCR validation of diverse circRNA enrichment methods. (a) qPCR validation of five differentially expressed circRNAs in four diverse circRNA enrichment methods. (b) The RNA-seq result of five differentially expressed circRNAs by using four diverse circRNA enrichment methods. Data are shown as means ± SEM.

To confirm our identification of circRNAs, six highly expressed circRNA candidates (three up-regulations and two down-regulations) were selected for experimental validation using qPCR. A set of divergent primers (Figure 2(a) and Table 2) was designed for qPCR with GAPDH as the internal gene expression control. As is shown in Figure 7(a,b), we successfully amplified five circRNAs (83% of six candidates) from rRNA^–^, polyA+RNase R, rRNA^–^+polyA+RNase R and polyA+RNase R+ rRNA ^–^ samples.

## Discussion

As a group of much neglected noncoding RNA, circRNAs have been recently authenticated in many cells and tissues. Although tens of thousands of circRNAs have been discovered to date, it is still far from being fully study. It is due to the fact that circular RNAs are not easy to detect and enrichment from total RNA due to their covalently closed ring structure. In view of this problem, researchers developed new methodology to improve the enrichment efficiency of circRNAs during sample processing, such as rRNA depleted method, exonuclease‐enrichment approaches, RPAD method. Although new enrichment methods have improved for circRNAs enrichment efficiency, the accuracy enrichment performance of these methods have not been evaluated. Hence, in this study, we selected four commonly used circRNA enrichment methods (rRNA depletion, polyA+RNase R, rRNA^–^+polyA+RNase R and polyA+RNase R+ rRNA^–^) for comparison, and the sequencing data were systematically evaluated among them. Our results showed that polyA+RNase R+ rRNA **^–^** enrichment method obtained more circRNA number, higher sensitivity and abundance among them; polyA+RNase R method obtained higher precision. The linear RNAs can be thoroughly removed in all enrichment methods except rRNA depletion method.

To validate the digestion of linear RNAs with RNase R, we performed a limited screen of mRNAs and circRNAs by using qPCR. This result showed that linear RNAs were decreased in polyA+RNase R treatment group compared to the rRNA ^–^ treatment group, whereas the opposite was found for circRNAs. These results were consistent with previous studies; RNase R can degrade linear RNAs from total RNA pool and improve circRNA enrichment efficiency [21,22]. Furthermore, our studies found that the efficiency of linear RNAs depletion were better using rRNA^–^+polyA+RNase R and polyA+RNase R+ rRNA ^–^ enrichment methods with compared to polyA+RNase R enrichment method. This result indicated that the combination of specific linear RNA and general linear RNA removal method will be better. It was worth mentioning that the linear RNAs depletion efficiency were not remarkably difference using polyA+RNase R+ rRNA ^–^ enrichment method with compared to rRNA^–^+polyA+RNase R enrichment method. That means that the order of treatment is not different, and the depletion of rRNA is more critical. Thus, the combination of rRNA ^–^ depletion and polyA+RNase R method can be used to isolate highly purity circRNAs from total RNA pools, which increase the opportunity to detect novel circRNAs by RNA-seq.

Additionally, previous studies have been shown that only total RNA-seq data with good base quality is eligible for circRNA predictions [30]. Thus, we evaluated the data quality of each circRNA enrichment methods. This result showed that the majority of reads mapped linearly to the genome, and which was not affected of circRNA enrichment methods. These results are consistent with those reported by Ma N, et al. [31]. Additionally, in order to evaluate the mapped rate of sequencing data, we analysed the rate of uniquely mapped linearly from each circRNA enrichment methods. The results showed that the specificity and efficiency of circRNA enrichment methods were largely limited, thus leading to an extraordinarily high variance in uniquely mapped by using different circRNA methods. Furthermore, studies indicated that the junction spanning reads of circRNA typically comprise less than 0.1% of the reads generated in a total RNA-seq [32–34]. The consistent result was also observed in our current study after rRNA treatment group. However, the junction reads ratio was higher in other three enrichment protocols than that rRNA depletion protocol. It is due to the fact that RNase R effectively removes the linear RNAs, leading to an increased of junction reads ratio. More importantly, the junction reads ratio was higher in polyA+RNase R+ rRNA ^–^ (0.5%) treatment group than that polyA+RNase R (0.16%) and rRNA ^–^ +polyA+RNase R (0.16%) groups. Consequently, we believe polyA+RNase R+ rRNA ^–^ of circRNA enrichment methods might be higher detection efficiency than other three enrichment methods for circRNAs enrichment. It was worth mentioning that the junction reads ratio were higher using polyA+RNase R+ rRNA ^–^ enrichment method with compared to rRNA^–^+polyA+RNase R enrichment method. This result may be due to the different order of rRNA removal, which affects the efficiency of rRNA removal, thus resulting in the different of junction reads ratio. Furthermore, in order to evaluate the library complexity, we detected the PCR duplication rate of the read pairs as lower duplication rates usually indicate a higher complexity of the sample and better representation of RNA present in a sample [35]. We result showed that a lower PCR duplication rates for the polyA+RNase R and rRNA^–^+polyA+RNase R prepared samples as compared to samples prepared with the other enrichment protocols. A possible explanation to this observed lower PCR duplication rate may relate to the methods of these two enrichment methods.

In this study, our result showed that the number of detected circRNAs by polyA+RNase R+ rRNA ^–^ treatment is threefold more than the circRNA number ever detected by rRNA^–^, polyA+RNase R and rRNA^–^+polyA+RNase R treatment. This might be explained by the fact that polyA+RNase R+ rRNA ^–^ treatment method can more efficient at removing linear RNAs, thus leading to an increased the resolution of the analysis. This result has also been confirmed in Philips A, et al. [36] study. Nevertheless, the number of identified circRNA candidates by using polyA+RNase R and rRNA^–^+polyA+RNase R enrichment methods were not difference with compared to rRNA depletion group. This could due to the fact that polyA+RNase R and rRNA^–^+polyA+RNase R libraries the average number of reads was lower than that rRNA depletion library. In addition, previous studies have been shown that because most of the circRNA abundance is relatively low, if we want to obtain the same amount of circRNA as the common method, which need deeper sequencing depth [28,37]. This also supports the rationality of our results once again. In addition, present study indicated that only a modest overlap of 5844 circRNAs was observed between all four enrichment methods, indicating that the obtained circRNA landscape differs quite dramatically depending on the enrichment methods of choice. Furthermore, our study found that each enrichment method identified circRNA species, only a small number of circRNAs can be found in circBase. This result may be attributed to the differences in the samples, circRNA enrichment methods and identification methods. Additionally, we evaluated the percentage of circRNA species originated from exon, intron and intergenic region. This result indicated that there were no significantly differences on the number of circRNAs in the exon region by using different enrichment methods. This is similar to the exon circRNAs distribution pattern of other study [6,38]. It is mentioning that the number of identified intron circRNAs and intergenic region circRNAs by using polyA+RNase R+ rRNA ^–^ treatment method was higher than those other three methods. This result showed that polyA+RNase R+ rRNA ^–^ enrichment method could obtain more abundant non-coding regions circRNA species. Consequently, this result will be helpful for researchers to explore the types of non-coding regions circRNAs.

How well relative expression levels of the same circRNAs can be compared across samples depends on two factors. First, the frequency of circRNAs can be measured (i.e. the abundance detected in the sample). Second, with how much technical variation it is measured (i.e. with how much noise). For the first factor, we found polyA+RNase R+ rRNA ^–^ treatment group to be the best method, as expected from its high circRNA identification sensitivity. This also in part reflected in the total number of circRNAs predicted, polyA+RNase R+ rRNA ^–^ enrichment method was identified the highest number of circRNA species. For the second factor, we found the polyA+RNase R enrichment method to perform better, as expected from its high circRNA detection precision. This allowed us to translate the sensitivity and precision parameters into the practically relevant power to detect circRNA. In addition, to more accurate evaluate the performance on balancing sensitivity and precision, the *F1*-score was employed, which represents an impartial metric. Our results found that the *F1*-score were not significantly difference in each enrichment methods. Hence, we suggested that polyA+RNase R+ rRNA ^–^ enrichment method could be chosen if researchers need to obtain a relatively high number of circRNAs. Furthermore, we further evaluated the technical reproducibility of different circRNA enrichment methods. This result found that all circRNA enrichment methods obtained a higher correlation (*R^2^* > 0.8), which was even higher than of many previous results (*R^2^* < 0.6) [17,18]. Moreover, it is important to note that, in PCA analysis, the samples of polyA+RNase R treatment presented one large deviation. This result may be caused by the deviation between human factor and operation in the library construction.

Although some circRNAs have been verified to be abundantly expressed, even more highly than their linear counterparts, the vast majority of them were usually expressed at low levels [15,17,39]. The low expression circRNAs not only constitutes challenge for their identification but also raises doubts about their functions [17,39]. In our study, we found some circRNAs were detected in the rRNA depletion libraries but were not detected in other three libraries, which might suggest some circRNAs were sensitive to RNase R. In addition, some circRNAs were not detected in the rRNA depletion libraries but were detected in other three libraries, possibly due to the presence of other similar RNA species or a bias based on the elimination of rRNA depletion. The highly abundant circRNAs may have important function. There were higher abundance of circRNAs in the polyA+RNase R+ rRNA ^–^ libraries compared to other three library preparation methods. Furthermore, in order to confirm the reliability of our enrichment methods, we selected six circRNAs identified in the experiment, including three up-regulated and three down-regulated. The selected circRNAs were further evaluated using qPCR and the results successfully amplified five circRNAs (83% of six candidates) from rRNA^–^, polyA+RNase R, rRNA^–^+polyA+RNase R and polyA+RNase R+ rRNA ^–^ samples, and confirmed the reliability of the RNA-seq analysis.

## Conclusions

In summary, we systematically compared four circRNA enrichment methods and found that polyA+RNase R+ rRNA ^–^ is preferable when needs to attain a large number of circRNA species and abundance, polyA+RNase R is preferable when needs to more higher precision of identification circRNA. Overall, our results helps researchers to quickly selection a circRNA enrichment of suitable for own study among many enrichment methods, and it provides a benchmark framework for future improvements circRNA enrichment methods.

## Supplementary Material

Supplemental Material
