# Supplementary material for: Comparative analysis of circular RNA enrichment methods
Source: RNA Biol. 2021 Dec 12;19(1):55–67. doi: 10.1080/15476286.2021.2012632 (PMC8786342; doi:10.1080/15476286.2021.2012632)
Supplement: Supplemental Material [file KRNB_A_2012632_SM2771.zip › supplementary/Supplementary Tables.docx]

| **Supplementary Table S1. Summary of RNA sequencing data.** | | | | | | | | |
| --- | --- | --- | --- | --- | --- | --- | --- | --- |
| Group | Raw Base(G) | Raw Reads | Clean Base(G) | Clean Reads | Error Rate(%) | Q20(%) | Q30(%) | GC Content(%) |
| rRNA^–^_1 | 17.25 | 57,511,689 | 11.84 | 39,465,517 | 0.03 | 97.74 | 94.03 | 58.76 |
| rRNA^–^_2 | 22.04 | 73,462,013 | 14.23 | 47,440,315 | 0.03 | 97.66 | 93.97 | 60.19 |
| rRNA^–^_3 | 17.05 | 56,827,296 | 12.4 | 41,338,029 | 0.03 | 97.54 | 93.4 | 58.34 |
| polyA+RNase R_1 | 17.32 | 57,718,872 | 11.34 | 37,792,986 | 0.03 | 97.08 | 92.86 | 63.45 |
| polyA+RNase R_2 | 17.49 | 58,314,386 | 11.77 | 39,221,877 | 0.03 | 96.95 | 92.58 | 63.49 |
| polyA+RNase R_3 | 15.96 | 53,190,866 | 10.28 | 34,274,902 | 0.03 | 96.96 | 92.67 | 63.64 |
| rRNA^–^+poly+RNase R_1 | 15.62 | 52,058,445 | 12.45 | 41,509,547 | 0.03 | 96.6 | 91.81 | 64.56 |
| rRNA^–^+poly+RNase R_2 | 11.88 | 39,608,839 | 10.03 | 33,446,970 | 0.03 | 96.95 | 92.22 | 65.93 |
| rRNA^–^+poly+RNase R_3 | 12.2 | 40,659,113 | 9.8 | 32,678,265 | 0.03 | 97.1 | 92.63 | 66.12 |
| polyA+RNase R+rRNA^–^_1 | 11.09 | 36,957,063 | 10.59 | 35,290,669 | 0.03 | 96.54 | 91.44 | 63.05 |
| polyA+RNase R+rRNA^–^_2 | 12.34 | 41,132,971 | 10.95 | 36,488,394 | 0.03 | 96.42 | 91.28 | 64.6 |
| polyA+RNase R+rRNA^–^_3 | 14.51 | 48,356,751 | 12.57 | 41,900,765 | 0.03 | 96.75 | 91.86 | 61.96 |

**Supplementary Table S2. Raw data of different treatment groups sequencing.**

| Sample | Clean reads | Paired reads | Mapped reads | Uniquely mapped | PE mapped reads | Unmapped reads | Multi Mapped reads | Junction reads | Junction reads ratio |
| --- | --- | --- | --- | --- | --- | --- | --- | --- | --- |
| rRNA^–^_1 | 39,465,517 | 39,465,517 | 37,961,881 (96.19%) | 22,955,389 (58.17%) | 36,898,733 (93.5%) | 15,036,36 (3.81%) | 13,943,344 (35.33%) | 101,446 | 0.257 |
| rRNA^–^_2 | 47,440,315 | 47,440,315 | 45,177,412 (95.23%) | 25,860,520 (54.51%) | 43,709,331 (92.13%) | 22,629,03 (4.77%) | 17,848,811 (37.62%) | 11153 | 0.024 |
| rRNA^–^_3 | 41,338,029 | 41,338,029 | 39,775,451 (96.22%) | 23,946,817 (57.93%) | 38,660,214 (93.52%) | 15,625,78 (3.78%) | 14,713,397 (35.59%) | 29667 | 0.072 |
| **Average** | **42,747,954** | **42,747,954** | **40,971,581 (95.88%)** | **24,254,242 (56.87%)** | **39,756,093 (93.05%)** | **17,763,72 (4.12%)** | **15,501,851 (36.18%)** | **47422** | **0.12** |
| polyA+RNase R_1 | 37,792,986 | 37,792,986 | 35,880,660 (94.94%) | 10,751,311 (28.45%) | 34,473,285 (91.22%) | 19,123,26 (5.06%) | 23,721,974 (62.77%) | 65594 | 0.17 |
| polyA+RNase R_2 | 39,221,877 | 39,221,877 | 37,225,483 (94.91%) | 11,632,495 (29.66%) | 35,751,021 (91.15%) | 19,963,94 (5.09%) | 24,118,526 (61.49%) | 74347 | 0.19 |
| polyA+RNase R_3 | 34,274,902 | 34,274,902 | 32,434,339 (94.63%) | 85,661,05 (24.99%) | 31,097,896 (90.73%) | 18,405,63 (5.37%) | 22,531,791 (65.74%) | 36934 | 0.11 |
| **Average** | **37,096,588** | **37,096,588** | **35,180,161(94.8%)** | **11,191,903(27.27%)** | **33,774,067 (91.03%)** | **19,164,28 (5.17%)** | **23,457,430 (63.3%)** | **58958** | **0.16** |
| rRNA^–^+poly+RNase R_1 | 41,509,547 | 41,509,547 | 37,312,931 (89.89%) | 14,660,845 (35.32%) | 33,972,325 (81.84 %) | 41,966,16 (10.11%) | 19,311,480 (46.52 %) | 74175 | 0.18 |
| rRNA^–^+poly+RNase R_2 | 33,446,970 | 33,446,970 | 30,523,704 (91.26%) | 12,961,497 (38.75%) | 28,037,113 (83.82%) | 29,232,66 (8.74%) | 15,075,616 (45.07%) | 89856 | 0.27 |
| rRNA^–^+poly+RNase R_3 | 32,678,265 | 32,678,265 | 30,142,432 (92.24%) | 12,983,580 (39.73%) | 28,184,711 (86.25%) | 25,358,33 (7.76%) | 15,201,131 (46.52%) | 10988 | 0.03 |
| **Average** | **35,878,261** | **35,878,261** | **32,659,689(91.13%)** | **13,535,307 (37.93%)** | **30,064,716 (83.97%)** | **32,185,72 (8.87%)** | **16,529,409 (46.04%)** | **58340** | **0.16** |
| polyA+RNase R+rRNA^–^_1 | 35,290,669 | 35,290,669 | 32,139,212 (91.07%) | 17,542,197 (49.71%) | 29,682,227 (84.11%) | 31,514,57 (8.93%) | 12,140,080 (34.40%) | 246215 | 0.70 |
| polyA+RNase R+rRNA^–^_2 | 36,488,394 | 36,488,394 | 33,547,429 (91.94%) | 15,828,356 (43.38%) | 30,733,007 (84.23%) | 29,409,65 (8.06%) | 14,904,651 (40.85%) | 185652 | 0.51 |
| polyA+RNase R+rRNA^–^_3 | 41,900,765 | 41,900,765 | 39,688,404 (94.72%) | 16,360,218 (39.05%) | 38,038,696 (90.79%) | 22,123,61 (5.28%) | 21,678,478 (51.74%) | 126250 | 0.30 |
| **Average** | **37,893,276** | **37,893,276** | **35,125,015(92.58%)** | **16,576,924 (44.05%)** | **32,817,977 (86.38%)** | **27,682,61 (7.42%)** | **16,241,070 (42.33%)** | **186039** | **0.50** |

Note: Clean reads: the number of reads after preprocessing; Paired reads: the number of paie-end reads mapped to the genomic regions; Mapped reads: the number of reads mapped on the genome; PE mapped reads: the number of paied-end reads mapped to the genomic regions in pairs. Unmapped reads: the number of reads failed to linear mapped to the genomic regions; Multi mapped reads: the number of reads mapped to multi genomic regions;

**Supplemental Tables S3. Abundances of circRNAs in different circRNA enrichment methods**

| circRNA_ID | circBase_circRNA_ID | rRNA^–^_1 | rRNA^–^_2 | rRNA^–^_3 | polyA+RNase R_1 | polyA+RNase R_2 | polyA+RNase R_3 | rRNA^–^+poly+RNase R_1 | rRNA^–^+poly+RNase R_2 | rRNA^–^+poly+RNase R_3 | polyA+RNase R+rRNA^–^_1 | polyA+RNase R+rRNA^–^_2 | polyA+RNase R+rRNA^–^_3 |
| --- | --- | --- | --- | --- | --- | --- | --- | --- | --- | --- | --- | --- | --- |
| chrY:2821950\|2829687 | hsa_circ_0001953 | 24 | 18 | 5 | 11 | 11 | 5 | 30 | 29 | 10 | 214 | 99 | 38 |
| chrY:22669238\|22683186 | #N/A | 10 | 6 | 3 | 9 | 3 | 3 | 5 | 9 | 6 | 85 | 31 | 7 |
| chrY:21749096\|21749393 | hsa_circ_0009024 | 12 | 12 | 6 | 5 | 5 | 6 | 10 | 18 | 14 | 99 | 50 | 25 |
| chrX:84322133\|84329397 | #N/A | 24 | 19 | 7 | 13 | 17 | 8 | 11 | 17 | 8 | 198 | 70 | 19 |
| chrX:79962926\|79975155 | hsa_circ_0001936 | 6 | 5 | 2 | 3 | 5 | 5 | 4 | 8 | 3 | 56 | 22 | 9 |
| chrX:77270159\|77275895 | hsa_circ_0001934 | 77 | 62 | 19 | 46 | 42 | 29 | 95 | 122 | 67 | 702 | 308 | 141 |
| chrX:76907604\|76912143 | hsa_circ_0001932 | 56 | 42 | 21 | 32 | 21 | 18 | 10 | 12 | 11 | 108 | 21 | 8 |
| chrX:74357770\|74362801 | #N/A | 21 | 13 | 3 | 15 | 8 | 6 | 11 | 13 | 19 | 113 | 40 | 12 |
| chrX:67731691\|67742759 | hsa_circ_0004379 | 21 | 15 | 8 | 20 | 27 | 9 | 30 | 32 | 10 | 237 | 117 | 28 |
| chrX:54013511\|54014377 | hsa_circ_0009069 | 8 | 13 | 4 | 7 | 5 | 3 | 7 | 12 | 4 | 36 | 31 | 5 |
| chrX:53641495\|53642796 | hsa_circ_0007817 | 37 | 43 | 7 | 23 | 24 | 13 | 34 | 46 | 24 | 252 | 114 | 54 |
| chrX:53430498\|53430825 | hsa_circ_0001921 | 144 | 150 | 52 | 115 | 132 | 91 | 125 | 144 | 111 | 923 | 582 | 234 |
| chrX:47705504\|47755339 | hsa_circ_0006364 | 3 | 5 | 3 | 6 | 3 | 5 | 5 | 8 | 7 | 36 | 26 | 14 |
| chrX:44383248\|44386611 | hsa_circ_0007290 | 13 | 14 | 3 | 8 | 7 | 4 | 7 | 18 | 12 | 147 | 55 | 23 |
| chrX:17151947\|17157084 | hsa_circ_0089973 | 10 | 10 | 4 | 14 | 12 | 7 | 12 | 12 | 13 | 101 | 55 | 16 |
| chrX:17121841\|17157084 | hsa_circ_0089972 | 5 | 8 | 8 | 8 | 7 | 8 | 8 | 9 | 6 | 74 | 33 | 7 |
| chrX:154018229\|154020560 | hsa_circ_0001952 | 8 | 7 | 2 | 6 | 9 | 3 | 4 | 9 | 14 | 48 | 38 | 16 |
| chrX:149962164\|149984551 | hsa_circ_0001948 | 12 | 17 | 4 | 14 | 16 | 7 | 26 | 35 | 17 | 151 | 111 | 34 |
| chrX:147743429\|147744289 | hsa_circ_0001947 | 38 | 35 | 11 | 10 | 10 | 2 | 7 | 6 | 3 | 138 | 76 | 13 |
| chrX:13684436\|13698717 | hsa_circ_0007717 | 31 | 26 | 17 | 33 | 20 | 10 | 39 | 46 | 35 | 222 | 145 | 75 |
| chrX:13684436\|13686502 | hsa_circ_0002871 | 19 | 7 | 2 | 8 | 11 | 4 | 8 | 27 | 12 | 126 | 76 | 36 |
| chrX:123155217\|123164975 | #N/A | 10 | 9 | 8 | 3 | 10 | 2 | 7 | 4 | 4 | 84 | 24 | 12 |
| chrX:118774655\|118787003 | hsa_circ_0008350 | 13 | 8 | 9 | 4 | 14 | 2 | 17 | 29 | 16 | 101 | 86 | 26 |
| chrX:117788565\|117788924 | hsa_circ_0007733 | 8 | 5 | 3 | 11 | 15 | 4 | 12 | 8 | 6 | 81 | 38 | 3 |
| chrX:117718698\|117724265 | hsa_circ_0091382 | 5 | 7 | 6 | 8 | 11 | 4 | 3 | 11 | 4 | 89 | 23 | 7 |
| chrX:106410879\|106418423 | hsa_circ_0091291 | 7 | 14 | 3 | 7 | 6 | 4 | 4 | 6 | 5 | 24 | 19 | 8 |
| chrX:106394086\|106396733 | #N/A | 11 | 14 | 2 | 11 | 12 | 4 | 24 | 23 | 15 | 166 | 59 | 14 |
| chrX:106390973\|106396733 | hsa_circ_0091288 | 13 | 7 | 3 | 7 | 11 | 5 | 12 | 16 | 13 | 116 | 62 | 29 |
| chrX:106331666\|106332069 | hsa_circ_0002995 | 9 | 10 | 3 | 14 | 14 | 3 | 13 | 22 | 8 | 138 | 58 | 30 |
| chrX:10031485\|10066619 | hsa_circ_0001910 | 18 | 6 | 8 | 9 | 8 | 5 | 13 | 11 | 9 | 115 | 48 | 10 |
| chr9:99220661\|99233376 | hsa_circ_0087631 | 4 | 2 | 5 | 5 | 2 | 2 | 4 | 7 | 9 | 22 | 21 | 14 |
| chr9:98740343\|98766983 | hsa_circ_0008720 | 27 | 38 | 9 | 16 | 15 | 5 | 25 | 26 | 16 | 197 | 111 | 42 |
| chr9:96233423\|96261168 | hsa_circ_0001875 | 32 | 35 | 10 | 21 | 33 | 21 | 26 | 22 | 25 | 205 | 119 | 34 |
| chr9:96233423\|96238620 | hsa_circ_0008193 | 23 | 13 | 8 | 17 | 37 | 19 | 41 | 34 | 20 | 180 | 89 | 39 |
| chr9:88957990\|88961395 | #N/A | 12 | 11 | 3 | 7 | 7 | 4 | 3 | 8 | 9 | 96 | 49 | 18 |
| chr9:88920107\|88924932 | hsa_circ_0001869 | 29 | 23 | 13 | 21 | 23 | 11 | 36 | 37 | 23 | 337 | 115 | 41 |
| chr9:88307604\|88327481 | hsa_circ_0007351 | 41 | 22 | 14 | 20 | 25 | 20 | 6 | 6 | 3 | 116 | 46 | 10 |
| chr9:88292351\|88327481 | hsa_circ_0006687 | 18 | 15 | 3 | 10 | 7 | 7 | 17 | 22 | 19 | 140 | 72 | 28 |
| chr9:88284400\|88327481 | hsa_circ_0087391 | 25 | 42 | 15 | 10 | 20 | 7 | 21 | 26 | 7 | 276 | 113 | 33 |
| chr9:88233898\|88248289 | hsa_circ_0001868 | 16 | 25 | 11 | 6 | 9 | 4 | 4 | 17 | 2 | 115 | 55 | 14 |
| chr9:86297866\|86301070 | hsa_circ_0005142 | 107 | 75 | 17 | 70 | 78 | 51 | 74 | 47 | 22 | 886 | 331 | 134 |
| chr9:86294690\|86301070 | hsa_circ_0008207 | 66 | 25 | 15 | 39 | 24 | 17 | 70 | 65 | 39 | 593 | 257 | 91 |
| chr9:86293356\|86301070 | hsa_circ_0087357 | 81 | 65 | 24 | 31 | 28 | 15 | 50 | 32 | 15 | 487 | 193 | 67 |
| chr9:86293356\|86297981 | hsa_circ_0001866 | 27 | 17 | 9 | 16 | 18 | 6 | 25 | 35 | 16 | 183 | 100 | 36 |
| chr9:86274695\|86284242 | hsa_circ_0008038 | 13 | 3 | 4 | 6 | 4 | 3 | 11 | 7 | 5 | 63 | 29 | 16 |
| chr9:80409379\|80412564 | hsa_circ_0006459 | 7 | 11 | 7 | 10 | 11 | 4 | 12 | 15 | 7 | 63 | 30 | 9 |
| chr9:79829245\|79842401 | hsa_circ_0087243 | 30 | 36 | 9 | 12 | 7 | 6 | 9 | 10 | 7 | 159 | 58 | 15 |
| chr9:77631184\|77632364 | hsa_circ_0004189 | 29 | 15 | 8 | 13 | 15 | 6 | 23 | 20 | 17 | 245 | 67 | 33 |
| chr9:77611363\|77632364 | hsa_circ_0087220 | 6 | 4 | 8 | 6 | 3 | 3 | 13 | 9 | 2 | 83 | 17 | 7 |
| chr9:6420912\|6434173 | hsa_circ_0002359 | 20 | 22 | 8 | 20 | 15 | 16 | 30 | 27 | 7 | 238 | 158 | 34 |
| chr9:6420912\|6421142 | hsa_circ_0086296 | 15 | 8 | 4 | 9 | 12 | 8 | 8 | 7 | 8 | 67 | 37 | 10 |
| chr9:4860125\|4860901 | hsa_circ_0007592 | 52 | 50 | 16 | 23 | 24 | 18 | 29 | 31 | 18 | 364 | 164 | 50 |
| chr9:4823548\|4833228 | hsa_circ_0006134 | 7 | 22 | 2 | 14 | 4 | 6 | 8 | 15 | 6 | 128 | 55 | 13 |
| chr9:4823548\|4827033 | hsa_circ_0006345 | 49 | 60 | 10 | 40 | 38 | 23 | 38 | 15 | 16 | 328 | 202 | 66 |
| chr9:37424842\|37426651 | hsa_circ_0001861 | 43 | 38 | 11 | 41 | 42 | 10 | 57 | 64 | 35 | 310 | 219 | 79 |
| chr9:37126309\|37147442 | #N/A | 20 | 8 | 5 | 5 | 5 | 6 | 8 | 7 | 5 | 111 | 25 | 13 |
| chr9:37126309\|37126939 | hsa_circ_0001860 | 43 | 35 | 16 | 35 | 29 | 16 | 30 | 55 | 24 | 305 | 162 | 58 |
| chr9:36597219\|36643080 | hsa_circ_0007658 | 5 | 7 | 7 | 5 | 7 | 3 | 8 | 10 | 7 | 98 | 40 | 25 |
| chr9:3647338\|3651867 | #N/A | 51 | 27 | 10 | 30 | 39 | 36 | 12 | 14 | 17 | 328 | 133 | 45 |
| chr9:36375931\|36390613 | hsa_circ_0001856 | 14 | 26 | 7 | 27 | 15 | 9 | 24 | 39 | 16 | 175 | 89 | 20 |
| chr9:36375931\|36376124 | hsa_circ_0001855 | 21 | 15 | 6 | 24 | 19 | 14 | 9 | 12 | 4 | 53 | 36 | 13 |
| chr9:36369716\|36390613 | hsa_circ_0087024 | 20 | 13 | 8 | 16 | 19 | 3 | 9 | 19 | 9 | 115 | 96 | 29 |
| chr9:36369716\|36376124 | hsa_circ_0087023 | 19 | 22 | 11 | 17 | 30 | 12 | 25 | 40 | 21 | 159 | 128 | 42 |
| chr9:3630938\|3651867 | #N/A | 6 | 9 | 3 | 3 | 9 | 5 | 7 | 11 | 7 | 145 | 45 | 27 |
| chr9:3488776\|3490345 | #N/A | 12 | 11 | 2 | 7 | 5 | 2 | 3 | 4 | 8 | 70 | 29 | 7 |
| chr9:33996221\|34017187 | hsa_circ_0003410 | 47 | 37 | 9 | 26 | 30 | 7 | 24 | 27 | 15 | 361 | 128 | 43 |
| chr9:33996221\|33998862 | hsa_circ_0086736 | 13 | 11 | 2 | 14 | 4 | 8 | 3 | 2 | 3 | 86 | 51 | 11 |
| chr9:33986758\|34017187 | hsa_circ_0086735 | 73 | 53 | 31 | 16 | 18 | 14 | 5 | 3 | 4 | 256 | 49 | 12 |
| chr9:33971649\|33973235 | hsa_circ_0001851 | 98 | 92 | 25 | 92 | 91 | 67 | 124 | 138 | 82 | 1028 | 566 | 226 |
| chr9:33960824\|33973235 | hsa_circ_0001850 | 22 | 19 | 9 | 22 | 28 | 10 | 26 | 30 | 13 | 146 | 92 | 35 |
| chr9:33960824\|33963789 | hsa_circ_0001849 | 355 | 290 | 144 | 363 | 381 | 219 | 222 | 139 | 96 | 5158 | 1948 | 419 |
| chr9:33953283\|33963789 | hsa_circ_0001847 | 19 | 14 | 5 | 14 | 18 | 4 | 17 | 28 | 16 | 143 | 90 | 20 |
| chr9:33953283\|33956144 | hsa_circ_0003945 | 22 | 20 | 7 | 15 | 25 | 9 | 15 | 16 | 17 | 126 | 75 | 20 |
| chr9:33948372\|33956144 | hsa_circ_0007367 | 65 | 75 | 19 | 91 | 100 | 50 | 102 | 96 | 83 | 633 | 366 | 139 |
| chr9:33948372\|33953472 | hsa_circ_0003496 | 66 | 65 | 19 | 96 | 84 | 34 | 81 | 100 | 41 | 474 | 325 | 116 |
| chr9:33944363\|33956144 | hsa_circ_0001846 | 32 | 17 | 4 | 26 | 27 | 12 | 19 | 18 | 8 | 87 | 68 | 26 |
| chr9:33932560\|33933644 | #N/A | 7 | 5 | 3 | 31 | 42 | 32 | 4 | 8 | 5 | 4 | 9 | 2 |
| chr9:33932560\|33933626 | hsa_circ_0086694 | 20 | 20 | 4 | 115 | 114 | 74 | 22 | 15 | 14 | 29 | 32 | 9 |
| chr9:33271150\|33278223 | hsa_circ_0003575 | 28 | 23 | 12 | 13 | 24 | 17 | 26 | 33 | 31 | 232 | 115 | 33 |
| chr9:17330630\|17342442 | hsa_circ_0086422 | 28 | 25 | 14 | 13 | 10 | 10 | 8 | 15 | 14 | 213 | 89 | 31 |
| chr9:139115853\|139118720 | #N/A | 66 | 63 | 18 | 63 | 61 | 31 | 36 | 38 | 32 | 353 | 171 | 83 |
| chr9:139115609\|139118720 | #N/A | 89 | 87 | 35 | 93 | 116 | 43 | 108 | 113 | 69 | 799 | 412 | 189 |
| chr9:138773479\|138774924 | hsa_circ_0001900 | 33 | 23 | 8 | 18 | 17 | 6 | 37 | 55 | 10 | 143 | 123 | 49 |
| chr9:136277419\|136278041 | hsa_circ_0089371 | 8 | 4 | 3 | 5 | 7 | 2 | 6 | 15 | 6 | 35 | 7 | 19 |
| chr9:134888551\|134889854 | hsa_circ_0006825 | 30 | 19 | 6 | 17 | 17 | 10 | 20 | 6 | 7 | 154 | 71 | 10 |
| chr9:134381501\|134381840 | hsa_circ_0001897 | 13 | 23 | 3 | 15 | 16 | 9 | 10 | 7 | 4 | 115 | 44 | 13 |
| chr9:131271155\|131277918 | hsa_circ_0002675 | 20 | 11 | 3 | 9 | 16 | 2 | 14 | 8 | 12 | 111 | 57 | 21 |
| chr9:131235192\|131236020 | #N/A | 7 | 6 | 4 | 9 | 7 | 8 | 7 | 11 | 11 | 49 | 35 | 17 |
| chr9:130947826\|130950213 | hsa_circ_0088744 | 3 | 7 | 2 | 9 | 14 | 10 | 10 | 6 | 5 | 29 | 16 | 5 |
| chr9:128691934\|128697886 | hsa_circ_0004804 | 11 | 9 | 3 | 10 | 19 | 5 | 17 | 29 | 9 | 153 | 81 | 16 |
| chr9:128059708\|128061385 | #N/A | 6 | 5 | 2 | 5 | 3 | 3 | 12 | 10 | 6 | 55 | 22 | 9 |
| chr9:127670656\|127674305 | hsa_circ_0002883 | 47 | 46 | 17 | 24 | 31 | 32 | 15 | 18 | 16 | 138 | 103 | 32 |
| chr9:126519982\|126641300 | hsa_circ_0002544 | 16 | 7 | 4 | 14 | 17 | 4 | 12 | 8 | 8 | 107 | 59 | 26 |
| chr9:126519982\|126531842 | hsa_circ_0088474 | 36 | 25 | 3 | 16 | 25 | 15 | 2 | 4 | 2 | 168 | 106 | 17 |
| chr9:126214555\|126217058 | #N/A | 54 | 56 | 34 | 50 | 56 | 24 | 35 | 26 | 16 | 406 | 208 | 41 |
| chr9:125932181\|125936121 | hsa_circ_0088425 | 6 | 4 | 2 | 10 | 7 | 5 | 10 | 15 | 5 | 88 | 39 | 13 |
| chr9:123924134\|123928458 | hsa_circ_0088333 | 24 | 12 | 6 | 9 | 4 | 9 | 7 | 13 | 5 | 92 | 37 | 13 |
| chr9:115030329\|115060196 | hsa_circ_0003500 | 12 | 7 | 2 | 12 | 12 | 7 | 16 | 11 | 6 | 113 | 51 | 24 |
| chr9:115013209\|115015068 | hsa_circ_0088072 | 15 | 11 | 4 | 8 | 25 | 10 | 18 | 15 | 5 | 117 | 50 | 24 |
| chr9:114911524\|114919893 | hsa_circ_0088062 | 23 | 15 | 9 | 6 | 7 | 6 | 20 | 19 | 11 | 88 | 54 | 22 |
| chr9:114842354\|114860942 | hsa_circ_0088041 | 16 | 9 | 4 | 3 | 4 | 6 | 12 | 24 | 10 | 119 | 68 | 27 |
| chr9:114840818\|114842445 | hsa_circ_0088036 | 13 | 17 | 6 | 7 | 21 | 5 | 20 | 18 | 14 | 162 | 94 | 26 |
| chr9:114148657\|114154104 | hsa_circ_0001882 | 23 | 23 | 5 | 14 | 12 | 4 | 20 | 33 | 15 | 240 | 109 | 50 |
| chr9:111812563\|111812972 | hsa_circ_0087905 | 25 | 28 | 9 | 12 | 18 | 18 | 21 | 18 | 11 | 149 | 55 | 22 |
| chr9:110062422\|110074018 | hsa_circ_0008812 | 29 | 24 | 10 | 30 | 23 | 10 | 36 | 43 | 20 | 294 | 134 | 48 |
| chr9:107513237\|107521452 | #N/A | 16 | 4 | 3 | 3 | 3 | 6 | 8 | 5 | 5 | 56 | 41 | 8 |
| chr9:102677460\|102691125 | hsa_circ_0004825 | 11 | 24 | 3 | 14 | 6 | 9 | 8 | 5 | 5 | 125 | 60 | 9 |
| chr9:100780571\|100788733 | hsa_circ_0004036 | 35 | 21 | 5 | 25 | 25 | 13 | 29 | 18 | 10 | 148 | 95 | 25 |
| chr9:100756913\|100760960 | hsa_circ_0006609 | 40 | 36 | 18 | 26 | 34 | 12 | 16 | 25 | 20 | 371 | 162 | 55 |
| chr8:99718695\|99719539 | hsa_circ_0004592 | 15 | 8 | 3 | 2 | 6 | 9 | 9 | 8 | 6 | 141 | 44 | 29 |
| chr8:98725890\|98735263 | hsa_circ_0002946 | 10 | 17 | 4 | 16 | 6 | 12 | 23 | 22 | 11 | 179 | 90 | 29 |
| chr8:98725890\|98731417 | hsa_circ_0003117 | 15 | 11 | 2 | 8 | 11 | 7 | 8 | 6 | 3 | 69 | 23 | 5 |
| chr8:98698896\|98703416 | hsa_circ_0002961 | 5 | 9 | 2 | 2 | 2 | 2 | 4 | 4 | 2 | 59 | 20 | 9 |
| chr8:95897295\|95897786 | hsa_circ_0008608 | 14 | 28 | 8 | 19 | 20 | 11 | 12 | 10 | 5 | 149 | 71 | 27 |
| chr8:95547067\|95556170 | hsa_circ_0008316 | 8 | 4 | 2 | 7 | 6 | 4 | 8 | 7 | 4 | 75 | 32 | 13 |
| chr8:93929157\|93941802 | hsa_circ_0004561 | 7 | 2 | 3 | 4 | 4 | 2 | 14 | 16 | 5 | 86 | 35 | 10 |
| chr8:74585342\|74601048 | hsa_circ_0001811 | 34 | 27 | 15 | 32 | 47 | 15 | 41 | 47 | 34 | 341 | 141 | 52 |
| chr8:71126138\|71128999 | #N/A | 126 | 80 | 31 | 72 | 69 | 43 | 62 | 54 | 40 | 775 | 328 | 105 |
| chr8:71071740\|71075089 | hsa_circ_0001810 | 28 | 34 | 4 | 22 | 36 | 10 | 18 | 42 | 28 | 195 | 109 | 65 |
| chr8:68044186\|68049838 | hsa_circ_0003388 | 31 | 16 | 10 | 12 | 8 | 9 | 11 | 10 | 12 | 130 | 49 | 16 |
| chr8:68018140\|68028357 | hsa_circ_0001806 | 26 | 11 | 5 | 16 | 14 | 7 | 14 | 24 | 8 | 111 | 65 | 18 |
| chr8:62593527\|62596747 | hsa_circ_0084615 | 19 | 11 | 2 | 14 | 13 | 12 | 21 | 22 | 27 | 118 | 65 | 23 |
| chr8:52773421\|52773806 | hsa_circ_0001802 | 27 | 19 | 9 | 7 | 15 | 11 | 16 | 10 | 7 | 247 | 46 | 14 |
| chr8:52773405\|52773806 | hsa_circ_0001801 | 618 | 473 | 230 | 251 | 231 | 270 | 238 | 253 | 175 | 4610 | 1061 | 459 |
| chr8:48612965\|48614577 | hsa_circ_0001799 | 2 | 2 | 3 | 7 | 8 | 4 | 5 | 5 | 3 | 29 | 10 | 3 |
| chr8:48308936\|48320523 | hsa_circ_0001798 | 61 | 58 | 23 | 34 | 45 | 19 | 55 | 42 | 37 | 690 | 200 | 60 |
| chr8:48196617\|48206619 | hsa_circ_0002157 | 6 | 5 | 4 | 6 | 7 | 2 | 12 | 14 | 6 | 73 | 25 | 16 |
| chr8:42914235\|42919358 | hsa_circ_0001796 | 39 | 36 | 10 | 29 | 36 | 19 | 22 | 18 | 10 | 193 | 89 | 24 |
| chr8:42761316\|42785315 | hsa_circ_0003078 | 8 | 7 | 3 | 6 | 2 | 8 | 2 | 2 | 2 | 49 | 8 | 3 |
| chr8:42183488\|42186732 | hsa_circ_0001793 | 18 | 15 | 12 | 11 | 22 | 9 | 10 | 15 | 7 | 72 | 79 | 20 |
| chr8:41561559\|41571772 | hsa_circ_0084084 | 10 | 15 | 2 | 6 | 11 | 3 | 12 | 21 | 10 | 44 | 55 | 25 |
| chr8:41561559\|41563759 | hsa_circ_0084083 | 12 | 15 | 3 | 19 | 17 | 6 | 14 | 29 | 15 | 140 | 72 | 29 |
| chr8:41550166\|41550719 | #N/A | 12 | 12 | 5 | 17 | 13 | 8 | 22 | 30 | 15 | 122 | 60 | 19 |
| chr8:41519319\|41521260 | hsa_circ_0007366 | 10 | 9 | 6 | 15 | 15 | 9 | 4 | 8 | 12 | 90 | 54 | 16 |
| chr8:41518948\|41519459 | hsa_circ_0001792 | 8 | 17 | 2 | 28 | 35 | 17 | 18 | 37 | 23 | 156 | 140 | 40 |
| chr8:37971710\|37978667 | hsa_circ_0006302 | 3 | 2 | 2 | 14 | 7 | 2 | 4 | 14 | 5 | 49 | 31 | 12 |
| chr8:37971710\|37976881 | hsa_circ_0001790 | 262 | 298 | 103 | 198 | 208 | 131 | 342 | 407 | 239 | 2952 | 1133 | 454 |
| chr8:37967897\|37968351 | hsa_circ_0083952 | 19 | 16 | 7 | 13 | 19 | 10 | 2 | 6 | 2 | 100 | 55 | 23 |
| chr8:37734627\|37735069 | hsa_circ_0001789 | 27 | 22 | 11 | 25 | 24 | 8 | 39 | 47 | 19 | 227 | 157 | 66 |
| chr8:37623044\|37623873 | hsa_circ_0001788 | 318 | 230 | 67 | 208 | 229 | 134 | 100 | 113 | 92 | 1521 | 1011 | 368 |
| chr8:30034642\|30038146 | hsa_circ_0001786 | 12 | 7 | 3 | 7 | 7 | 4 | 10 | 9 | 4 | 61 | 35 | 6 |
| chr8:26265516\|26268010 | #N/A | 9 | 7 | 3 | 4 | 6 | 4 | 6 | 8 | 9 | 74 | 31 | 12 |
| chr8:21835281\|21837714 | #N/A | 12 | 20 | 4 | 22 | 22 | 13 | 12 | 2 | 4 | 93 | 45 | 8 |
| chr8:21832181\|21845368 | #N/A | 11 | 19 | 3 | 7 | 9 | 2 | 10 | 6 | 6 | 53 | 22 | 15 |
| chr8:21832181\|21837714 | #N/A | 136 | 77 | 37 | 99 | 111 | 82 | 90 | 130 | 60 | 1070 | 531 | 161 |
| chr8:21832181\|21835354 | #N/A | 18 | 17 | 7 | 44 | 61 | 18 | 32 | 27 | 21 | 217 | 101 | 23 |
| chr8:21832181\|21834032 | hsa_circ_0083530 | 3 | 6 | 3 | 13 | 9 | 10 | 11 | 11 | 9 | 67 | 19 | 13 |
| chr8:142264088\|142264728 | hsa_circ_0001829 | 31 | 29 | 11 | 21 | 18 | 18 | 25 | 33 | 19 | 272 | 124 | 41 |
| chr8:141595218\|141616013 | hsa_circ_0007313 | 137 | 100 | 47 | 141 | 160 | 78 | 140 | 152 | 79 | 980 | 513 | 156 |
| chr8:141595218\|141595410 | hsa_circ_0008113 | 24 | 26 | 5 | 18 | 22 | 7 | 8 | 14 | 12 | 94 | 41 | 19 |
| chr8:141582911\|141616013 | #N/A | 6 | 7 | 2 | 6 | 13 | 3 | 12 | 6 | 7 | 93 | 50 | 16 |
| chr8:141582911\|141595410 | hsa_circ_0002965 | 59 | 51 | 19 | 45 | 54 | 23 | 53 | 68 | 38 | 463 | 240 | 96 |
| chr8:141407719\|141415797 | hsa_circ_0004380 | 2 | 12 | 6 | 7 | 5 | 4 | 10 | 4 | 3 | 49 | 30 | 9 |
| chr8:133734286\|133769540 | hsa_circ_0008205 | 12 | 9 | 3 | 8 | 10 | 5 | 8 | 9 | 6 | 98 | 49 | 10 |
| chr8:131302247\|131370389 | #N/A | 129 | 128 | 50 | 94 | 147 | 60 | 82 | 71 | 58 | 831 | 460 | 165 |
| chr8:131249168\|131370389 | hsa_circ_0085614 | 66 | 70 | 23 | 50 | 63 | 19 | 62 | 59 | 30 | 442 | 229 | 74 |
| chr8:131226802\|131370389 | hsa_circ_0009126 | 122 | 132 | 44 | 95 | 102 | 44 | 131 | 132 | 84 | 1140 | 481 | 180 |
| chr8:131220876\|131370389 | #N/A | 9 | 6 | 3 | 12 | 11 | 3 | 3 | 10 | 8 | 43 | 30 | 11 |
| chr8:131199482\|131370389 | #N/A | 10 | 13 | 4 | 8 | 10 | 5 | 6 | 16 | 5 | 94 | 59 | 10 |
| chr8:131191510\|131370389 | hsa_circ_0085606 | 21 | 13 | 6 | 11 | 11 | 6 | 14 | 15 | 9 | 112 | 65 | 14 |
| chr8:131164982\|131370389 | hsa_circ_0085602 | 22 | 29 | 11 | 13 | 8 | 6 | 8 | 13 | 11 | 96 | 40 | 20 |
| chr8:131164982\|131193126 | hsa_circ_0008934 | 154 | 118 | 60 | 80 | 92 | 50 | 118 | 131 | 68 | 997 | 500 | 172 |
| chr8:131164982\|131181313 | hsa_circ_0001824 | 106 | 110 | 48 | 82 | 83 | 54 | 108 | 173 | 89 | 1314 | 652 | 225 |
| chr8:131130407\|131138335 | hsa_circ_0003244 | 25 | 10 | 5 | 5 | 8 | 2 | 12 | 21 | 20 | 179 | 70 | 27 |
| chr8:131092148\|131104389 | hsa_circ_0005718 | 8 | 10 | 4 | 32 | 38 | 21 | 15 | 16 | 6 | 64 | 43 | 11 |
| chr8:124349865\|124351686 | hsa_circ_0085465 | 69 | 65 | 16 | 34 | 45 | 26 | 34 | 29 | 31 | 635 | 196 | 71 |
| chr8:124346118\|124350061 | hsa_circ_0085459 | 21 | 28 | 7 | 14 | 8 | 5 | 3 | 8 | 3 | 93 | 20 | 12 |
| chr8:124346118\|124348772 | hsa_circ_0085458 | 73 | 41 | 31 | 32 | 38 | 18 | 42 | 54 | 31 | 587 | 208 | 74 |
| chr8:124089351\|124117704 | hsa_circ_0085439 | 31 | 9 | 9 | 6 | 13 | 2 | 6 | 13 | 7 | 125 | 42 | 12 |
| chr8:124089351\|124096580 | hsa_circ_0085438 | 31 | 26 | 13 | 15 | 24 | 11 | 25 | 40 | 23 | 373 | 170 | 58 |
| chr8:117668095\|117671219 | hsa_circ_0005231 | 23 | 15 | 4 | 16 | 19 | 8 | 37 | 18 | 9 | 183 | 116 | 27 |
| chr8:109462052\|109468159 | #N/A | 17 | 19 | 5 | 18 | 19 | 12 | 18 | 30 | 14 | 183 | 78 | 27 |
| chr8:109462052\|109462721 | #N/A | 35 | 34 | 19 | 13 | 28 | 20 | 11 | 12 | 10 | 281 | 90 | 35 |
| chr8:107691438\|107696587 | hsa_circ_0008320 | 7 | 8 | 3 | 7 | 9 | 3 | 7 | 11 | 10 | 88 | 51 | 24 |
| chr8:103372299\|103373854 | hsa_circ_0001819 | 55 | 54 | 8 | 32 | 40 | 23 | 54 | 57 | 39 | 499 | 207 | 72 |
| chr8:101718879\|101719225 | hsa_circ_0006623 | 29 | 25 | 9 | 26 | 19 | 14 | 25 | 16 | 14 | 120 | 94 | 22 |
| chr8:100515064\|100523740 | #N/A | 12 | 11 | 6 | 12 | 13 | 8 | 19 | 9 | 7 | 174 | 75 | 24 |
| chr7:99621042\|99621930 | hsa_circ_0001727 | 90 | 58 | 24 | 60 | 60 | 20 | 83 | 115 | 65 | 625 | 380 | 129 |
| chr7:99090663\|99092254 | hsa_circ_0001726 | 22 | 13 | 4 | 5 | 5 | 3 | 3 | 2 | 4 | 75 | 33 | 9 |
| chr7:99001021\|99002576 | #N/A | 23 | 22 | 18 | 24 | 36 | 22 | 19 | 15 | 14 | 129 | 74 | 23 |
| chr7:92921015\|92926555 | hsa_circ_0002620 | 10 | 9 | 7 | 9 | 10 | 7 | 6 | 16 | 10 | 150 | 68 | 15 |
| chr7:91924203\|91957214 | #N/A | 24 | 12 | 14 | 12 | 9 | 12 | 16 | 11 | 5 | 101 | 26 | 16 |
| chr7:91924203\|91936970 | #N/A | 23 | 12 | 12 | 15 | 11 | 10 | 9 | 10 | 4 | 109 | 47 | 26 |
| chr7:8043538\|8047896 | #N/A | 5 | 4 | 2 | 5 | 4 | 6 | 2 | 4 | 4 | 15 | 11 | 2 |
| chr7:7826419\|7841374 | hsa_circ_0001676 | 26 | 33 | 6 | 24 | 32 | 16 | 26 | 31 | 16 | 189 | 109 | 29 |
| chr7:77407655\|77410091 | #N/A | 6 | 5 | 7 | 8 | 11 | 6 | 7 | 12 | 4 | 54 | 43 | 16 |
| chr7:77407655\|77408131 | #N/A | 35 | 26 | 11 | 27 | 27 | 20 | 35 | 34 | 30 | 245 | 162 | 41 |
| chr7:77214860\|77230123 | hsa_circ_0003764 | 19 | 16 | 2 | 18 | 17 | 2 | 21 | 14 | 5 | 116 | 58 | 24 |
| chr7:72301331\|72302378 | hsa_circ_0006688 | 4 | 9 | 3 | 3 | 9 | 3 | 2 | 6 | 4 | 32 | 25 | 12 |
| chr7:72301272\|72302378 | hsa_circ_0005878 | 16 | 6 | 6 | 8 | 12 | 6 | 22 | 20 | 6 | 157 | 66 | 20 |
| chr7:6854395\|6862991 | hsa_circ_0003943 | 10 | 8 | 4 | 8 | 4 | 2 | 10 | 6 | 9 | 63 | 37 | 9 |
| chr7:66458204\|66459328 | hsa_circ_0003203 | 51 | 30 | 23 | 28 | 36 | 7 | 34 | 29 | 25 | 351 | 174 | 54 |
| chr7:66456124\|66459328 | #N/A | 27 | 28 | 6 | 8 | 17 | 7 | 13 | 16 | 5 | 167 | 53 | 33 |
| chr7:65595731\|65599361 | hsa_circ_0080342 | 10 | 16 | 8 | 14 | 9 | 10 | 5 | 2 | 6 | 133 | 52 | 19 |
| chr7:65592691\|65599361 | hsa_circ_0004604 | 48 | 61 | 17 | 32 | 48 | 35 | 40 | 34 | 16 | 330 | 180 | 51 |
| chr7:6085702\|6089676 | hsa_circ_0001673 | 32 | 37 | 5 | 30 | 13 | 13 | 52 | 67 | 23 | 267 | 162 | 83 |
| chr7:6077055\|6078666 | #N/A | 9 | 7 | 2 | 6 | 19 | 5 | 16 | 14 | 9 | 98 | 44 | 8 |
| chr7:5680785\|5692141 | hsa_circ_0079284 | 16 | 11 | 7 | 15 | 17 | 5 | 19 | 18 | 18 | 125 | 68 | 26 |
| chr7:5256194\|5256818 | hsa_circ_0002685 | 11 | 5 | 2 | 9 | 17 | 8 | 9 | 6 | 6 | 62 | 37 | 9 |
| chr7:50358644\|50367353 | hsa_circ_0001708 | 33 | 28 | 6 | 29 | 44 | 23 | 24 | 39 | 17 | 262 | 146 | 38 |
| chr7:48541722\|48542148 | hsa_circ_0001707 | 34 | 41 | 14 | 18 | 33 | 22 | 51 | 46 | 18 | 390 | 122 | 57 |
| chr7:44739740\|44741214 | hsa_circ_0009092 | 10 | 15 | 3 | 21 | 23 | 12 | 9 | 4 | 7 | 35 | 28 | 8 |
| chr7:40037093\|40041630 | hsa_circ_0003951 | 7 | 2 | 4 | 4 | 5 | 3 | 12 | 11 | 4 | 116 | 37 | 11 |
| chr7:36450123\|36450775 | hsa_circ_0079875 | 18 | 14 | 10 | 15 | 17 | 9 | 18 | 14 | 7 | 154 | 62 | 21 |
| chr7:35707044\|35712888 | hsa_circ_0001696 | 14 | 13 | 5 | 15 | 14 | 11 | 25 | 21 | 22 | 156 | 91 | 41 |
| chr7:32672155\|32718742 | #N/A | 14 | 12 | 9 | 7 | 8 | 3 | 10 | 7 | 2 | 121 | 24 | 19 |
| chr7:32672155\|32678977 | hsa_circ_0006010 | 9 | 7 | 4 | 13 | 12 | 4 | 13 | 4 | 3 | 112 | 29 | 16 |
| chr7:27824782\|27825108 | hsa_circ_0079668 | 36 | 29 | 13 | 16 | 17 | 15 | 13 | 14 | 14 | 131 | 61 | 33 |
| chr7:27668990\|27689252 | hsa_circ_0006773 | 2 | 13 | 2 | 7 | 8 | 2 | 8 | 10 | 9 | 76 | 30 | 16 |
| chr7:26724355\|26729981 | hsa_circ_0079658 | 9 | 9 | 2 | 5 | 8 | 3 | 11 | 2 | 2 | 62 | 32 | 10 |
| chr7:24663285\|24708279 | hsa_circ_0001686 | 33 | 30 | 16 | 9 | 7 | 5 | 13 | 10 | 2 | 79 | 18 | 16 |
| chr7:24663285\|24690331 | hsa_circ_0001685 | 21 | 29 | 10 | 11 | 18 | 7 | 12 | 23 | 8 | 239 | 87 | 31 |
| chr7:23650790\|23651172 | hsa_circ_0001684 | 8 | 12 | 7 | 14 | 14 | 11 | 26 | 31 | 18 | 274 | 116 | 42 |
| chr7:23224689\|23224917 | hsa_circ_0005096 | 15 | 14 | 2 | 13 | 18 | 2 | 7 | 4 | 5 | 81 | 35 | 16 |
| chr7:2269619\|2270362 | hsa_circ_0004961 | 21 | 12 | 8 | 13 | 7 | 5 | 16 | 28 | 8 | 135 | 91 | 31 |
| chr7:17908030\|17915413 | hsa_circ_0005519 | 23 | 10 | 5 | 11 | 7 | 5 | 8 | 8 | 9 | 124 | 58 | 16 |
| chr7:17885218\|17890587 | hsa_circ_0005535 | 22 | 25 | 7 | 18 | 30 | 4 | 34 | 29 | 16 | 295 | 96 | 37 |
| chr7:16298015\|16317851 | hsa_circ_0079480 | 163 | 130 | 46 | 119 | 127 | 71 | 96 | 101 | 53 | 1448 | 553 | 248 |
| chr7:158580695\|158591763 | hsa_circ_0001777 | 42 | 38 | 12 | 28 | 30 | 11 | 39 | 39 | 17 | 472 | 125 | 61 |
| chr7:158566032\|158591763 | hsa_circ_0083220 | 5 | 8 | 5 | 6 | 8 | 3 | 8 | 9 | 7 | 101 | 53 | 17 |
| chr7:158552177\|158557544 | hsa_circ_0001776 | 75 | 63 | 19 | 41 | 53 | 22 | 92 | 148 | 78 | 781 | 396 | 172 |
| chr7:156956504\|156963144 | hsa_circ_0083142 | 10 | 11 | 3 | 15 | 12 | 4 | 16 | 10 | 2 | 119 | 41 | 13 |
| chr7:156619299\|156629579 | hsa_circ_0005939 | 25 | 18 | 5 | 19 | 16 | 6 | 26 | 19 | 9 | 155 | 77 | 22 |
| chr7:155499554\|155511137 | hsa_circ_0002094 | 11 | 13 | 3 | 5 | 13 | 6 | 18 | 33 | 13 | 60 | 69 | 28 |
| chr7:155465561\|155473602 | hsa_circ_0001772 | 406 | 328 | 143 | 210 | 250 | 142 | 454 | 756 | 443 | 4569 | 2196 | 944 |
| chr7:155457869\|155473602 | hsa_circ_0001771 | 76 | 69 | 32 | 62 | 42 | 30 | 71 | 88 | 42 | 534 | 303 | 103 |
| chr7:152007051\|152012423 | hsa_circ_0001769 | 24 | 12 | 4 | 12 | 18 | 6 | 23 | 34 | 22 | 139 | 100 | 33 |
| chr7:151902191\|151904513 | hsa_circ_0083014 | 5 | 8 | 9 | 5 | 5 | 4 | 4 | 7 | 3 | 59 | 23 | 6 |
| chr7:151181823\|151195266 | hsa_circ_0004779 | 64 | 40 | 11 | 36 | 35 | 25 | 31 | 38 | 22 | 339 | 176 | 55 |
| chr7:148543589\|148544397 | hsa_circ_0005284 | 11 | 8 | 5 | 8 | 16 | 10 | 4 | 6 | 3 | 53 | 30 | 21 |
| chr7:148543562\|148544397 | hsa_circ_0006357 | 72 | 69 | 17 | 51 | 60 | 47 | 67 | 82 | 65 | 490 | 291 | 110 |
| chr7:148529726\|148544397 | hsa_circ_0008324 | 15 | 7 | 5 | 17 | 8 | 8 | 16 | 31 | 17 | 113 | 61 | 42 |
| chr7:139801751\|139810984 | hsa_circ_0082696 | 15 | 7 | 4 | 11 | 14 | 4 | 11 | 11 | 11 | 107 | 77 | 27 |
| chr7:139415731\|139416814 | hsa_circ_0001756 | 8 | 7 | 4 | 11 | 10 | 3 | 9 | 10 | 5 | 44 | 20 | 8 |
| chr7:138958043\|138958820 | hsa_circ_0082650 | 5 | 7 | 5 | 3 | 4 | 4 | 7 | 3 | 5 | 40 | 26 | 8 |
| chr7:138951079\|138957186 | hsa_circ_0005594 | 6 | 23 | 3 | 8 | 16 | 4 | 12 | 3 | 6 | 123 | 39 | 18 |
| chr7:131071879\|131084192 | hsa_circ_0001747 | 47 | 43 | 23 | 28 | 34 | 10 | 49 | 66 | 33 | 511 | 248 | 122 |
| chr7:131071879\|131073731 | hsa_circ_0002117 | 19 | 9 | 4 | 5 | 5 | 8 | 4 | 3 | 2 | 58 | 24 | 13 |
| chr7:131060183\|131084192 | hsa_circ_0001746 | 66 | 62 | 29 | 43 | 29 | 20 | 45 | 74 | 33 | 724 | 290 | 113 |
| chr7:129760589\|129762042 | hsa_circ_0002190 | 43 | 42 | 8 | 24 | 48 | 12 | 32 | 62 | 39 | 421 | 172 | 74 |
| chr7:128655033\|128658211 | hsa_circ_0001741 | 20 | 26 | 6 | 10 | 11 | 6 | 14 | 24 | 18 | 163 | 101 | 27 |
| chr7:117825701\|117828459 | hsa_circ_0001740 | 22 | 12 | 8 | 13 | 12 | 5 | 12 | 20 | 18 | 279 | 175 | 80 |
| chr7:105738136\|105739728 | hsa_circ_0081873 | 5 | 3 | 2 | 8 | 18 | 4 | 7 | 9 | 7 | 55 | 38 | 9 |
| chr7:104714066\|104717880 | hsa_circ_0001736 | 20 | 17 | 11 | 10 | 11 | 7 | 11 | 25 | 7 | 201 | 77 | 20 |
| chr7:104702611\|104707254 | hsa_circ_0081813 | 8 | 8 | 5 | 13 | 7 | 5 | 12 | 15 | 10 | 91 | 44 | 11 |
| chr7:103801520\|103808973 | hsa_circ_0081797 | 12 | 5 | 6 | 8 | 7 | 6 | 2 | 6 | 4 | 120 | 42 | 14 |
| chr7:102962379\|102963241 | hsa_circ_0081751 | 51 | 56 | 26 | 13 | 20 | 14 | 16 | 20 | 5 | 169 | 53 | 24 |
| chr7:102944289\|102944937 | hsa_circ_0008540 | 7 | 15 | 4 | 11 | 17 | 8 | 5 | 10 | 18 | 98 | 32 | 18 |
| chr7:102039995\|102047945 | hsa_circ_0007004 | 2 | 4 | 2 | 2 | 5 | 5 | 2 | 4 | 6 | 34 | 13 | 3 |
| chr7:101870647\|101870949 | hsa_circ_0006501 | 27 | 47 | 13 | 57 | 81 | 25 | 40 | 61 | 43 | 255 | 216 | 60 |
| chr6:99912480\|99916494 | hsa_circ_0004999 | 9 | 8 | 7 | 7 | 6 | 3 | 7 | 10 | 9 | 180 | 54 | 27 |
| chr6:99860427\|99864304 | hsa_circ_0001630 | 9 | 7 | 4 | 6 | 5 | 3 | 14 | 22 | 10 | 125 | 49 | 16 |
| chr6:90564536\|90566918 | #N/A | 14 | 14 | 5 | 9 | 20 | 7 | 12 | 7 | 5 | 117 | 40 | 16 |
| chr6:90556281\|90566918 | hsa_circ_0001621 | 12 | 15 | 8 | 13 | 8 | 5 | 20 | 22 | 9 | 199 | 76 | 31 |
| chr6:87925621\|87928449 | hsa_circ_0004058 | 13 | 10 | 8 | 12 | 19 | 7 | 19 | 13 | 10 | 130 | 63 | 22 |
| chr6:84894905\|84896341 | hsa_circ_0077223 | 22 | 15 | 11 | 16 | 13 | 3 | 19 | 16 | 7 | 250 | 87 | 26 |
| chr6:80364212\|80383516 | #N/A | 20 | 13 | 7 | 8 | 14 | 11 | 7 | 26 | 12 | 129 | 45 | 33 |
| chr6:79752560\|79770535 | hsa_circ_0001615 | 15 | 19 | 8 | 11 | 11 | 4 | 37 | 18 | 12 | 249 | 121 | 53 |
| chr6:76412361\|76412788 | hsa_circ_0001614 | 13 | 7 | 7 | 3 | 3 | 3 | 9 | 9 | 5 | 116 | 42 | 8 |
| chr6:76357447\|76369123 | hsa_circ_0007002 | 24 | 24 | 8 | 26 | 30 | 16 | 9 | 7 | 4 | 110 | 47 | 28 |
| chr6:76344423\|76373234 | hsa_circ_0077083 | 28 | 15 | 10 | 7 | 5 | 8 | 5 | 7 | 4 | 84 | 33 | 12 |
| chr6:76331248\|76344527 | hsa_circ_0077078 | 24 | 12 | 4 | 8 | 9 | 6 | 12 | 21 | 9 | 148 | 62 | 10 |
| chr6:7602856\|7606428 | hsa_circ_0001574 | 18 | 19 | 2 | 13 | 7 | 8 | 5 | 15 | 10 | 140 | 64 | 12 |
| chr6:57017019\|57025950 | hsa_circ_0002638 | 70 | 64 | 14 | 41 | 43 | 15 | 66 | 95 | 40 | 677 | 325 | 91 |
| chr6:56989532\|57006893 | hsa_circ_0007587 | 22 | 16 | 10 | 7 | 9 | 5 | 9 | 4 | 4 | 108 | 43 | 16 |
| chr6:56989532\|56999668 | hsa_circ_0076881 | 35 | 26 | 18 | 16 | 16 | 12 | 15 | 8 | 7 | 250 | 69 | 35 |
| chr6:56989532\|56993638 | #N/A | 9 | 4 | 8 | 6 | 6 | 3 | 2 | 6 | 4 | 61 | 20 | 13 |
| chr6:56915572\|56920595 | #N/A | 86 | 57 | 25 | 31 | 15 | 19 | 19 | 22 | 10 | 410 | 130 | 40 |
| chr6:52935855\|52941341 | hsa_circ_0003700 | 136 | 88 | 39 | 101 | 117 | 50 | 126 | 189 | 102 | 1103 | 557 | 216 |
| chr6:4891947\|4892613 | hsa_circ_0008285 | 345 | 348 | 148 | 243 | 287 | 166 | 360 | 469 | 305 | 2337 | 1582 | 572 |
| chr6:4891947\|4892337 | #N/A | 8 | 11 | 4 | 4 | 10 | 9 | 20 | 21 | 20 | 96 | 89 | 37 |
| chr6:47575663\|47580272 | #N/A | 29 | 19 | 9 | 15 | 23 | 3 | 15 | 13 | 6 | 255 | 99 | 30 |
| chr6:42630996\|42633983 | hsa_circ_0002906 | 43 | 33 | 8 | 29 | 26 | 14 | 53 | 39 | 26 | 457 | 207 | 60 |
| chr6:42571326\|42580029 | hsa_circ_0002732 | 4 | 5 | 4 | 5 | 2 | 3 | 17 | 11 | 8 | 65 | 32 | 5 |
| chr6:42571326\|42574389 | hsa_circ_0003177 | 16 | 12 | 2 | 10 | 13 | 8 | 18 | 12 | 7 | 170 | 75 | 14 |
| chr6:42559889\|42562042 | hsa_circ_0001603 | 46 | 54 | 14 | 36 | 53 | 30 | 55 | 75 | 51 | 510 | 243 | 88 |
| chr6:42541472\|42562042 | hsa_circ_0076347 | 14 | 3 | 3 | 9 | 11 | 7 | 24 | 22 | 26 | 152 | 94 | 29 |
| chr6:41839302\|41859613 | #N/A | 24 | 18 | 8 | 20 | 27 | 7 | 22 | 28 | 14 | 222 | 85 | 28 |
| chr6:36869997\|36870210 | hsa_circ_0004143 | 7 | 10 | 4 | 14 | 17 | 4 | 3 | 4 | 5 | 46 | 31 | 10 |
| chr6:34574332\|34614575 | hsa_circ_0009095 | 5 | 5 | 3 | 11 | 8 | 5 | 19 | 24 | 11 | 61 | 35 | 21 |
| chr6:34574332\|34574681 | hsa_circ_0006757 | 20 | 17 | 3 | 19 | 7 | 11 | 24 | 32 | 18 | 143 | 110 | 31 |
| chr6:3076998\|3078169 | hsa_circ_0001571 | 9 | 11 | 3 | 6 | 9 | 5 | 16 | 16 | 11 | 44 | 47 | 12 |
| chr6:20781376\|20846409 | hsa_circ_0008086 | 16 | 27 | 5 | 24 | 25 | 21 | 14 | 11 | 11 | 140 | 71 | 24 |
| chr6:18256592\|18258636 | hsa_circ_0008846 | 15 | 12 | 4 | 8 | 19 | 10 | 12 | 13 | 5 | 85 | 35 | 15 |
| chr6:18236683\|18237747 | #N/A | 86 | 63 | 18 | 24 | 26 | 29 | 20 | 16 | 18 | 458 | 103 | 48 |
| chr6:17669524\|17675264 | hsa_circ_0075737 | 42 | 17 | 7 | 19 | 16 | 13 | 23 | 16 | 12 | 265 | 124 | 30 |
| chr6:17669206\|17669777 | hsa_circ_0002049 | 18 | 22 | 6 | 18 | 25 | 18 | 8 | 7 | 6 | 99 | 67 | 18 |
| chr6:17665470\|17669777 | hsa_circ_0007268 | 56 | 45 | 20 | 27 | 39 | 13 | 56 | 75 | 32 | 543 | 257 | 67 |
| chr6:17665470\|17669259 | hsa_circ_0002492 | 23 | 19 | 9 | 14 | 24 | 16 | 3 | 5 | 6 | 95 | 61 | 22 |
| chr6:17646298\|17649531 | hsa_circ_0007370 | 17 | 12 | 4 | 19 | 20 | 14 | 35 | 18 | 14 | 210 | 80 | 23 |
| chr6:170852689\|170858201 | hsa_circ_0006107 | 10 | 8 | 2 | 9 | 10 | 5 | 16 | 17 | 11 | 109 | 63 | 16 |
| chr6:167435897\|167447459 | hsa_circ_0004558 | 12 | 14 | 11 | 13 | 16 | 7 | 13 | 29 | 13 | 171 | 86 | 27 |
| chr6:163876311\|163899928 | hsa_circ_0005328 | 14 | 10 | 5 | 13 | 5 | 4 | 16 | 18 | 10 | 113 | 60 | 16 |
| chr6:160467530\|160469575 | #N/A | 10 | 8 | 3 | 4 | 11 | 4 | 7 | 25 | 14 | 131 | 73 | 30 |
| chr6:159001972\|159010814 | hsa_circ_0001661 | 12 | 3 | 4 | 7 | 4 | 5 | 9 | 12 | 13 | 133 | 82 | 33 |
| chr6:158994452\|159010814 | hsa_circ_0004587 | 16 | 21 | 3 | 14 | 13 | 6 | 12 | 31 | 16 | 151 | 87 | 35 |
| chr6:155108995\|155114110 | hsa_circ_0078373 | 11 | 12 | 6 | 12 | 18 | 9 | 13 | 7 | 8 | 105 | 47 | 18 |
| chr6:155095123\|155116273 | hsa_circ_0001654 | 15 | 12 | 5 | 9 | 14 | 8 | 11 | 21 | 4 | 181 | 74 | 23 |
| chr6:150092298\|150094305 | hsa_circ_0006936 | 37 | 52 | 17 | 40 | 61 | 33 | 16 | 23 | 12 | 565 | 280 | 46 |
| chr6:149924404\|149925918 | hsa_circ_0001651 | 24 | 19 | 6 | 6 | 19 | 5 | 7 | 3 | 4 | 140 | 46 | 17 |
| chr6:146209156\|146216113 | hsa_circ_0001649 | 20 | 21 | 6 | 3 | 12 | 11 | 17 | 17 | 5 | 193 | 49 | 26 |
| chr6:144858718\|144860579 | hsa_circ_0001647 | 2 | 9 | 6 | 4 | 5 | 8 | 3 | 5 | 2 | 63 | 26 | 13 |
| chr6:139264650\|139265759 | hsa_circ_0004368 | 40 | 41 | 13 | 34 | 22 | 9 | 39 | 53 | 23 | 391 | 181 | 76 |
| chr6:137015278\|137019820 | hsa_circ_0006856 | 20 | 12 | 9 | 12 | 14 | 3 | 9 | 16 | 10 | 106 | 59 | 24 |
| chr6:13639795\|13644961 | hsa_circ_0001578 | 136 | 107 | 55 | 59 | 72 | 29 | 97 | 129 | 65 | 896 | 407 | 156 |
| chr6:13632602\|13644961 | hsa_circ_0001577 | 114 | 99 | 30 | 33 | 31 | 18 | 27 | 36 | 22 | 478 | 251 | 80 |
| chr6:13579683\|13584457 | hsa_circ_0007218 | 73 | 55 | 27 | 60 | 53 | 37 | 71 | 67 | 27 | 613 | 161 | 70 |
| chr6:135360711\|135363264 | hsa_circ_0001641 | 6 | 6 | 5 | 3 | 3 | 3 | 3 | 13 | 5 | 67 | 33 | 15 |
| chr6:131520601\|131540948 | #N/A | 16 | 21 | 6 | 9 | 15 | 4 | 8 | 8 | 6 | 102 | 47 | 22 |
| chr6:131481199\|131490413 | #N/A | 36 | 26 | 6 | 13 | 18 | 11 | 22 | 25 | 11 | 246 | 91 | 42 |
| chr6:131466425\|131490413 | #N/A | 80 | 77 | 28 | 33 | 32 | 24 | 36 | 34 | 22 | 572 | 152 | 75 |
| chr6:130505248\|130505768 | hsa_circ_0008833 | 13 | 8 | 5 | 9 | 14 | 6 | 4 | 14 | 12 | 97 | 60 | 25 |
| chr6:126176166\|126176386 | #N/A | 20 | 9 | 2 | 6 | 8 | 3 | 5 | 2 | 3 | 36 | 33 | 7 |
| chr6:117019867\|117026323 | hsa_circ_0001639 | 10 | 10 | 2 | 6 | 9 | 4 | 8 | 4 | 7 | 109 | 26 | 11 |
| chr6:117010483\|117013555 | #N/A | 70 | 60 | 18 | 16 | 11 | 22 | 9 | 6 | 3 | 274 | 35 | 14 |
| chr6:111583460\|111585149 | hsa_circ_0001638 | 10 | 11 | 9 | 10 | 18 | 13 | 12 | 28 | 8 | 126 | 40 | 28 |
| chr6:109466422\|109468140 | hsa_circ_0004131 | 62 | 31 | 7 | 29 | 24 | 11 | 34 | 34 | 12 | 328 | 162 | 46 |
| chr6:108243001\|108250718 | hsa_circ_0001635 | 14 | 9 | 6 | 6 | 11 | 9 | 10 | 18 | 7 | 176 | 55 | 16 |
| chr6:10703638\|10705077 | hsa_circ_0008053 | 24 | 14 | 8 | 3 | 12 | 3 | 11 | 13 | 6 | 140 | 45 | 19 |
| chr5:95242227\|95268417 | hsa_circ_0009176 | 5 | 6 | 3 | 3 | 7 | 2 | 6 | 7 | 5 | 50 | 23 | 10 |
| chr5:95091100\|95099324 | hsa_circ_0007444 | 179 | 138 | 69 | 74 | 90 | 60 | 120 | 151 | 100 | 1623 | 572 | 291 |
| chr5:93964516\|93966448 | hsa_circ_0073371 | 15 | 14 | 7 | 6 | 3 | 3 | 11 | 15 | 10 | 135 | 43 | 22 |
| chr5:78952781\|78964851 | hsa_circ_0008517 | 14 | 7 | 7 | 27 | 30 | 18 | 14 | 2 | 4 | 96 | 32 | 8 |
| chr5:78915435\|78919312 | hsa_circ_0001504 | 37 | 29 | 8 | 8 | 27 | 12 | 16 | 14 | 4 | 194 | 96 | 31 |
| chr5:78914470\|78915906 | hsa_circ_0001503 | 30 | 22 | 10 | 25 | 23 | 7 | 22 | 29 | 15 | 317 | 136 | 49 |
| chr5:76342172\|76344812 | #N/A | 19 | 12 | 10 | 15 | 12 | 3 | 28 | 27 | 8 | 211 | 72 | 35 |
| chr5:76342172\|76344097 | hsa_circ_0008620 | 76 | 67 | 19 | 48 | 44 | 22 | 70 | 99 | 40 | 766 | 360 | 120 |
| chr5:75993812\|75997038 | #N/A | 24 | 8 | 5 | 8 | 5 | 8 | 11 | 13 | 2 | 103 | 43 | 8 |
| chr5:74041503\|74046492 | #N/A | 17 | 19 | 3 | 9 | 8 | 6 | 15 | 22 | 7 | 119 | 63 | 29 |
| chr5:74011335\|74012498 | hsa_circ_0007559 | 2 | 3 | 2 | 4 | 4 | 3 | 7 | 5 | 2 | 49 | 16 | 8 |
| chr5:72374007\|72378652 | hsa_circ_0072979 | 17 | 11 | 7 | 7 | 18 | 9 | 14 | 27 | 14 | 228 | 81 | 38 |
| chr5:72370569\|72373320 | hsa_circ_0002490 | 205 | 220 | 68 | 156 | 204 | 113 | 112 | 156 | 107 | 1832 | 826 | 299 |
| chr5:72354260\|72373320 | hsa_circ_0003571 | 131 | 108 | 23 | 129 | 141 | 35 | 122 | 89 | 58 | 780 | 470 | 128 |
| chr5:72285254\|72286691 | hsa_circ_0072954 | 196 | 174 | 61 | 64 | 71 | 46 | 46 | 58 | 32 | 959 | 283 | 153 |
| chr5:72157635\|72161556 | hsa_circ_0002692 | 17 | 20 | 3 | 30 | 21 | 13 | 27 | 27 | 13 | 195 | 73 | 48 |
| chr5:68487622\|68492936 | #N/A | 23 | 18 | 10 | 15 | 22 | 21 | 19 | 23 | 8 | 149 | 61 | 32 |
| chr5:68470704\|68471364 | hsa_circ_0001495 | 58 | 48 | 25 | 53 | 50 | 29 | 83 | 92 | 60 | 704 | 329 | 129 |
| chr5:65284463\|65310553 | hsa_circ_0001493 | 10 | 12 | 4 | 9 | 6 | 5 | 6 | 14 | 3 | 107 | 49 | 18 |
| chr5:65284463\|65290692 | hsa_circ_0001492 | 91 | 75 | 26 | 77 | 81 | 42 | 99 | 163 | 69 | 1167 | 414 | 177 |
| chr5:64824279\|64847463 | #N/A | 15 | 10 | 4 | 14 | 5 | 3 | 7 | 5 | 2 | 155 | 36 | 21 |
| chr5:64824279\|64838675 | #N/A | 33 | 28 | 25 | 13 | 21 | 13 | 9 | 16 | 13 | 246 | 90 | 39 |
| chr5:64824279\|64825026 | #N/A | 73 | 45 | 16 | 24 | 42 | 29 | 29 | 19 | 14 | 418 | 137 | 45 |
| chr5:56542127\|56546968 | hsa_circ_0072547 | 59 | 58 | 21 | 25 | 55 | 21 | 36 | 34 | 28 | 395 | 152 | 40 |
| chr5:56542127\|56545403 | hsa_circ_0002775 | 26 | 10 | 7 | 17 | 23 | 12 | 21 | 15 | 11 | 189 | 78 | 46 |
| chr5:56542127\|56543042 | hsa_circ_0072546 | 26 | 37 | 12 | 17 | 31 | 12 | 14 | 17 | 17 | 246 | 88 | 27 |
| chr5:56526673\|56527148 | hsa_circ_0001486 | 17 | 20 | 4 | 15 | 27 | 10 | 26 | 37 | 21 | 211 | 101 | 44 |
| chr5:56160561\|56161804 | hsa_circ_0001485 | 56 | 44 | 26 | 32 | 36 | 17 | 60 | 79 | 44 | 563 | 296 | 93 |
| chr5:52899282\|52900725 | hsa_circ_0005593 | 4 | 16 | 7 | 16 | 12 | 8 | 2 | 7 | 2 | 99 | 57 | 20 |
| chr5:49694941\|49707217 | hsa_circ_0001481 | 37 | 43 | 13 | 13 | 17 | 5 | 14 | 17 | 10 | 217 | 66 | 40 |
| chr5:43675613\|43677908 | hsa_circ_0003832 | 18 | 10 | 2 | 10 | 21 | 6 | 3 | 3 | 4 | 110 | 46 | 18 |
| chr5:38991051\|39003724 | hsa_circ_0006157 | 3 | 4 | 5 | 3 | 3 | 8 | 19 | 11 | 7 | 107 | 56 | 14 |
| chr5:31421379\|31424578 | hsa_circ_0005524 | 30 | 19 | 11 | 33 | 34 | 10 | 21 | 29 | 17 | 225 | 115 | 36 |
| chr5:179976931\|179980471 | hsa_circ_0008836 | 32 | 36 | 11 | 36 | 49 | 25 | 34 | 24 | 18 | 250 | 157 | 43 |
| chr5:179688684\|179707608 | hsa_circ_0001566 | 8 | 8 | 5 | 3 | 4 | 4 | 4 | 2 | 3 | 51 | 25 | 12 |
| chr5:179665332\|179668155 | hsa_circ_0075341 | 45 | 39 | 12 | 30 | 34 | 20 | 16 | 17 | 23 | 270 | 115 | 37 |
| chr5:179136874\|179147561 | hsa_circ_0002051 | 10 | 9 | 7 | 3 | 6 | 6 | 3 | 6 | 3 | 49 | 32 | 3 |
| chr5:179136874\|179143295 | hsa_circ_0001565 | 13 | 14 | 7 | 11 | 12 | 10 | 8 | 12 | 6 | 98 | 47 | 12 |
| chr5:179018626\|179020646 | #N/A | 9 | 6 | 3 | 8 | 8 | 2 | 5 | 5 | 8 | 55 | 33 | 15 |
| chr5:176618885\|176631293 | hsa_circ_0075157 | 8 | 7 | 5 | 8 | 5 | 4 | 22 | 18 | 13 | 124 | 77 | 18 |
| chr5:176402397\|176409624 | hsa_circ_0008631 | 34 | 41 | 14 | 25 | 33 | 20 | 37 | 40 | 26 | 324 | 177 | 55 |
| chr5:176370336\|176385155 | hsa_circ_0001558 | 82 | 92 | 25 | 62 | 99 | 31 | 127 | 180 | 118 | 1011 | 540 | 172 |
| chr5:171482592\|171484477 | hsa_circ_0001555 | 14 | 6 | 2 | 12 | 6 | 6 | 10 | 13 | 7 | 42 | 35 | 11 |
| chr5:170610199\|170632616 | hsa_circ_0003718 | 13 | 3 | 2 | 8 | 11 | 8 | 26 | 12 | 7 | 100 | 63 | 10 |
| chr5:167995651\|167996003 | hsa_circ_0006894 | 19 | 15 | 2 | 12 | 17 | 8 | 16 | 7 | 14 | 168 | 79 | 25 |
| chr5:167915607\|167921655 | hsa_circ_0001550 | 73 | 46 | 31 | 26 | 36 | 19 | 59 | 62 | 28 | 575 | 267 | 78 |
| chr5:162909648\|162911251 | #N/A | 19 | 17 | 13 | 3 | 5 | 3 | 7 | 5 | 5 | 134 | 39 | 12 |
| chr5:153413351\|153414527 | hsa_circ_0001546 | 53 | 52 | 22 | 49 | 47 | 21 | 75 | 108 | 60 | 601 | 359 | 93 |
| chr5:153409049\|153414527 | hsa_circ_0007773 | 7 | 6 | 2 | 7 | 5 | 3 | 2 | 6 | 3 | 17 | 22 | 3 |
| chr5:150838349\|150859050 | hsa_circ_0074623 | 14 | 20 | 3 | 14 | 17 | 5 | 10 | 8 | 7 | 53 | 26 | 7 |
| chr5:145634506\|145638156 | hsa_circ_0006087 | 10 | 10 | 2 | 7 | 5 | 3 | 12 | 9 | 2 | 58 | 25 | 13 |
| chr5:145197457\|145205763 | hsa_circ_0006528 | 7 | 10 | 6 | 6 | 6 | 3 | 14 | 18 | 5 | 67 | 36 | 20 |
| chr5:142434004\|142437312 | hsa_circ_0074371 | 14 | 20 | 9 | 20 | 23 | 20 | 16 | 15 | 3 | 229 | 92 | 49 |
| chr5:142416761\|142437312 | hsa_circ_0074368 | 8 | 5 | 5 | 6 | 5 | 2 | 12 | 20 | 6 | 94 | 37 | 21 |
| chr5:140961879\|140963182 | hsa_circ_0008982 | 10 | 10 | 3 | 8 | 11 | 6 | 10 | 14 | 14 | 111 | 50 | 13 |
| chr5:138994171\|138994551 | hsa_circ_0006716 | 100 | 98 | 23 | 81 | 90 | 53 | 28 | 24 | 22 | 587 | 311 | 72 |
| chr5:138979957\|138994551 | hsa_circ_0005728 | 141 | 138 | 31 | 91 | 97 | 55 | 123 | 86 | 57 | 1168 | 451 | 134 |
| chr5:138699448\|138700432 | hsa_circ_0001538 | 150 | 136 | 46 | 75 | 83 | 45 | 55 | 72 | 42 | 965 | 328 | 124 |
| chr5:138614016\|138614818 | hsa_circ_0008922 | 22 | 24 | 6 | 17 | 18 | 11 | 12 | 12 | 7 | 218 | 149 | 6 |
| chr5:137320946\|137324004 | hsa_circ_0001535 | 584 | 504 | 163 | 371 | 424 | 222 | 448 | 427 | 253 | 4995 | 1920 | 587 |
| chr5:137288317\|137290065 | hsa_circ_0008177 | 16 | 22 | 7 | 13 | 19 | 12 | 18 | 33 | 20 | 227 | 119 | 27 |
| chr5:134076753\|134079742 | hsa_circ_0003154 | 8 | 11 | 10 | 10 | 8 | 7 | 10 | 18 | 13 | 120 | 77 | 21 |
| chr5:132426885\|132428481 | hsa_circ_0001531 | 2 | 7 | 3 | 2 | 10 | 4 | 3 | 6 | 7 | 40 | 28 | 14 |
| chr5:132227856\|132228810 | hsa_circ_0001529 | 16 | 15 | 7 | 12 | 22 | 7 | 28 | 42 | 21 | 196 | 97 | 41 |
| chr5:131013396\|131044965 | hsa_circ_0001524 | 28 | 27 | 7 | 12 | 12 | 6 | 29 | 37 | 15 | 206 | 120 | 33 |
| chr5:122881111\|122893258 | hsa_circ_0001522 | 126 | 125 | 56 | 54 | 35 | 30 | 55 | 44 | 30 | 808 | 298 | 99 |
| chr5:114875362\|114878598 | #N/A | 6 | 8 | 4 | 10 | 15 | 5 | 5 | 28 | 11 | 100 | 49 | 16 |
| chr5:112336781\|112337371 | hsa_circ_0002462 | 13 | 6 | 5 | 4 | 7 | 4 | 18 | 20 | 5 | 110 | 66 | 12 |
| chr5:112321532\|112339774 | hsa_circ_0001520 | 24 | 23 | 11 | 11 | 14 | 12 | 21 | 15 | 17 | 186 | 102 | 29 |
| chr5:112321532\|112337371 | hsa_circ_0073608 | 7 | 8 | 5 | 2 | 3 | 4 | 8 | 7 | 3 | 68 | 47 | 15 |
| chr5:111611023\|111643187 | #N/A | 66 | 40 | 14 | 27 | 31 | 17 | 19 | 18 | 27 | 196 | 107 | 37 |
| chr5:109049221\|109065214 | hsa_circ_0001519 | 22 | 23 | 16 | 11 | 20 | 6 | 34 | 25 | 26 | 331 | 134 | 60 |
| chr5:109049221\|109051965 | hsa_circ_0008058 | 17 | 18 | 2 | 10 | 16 | 14 | 22 | 27 | 11 | 152 | 75 | 42 |
| chr4:99495608\|99496056 | #N/A | 90 | 71 | 21 | 74 | 83 | 34 | 101 | 97 | 72 | 681 | 406 | 155 |
| chr4:90743397\|90756843 | #N/A | 8 | 7 | 6 | 11 | 14 | 6 | 15 | 15 | 11 | 80 | 52 | 18 |
| chr4:89859239\|89873545 | #N/A | 5 | 12 | 3 | 5 | 3 | 3 | 12 | 4 | 6 | 64 | 16 | 9 |
| chr4:89827530\|89912301 | #N/A | 9 | 3 | 3 | 6 | 7 | 3 | 7 | 5 | 4 | 35 | 25 | 7 |
| chr4:89827530\|89870589 | #N/A | 8 | 16 | 4 | 15 | 9 | 6 | 13 | 18 | 6 | 170 | 63 | 15 |
| chr4:89827530\|89859392 | hsa_circ_0070434 | 22 | 37 | 6 | 14 | 22 | 20 | 14 | 15 | 8 | 171 | 96 | 19 |
| chr4:89028336\|89036210 | #N/A | 8 | 8 | 6 | 6 | 3 | 6 | 12 | 8 | 6 | 111 | 40 | 17 |
| chr4:88116476\|88116842 | hsa_circ_0002538 | 108 | 93 | 37 | 47 | 78 | 21 | 108 | 148 | 82 | 1061 | 491 | 173 |
| chr4:88005272\|88016121 | #N/A | 23 | 34 | 8 | 18 | 23 | 18 | 6 | 20 | 18 | 90 | 56 | 23 |
| chr4:88005272\|88012981 | hsa_circ_0070382 | 46 | 45 | 15 | 32 | 49 | 25 | 55 | 55 | 35 | 454 | 180 | 46 |
| chr4:87967318\|87968746 | hsa_circ_0001423 | 124 | 100 | 37 | 93 | 117 | 80 | 95 | 94 | 61 | 543 | 410 | 94 |
| chr4:83891480\|83900159 | hsa_circ_0003148 | 47 | 39 | 11 | 37 | 50 | 20 | 42 | 31 | 36 | 358 | 174 | 42 |
| chr4:83799883\|83803093 | hsa_circ_0006618 | 15 | 15 | 11 | 8 | 21 | 3 | 22 | 30 | 16 | 173 | 82 | 20 |
| chr4:83375875\|83378191 | hsa_circ_0003451 | 16 | 15 | 2 | 2 | 9 | 7 | 8 | 5 | 5 | 64 | 35 | 14 |
| chr4:79747191\|79772210 | hsa_circ_0070190 | 13 | 15 | 8 | 2 | 10 | 5 | 12 | 6 | 2 | 139 | 46 | 15 |
| chr4:78694235\|78697546 | hsa_circ_0006168 | 53 | 43 | 21 | 34 | 48 | 36 | 67 | 95 | 50 | 654 | 288 | 99 |
| chr4:77065302\|77065626 | hsa_circ_0070040 | 77 | 83 | 22 | 55 | 48 | 37 | 63 | 68 | 43 | 511 | 262 | 79 |
| chr4:77055328\|77065626 | hsa_circ_0070039 | 22 | 26 | 13 | 13 | 9 | 6 | 11 | 15 | 19 | 179 | 79 | 18 |
| chr4:73956384\|73958017 | hsa_circ_0007883 | 23 | 21 | 6 | 10 | 15 | 11 | 9 | 5 | 3 | 53 | 22 | 7 |
| chr4:71808540\|71835741 | #N/A | 30 | 22 | 9 | 20 | 17 | 4 | 30 | 34 | 13 | 266 | 105 | 42 |
| chr4:71808540\|71824757 | #N/A | 13 | 13 | 4 | 5 | 5 | 4 | 7 | 10 | 2 | 62 | 38 | 17 |
| chr4:6995911\|7002978 | hsa_circ_0006681 | 33 | 28 | 16 | 16 | 12 | 14 | 31 | 67 | 36 | 332 | 196 | 64 |
| chr4:57462666\|57470388 | #N/A | 5 | 3 | 3 | 7 | 5 | 2 | 7 | 9 | 5 | 55 | 34 | 5 |
| chr4:56883814\|56885721 | hsa_circ_0069819 | 26 | 19 | 2 | 7 | 10 | 4 | 24 | 31 | 13 | 228 | 93 | 38 |
| chr4:56877578\|56878151 | hsa_circ_0003985 | 28 | 22 | 13 | 17 | 20 | 6 | 39 | 17 | 12 | 257 | 108 | 32 |
| chr4:56277781\|56284152 | hsa_circ_0001414 | 30 | 34 | 16 | 11 | 8 | 7 | 9 | 19 | 4 | 198 | 46 | 21 |
| chr4:54292039\|54310270 | hsa_circ_0001413 | 44 | 52 | 18 | 87 | 123 | 37 | 55 | 72 | 36 | 470 | 245 | 64 |
| chr4:54292039\|54294350 | hsa_circ_0069748 | 54 | 56 | 15 | 49 | 78 | 38 | 40 | 32 | 41 | 293 | 197 | 75 |
| chr4:54280782\|54310270 | hsa_circ_0001412 | 16 | 11 | 8 | 20 | 9 | 6 | 15 | 16 | 10 | 100 | 34 | 16 |
| chr4:54280782\|54294350 | hsa_circ_0007476 | 50 | 52 | 23 | 65 | 70 | 29 | 76 | 77 | 56 | 575 | 291 | 106 |
| chr4:54265897\|54294350 | hsa_circ_0005912 | 14 | 27 | 5 | 14 | 26 | 4 | 19 | 27 | 12 | 103 | 69 | 24 |
| chr4:54249940\|54256040 | hsa_circ_0001411 | 30 | 24 | 16 | 26 | 38 | 22 | 19 | 9 | 7 | 324 | 150 | 41 |
| chr4:52729603\|52758017 | hsa_circ_0007646 | 23 | 27 | 17 | 24 | 29 | 13 | 18 | 22 | 11 | 286 | 106 | 40 |
| chr4:52729603\|52752804 | hsa_circ_0007928 | 9 | 14 | 8 | 5 | 6 | 3 | 5 | 17 | 6 | 124 | 30 | 11 |
| chr4:52729603\|52744020 | hsa_circ_0001410 | 56 | 55 | 14 | 31 | 44 | 24 | 44 | 47 | 38 | 559 | 258 | 80 |
| chr4:48686690\|48712715 | hsa_circ_0002021 | 17 | 11 | 6 | 8 | 10 | 6 | 14 | 9 | 8 | 100 | 50 | 13 |
| chr4:48608455\|48611084 | #N/A | 10 | 11 | 4 | 4 | 7 | 6 | 9 | 3 | 4 | 115 | 25 | 16 |
| chr4:48371866\|48385801 | #N/A | 59 | 38 | 13 | 21 | 18 | 16 | 3 | 14 | 4 | 138 | 52 | 17 |
| chr4:42024855\|42025401 | hsa_circ_0001406 | 12 | 18 | 2 | 7 | 19 | 3 | 7 | 4 | 3 | 109 | 42 | 11 |
| chr4:39915231\|39927553 | hsa_circ_0007308 | 46 | 58 | 21 | 19 | 22 | 10 | 30 | 38 | 25 | 428 | 153 | 71 |
| chr4:39843568\|39846421 | hsa_circ_0006865 | 6 | 2 | 3 | 6 | 6 | 4 | 14 | 8 | 6 | 42 | 28 | 3 |
| chr4:39839476\|39843676 | #N/A | 7 | 17 | 4 | 11 | 4 | 4 | 10 | 20 | 11 | 112 | 58 | 24 |
| chr4:39739040\|39776553 | hsa_circ_0002590 | 25 | 34 | 9 | 9 | 13 | 7 | 16 | 13 | 2 | 185 | 65 | 15 |
| chr4:39739040\|39757359 | hsa_circ_0009154 | 78 | 72 | 31 | 86 | 86 | 45 | 47 | 44 | 39 | 601 | 242 | 75 |
| chr4:39739040\|39747430 | #N/A | 22 | 15 | 18 | 11 | 17 | 18 | 6 | 4 | 3 | 219 | 78 | 12 |
| chr4:39734979\|39747430 | #N/A | 16 | 21 | 3 | 15 | 13 | 24 | 15 | 19 | 11 | 153 | 58 | 13 |
| chr4:39328183\|39329376 | hsa_circ_0001403 | 59 | 51 | 16 | 27 | 25 | 16 | 26 | 28 | 30 | 367 | 132 | 52 |
| chr4:38091553\|38104778 | hsa_circ_0001402 | 18 | 7 | 10 | 16 | 15 | 2 | 14 | 24 | 8 | 167 | 70 | 43 |
| chr4:37633007\|37640126 | hsa_circ_0001400 | 145 | 175 | 52 | 168 | 186 | 69 | 219 | 420 | 245 | 1374 | 893 | 351 |
| chr4:178274462\|178281831 | hsa_circ_0001460 | 191 | 175 | 65 | 49 | 72 | 58 | 34 | 21 | 11 | 729 | 195 | 79 |
| chr4:178274462\|178274882 | hsa_circ_0001459 | 265 | 241 | 84 | 113 | 101 | 61 | 80 | 76 | 43 | 1638 | 532 | 127 |
| chr4:17816476\|17816981 | hsa_circ_0001395 | 267 | 267 | 81 | 180 | 255 | 168 | 158 | 133 | 109 | 1480 | 852 | 262 |
| chr4:170501993\|170511960 | hsa_circ_0071434 | 9 | 9 | 5 | 6 | 6 | 6 | 18 | 18 | 8 | 120 | 34 | 18 |
| chr4:170428188\|170459062 | #N/A | 31 | 14 | 8 | 15 | 22 | 12 | 37 | 26 | 19 | 215 | 99 | 33 |
| chr4:170428188\|170429482 | hsa_circ_0001456 | 13 | 19 | 5 | 12 | 7 | 9 | 8 | 14 | 4 | 99 | 34 | 20 |
| chr4:1696501\|1698078 | hsa_circ_0068850 | 6 | 12 | 5 | 7 | 6 | 9 | 7 | 8 | 5 | 42 | 26 | 9 |
| chr4:160225494\|160235920 | #N/A | 9 | 5 | 4 | 3 | 14 | 3 | 8 | 10 | 5 | 79 | 65 | 18 |
| chr4:160107794\|160164943 | #N/A | 18 | 6 | 3 | 17 | 20 | 5 | 26 | 34 | 20 | 189 | 102 | 50 |
| chr4:154547299\|154553990 | hsa_circ_0071311 | 14 | 7 | 2 | 15 | 12 | 7 | 12 | 11 | 10 | 119 | 46 | 21 |
| chr4:154315414\|154318485 | hsa_circ_0006225 | 34 | 25 | 22 | 26 | 26 | 18 | 25 | 32 | 18 | 150 | 96 | 36 |
| chr4:153332455\|153391140 | #N/A | 17 | 20 | 5 | 6 | 2 | 4 | 7 | 9 | 2 | 80 | 27 | 6 |
| chr4:153332455\|153333681 | hsa_circ_0001451 | 428 | 323 | 114 | 174 | 173 | 80 | 182 | 280 | 192 | 2238 | 1141 | 408 |
| chr4:151719233\|151738409 | hsa_circ_0008618 | 32 | 34 | 8 | 27 | 31 | 14 | 40 | 41 | 27 | 299 | 176 | 60 |
| chr4:151682935\|151729550 | hsa_circ_0071185 | 5 | 6 | 3 | 5 | 15 | 7 | 15 | 12 | 8 | 94 | 44 | 12 |
| chr4:147227078\|147230127 | #N/A | 18 | 24 | 11 | 11 | 19 | 5 | 10 | 8 | 4 | 102 | 42 | 17 |
| chr4:144920564\|144929602 | #N/A | 34 | 39 | 16 | 38 | 22 | 21 | 7 | 9 | 7 | 150 | 137 | 34 |
| chr4:144915311\|144922436 | #N/A | 12 | 8 | 2 | 7 | 3 | 6 | 7 | 10 | 4 | 88 | 32 | 3 |
| chr4:144801564\|145040934 | #N/A | 42 | 40 | 13 | 8 | 13 | 6 | 7 | 15 | 2 | 127 | 48 | 19 |
| chr4:144464662\|144465125 | hsa_circ_0001445 | 467 | 405 | 141 | 250 | 299 | 197 | 235 | 370 | 215 | 2753 | 1028 | 538 |
| chr4:144449021\|144451679 | #N/A | 13 | 6 | 4 | 3 | 10 | 3 | 17 | 15 | 3 | 140 | 37 | 10 |
| chr4:144380538\|144387378 | #N/A | 2 | 9 | 3 | 11 | 7 | 6 | 8 | 19 | 7 | 78 | 32 | 12 |
| chr4:144336630\|144336924 | #N/A | 7 | 17 | 6 | 12 | 12 | 8 | 12 | 19 | 15 | 141 | 58 | 20 |
| chr4:140058784\|140060651 | hsa_circ_0007466 | 50 | 27 | 9 | 34 | 25 | 13 | 45 | 47 | 29 | 380 | 170 | 62 |
| chr4:140046318\|140060651 | hsa_circ_0001441 | 35 | 23 | 12 | 22 | 14 | 10 | 20 | 66 | 26 | 449 | 169 | 90 |
| chr4:140037803\|140060651 | hsa_circ_0007710 | 10 | 13 | 4 | 9 | 7 | 4 | 4 | 16 | 9 | 106 | 51 | 28 |
| chr4:129960196\|130003528 | #N/A | 26 | 17 | 4 | 16 | 9 | 14 | 10 | 9 | 13 | 104 | 36 | 21 |
| chr4:129913322\|129925031 | hsa_circ_0001439 | 299 | 228 | 103 | 189 | 184 | 118 | 280 | 415 | 234 | 3110 | 1266 | 509 |
| chr4:128995615\|129012667 | hsa_circ_0070934 | 25 | 5 | 7 | 5 | 2 | 5 | 3 | 8 | 3 | 72 | 26 | 8 |
| chr4:128995615\|129003460 | hsa_circ_0007619 | 73 | 46 | 18 | 20 | 31 | 15 | 33 | 42 | 18 | 351 | 146 | 51 |
| chr4:128995615\|128999117 | hsa_circ_0001438 | 52 | 54 | 16 | 25 | 22 | 13 | 33 | 34 | 18 | 381 | 149 | 47 |
| chr4:123977542\|123978443 | hsa_circ_0006112 | 8 | 10 | 3 | 11 | 10 | 6 | 9 | 4 | 3 | 157 | 68 | 18 |
| chr4:122725777\|122731254 | hsa_circ_0008244 | 10 | 9 | 2 | 6 | 9 | 3 | 15 | 19 | 15 | 97 | 57 | 24 |
| chr4:1219148\|1235307 | hsa_circ_0001386 | 14 | 11 | 3 | 8 | 19 | 4 | 12 | 26 | 12 | 97 | 68 | 35 |
| chr4:110580167\|110585550 | hsa_circ_0070660 | 13 | 14 | 3 | 5 | 9 | 4 | 6 | 6 | 5 | 49 | 38 | 15 |
| chr4:110412483\|110416012 | hsa_circ_0001436 | 56 | 43 | 22 | 86 | 110 | 50 | 105 | 108 | 52 | 451 | 273 | 77 |
| chr4:110384057\|110384800 | hsa_circ_0003875 | 13 | 3 | 2 | 7 | 8 | 5 | 6 | 6 | 6 | 52 | 22 | 8 |
| chr4:107216251\|107230146 | hsa_circ_0003673 | 11 | 17 | 5 | 4 | 11 | 10 | 22 | 9 | 8 | 81 | 43 | 12 |
| chr4:106345353\|106377902 | hsa_circ_0001434 | 12 | 13 | 6 | 5 | 9 | 5 | 6 | 22 | 9 | 120 | 31 | 25 |
| chr4:105439734\|105440611 | #N/A | 57 | 68 | 18 | 30 | 45 | 21 | 35 | 26 | 16 | 396 | 191 | 54 |
| chr4:103644028\|103647840 | hsa_circ_0006007 | 13 | 13 | 4 | 9 | 7 | 3 | 7 | 17 | 14 | 131 | 54 | 17 |
| chr4:103635595\|103647840 | hsa_circ_0001432 | 23 | 14 | 6 | 13 | 14 | 4 | 29 | 40 | 15 | 223 | 118 | 39 |
| chr4:10099335\|10105610 | hsa_circ_0003550 | 29 | 20 | 8 | 19 | 14 | 12 | 27 | 47 | 19 | 226 | 156 | 69 |
| chr3:93714718\|93722752 | hsa_circ_0006135 | 14 | 6 | 4 | 11 | 11 | 2 | 13 | 46 | 14 | 161 | 61 | 21 |
| chr3:8977555\|8983488 | hsa_circ_0001264 | 35 | 28 | 17 | 26 | 17 | 14 | 17 | 24 | 19 | 260 | 98 | 51 |
| chr3:71739161\|71759635 | hsa_circ_0066536 | 8 | 8 | 10 | 7 | 5 | 4 | 8 | 4 | 8 | 89 | 30 | 18 |
| chr3:71090479\|71102924 | hsa_circ_0008234 | 16 | 8 | 5 | 12 | 17 | 8 | 20 | 17 | 17 | 95 | 77 | 34 |
| chr3:57875768\|57882659 | hsa_circ_0002693 | 8 | 14 | 3 | 5 | 4 | 2 | 2 | 10 | 4 | 25 | 28 | 8 |
| chr3:57618992\|57627474 | #N/A | 13 | 17 | 2 | 8 | 12 | 6 | 7 | 10 | 5 | 113 | 50 | 13 |
| chr3:56694759\|56707753 | hsa_circ_0001315 | 30 | 18 | 8 | 8 | 14 | 7 | 17 | 7 | 9 | 122 | 58 | 26 |
| chr3:56661065\|56662642 | hsa_circ_0001314 | 65 | 50 | 20 | 31 | 51 | 19 | 43 | 68 | 29 | 739 | 254 | 111 |
| chr3:56626998\|56628056 | hsa_circ_0001313 | 96 | 59 | 46 | 38 | 58 | 35 | 63 | 74 | 36 | 658 | 211 | 109 |
| chr3:56600622\|56601081 | hsa_circ_0001312 | 161 | 111 | 51 | 98 | 105 | 72 | 123 | 108 | 76 | 1388 | 544 | 183 |
| chr3:52771602\|52775515 | hsa_circ_0001309 | 30 | 22 | 8 | 21 | 20 | 8 | 60 | 43 | 21 | 353 | 177 | 58 |
| chr3:52446827\|52448603 | hsa_circ_0004912 | 24 | 33 | 4 | 17 | 15 | 7 | 19 | 32 | 7 | 157 | 86 | 41 |
| chr3:5212188\|5216099 | hsa_circ_0005046 | 6 | 7 | 3 | 3 | 2 | 4 | 4 | 13 | 6 | 73 | 31 | 14 |
| chr3:51575605\|51624575 | hsa_circ_0006909 | 2 | 6 | 2 | 2 | 5 | 2 | 4 | 9 | 4 | 17 | 6 | 4 |
| chr3:51575514\|51624575 | hsa_circ_0001307 | 5 | 10 | 3 | 10 | 11 | 4 | 24 | 22 | 10 | 80 | 52 | 14 |
| chr3:50145503\|50145737 | hsa_circ_0001305 | 10 | 5 | 2 | 12 | 18 | 7 | 3 | 8 | 4 | 144 | 68 | 7 |
| chr3:50102464\|50103935 | hsa_circ_0006531 | 29 | 19 | 4 | 21 | 22 | 10 | 40 | 41 | 23 | 230 | 137 | 35 |
| chr3:50004903\|50012825 | hsa_circ_0065769 | 12 | 18 | 6 | 10 | 2 | 5 | 6 | 8 | 3 | 73 | 39 | 5 |
| chr3:49372453\|49373029 | hsa_circ_0001301 | 43 | 42 | 27 | 34 | 31 | 43 | 33 | 54 | 48 | 288 | 156 | 85 |
| chr3:48491443\|48495818 | hsa_circ_0065384 | 11 | 3 | 3 | 12 | 8 | 3 | 2 | 8 | 3 | 104 | 28 | 8 |
| chr3:48491443\|48493305 | #N/A | 18 | 12 | 4 | 6 | 8 | 5 | 6 | 7 | 6 | 87 | 28 | 19 |
| chr3:47139445\|47147610 | hsa_circ_0001290 | 150 | 103 | 42 | 119 | 129 | 50 | 200 | 276 | 158 | 1893 | 859 | 329 |
| chr3:47139445\|47144913 | hsa_circ_0001289 | 75 | 72 | 28 | 38 | 58 | 24 | 52 | 82 | 39 | 654 | 326 | 134 |
| chr3:47103653\|47108608 | hsa_circ_0065159 | 19 | 7 | 10 | 5 | 11 | 8 | 13 | 15 | 11 | 89 | 49 | 22 |
| chr3:47098311\|47108608 | hsa_circ_0002569 | 12 | 5 | 2 | 4 | 10 | 4 | 5 | 7 | 8 | 43 | 28 | 8 |
| chr3:47079156\|47108608 | hsa_circ_0004692 | 12 | 12 | 4 | 10 | 6 | 2 | 5 | 5 | 4 | 38 | 30 | 2 |
| chr3:44871463\|44882730 | #N/A | 11 | 6 | 3 | 2 | 4 | 3 | 2 | 2 | 2 | 53 | 18 | 2 |
| chr3:44835709\|44835918 | hsa_circ_0065034 | 27 | 31 | 12 | 15 | 16 | 20 | 24 | 26 | 24 | 92 | 93 | 35 |
| chr3:3178944\|3182332 | hsa_circ_0004631 | 6 | 8 | 3 | 4 | 3 | 2 | 3 | 11 | 4 | 90 | 28 | 10 |
| chr3:31617888\|31621588 | hsa_circ_0001278 | 33 | 42 | 9 | 7 | 27 | 12 | 37 | 46 | 24 | 404 | 194 | 73 |
| chr3:20178434\|20181856 | #N/A | 8 | 6 | 6 | 5 | 2 | 5 | 3 | 3 | 2 | 70 | 17 | 12 |
| chr3:20113076\|20113951 | #N/A | 21 | 20 | 7 | 24 | 43 | 11 | 12 | 23 | 5 | 219 | 126 | 25 |
| chr3:20112856\|20113951 | #N/A | 11 | 10 | 2 | 10 | 9 | 7 | 11 | 13 | 12 | 77 | 78 | 9 |
| chr3:20017093\|20019895 | #N/A | 5 | 7 | 4 | 5 | 4 | 2 | 3 | 4 | 4 | 52 | 19 | 10 |
| chr3:197597031\|197602646 | hsa_circ_0005873 | 12 | 3 | 4 | 10 | 12 | 5 | 10 | 11 | 8 | 64 | 40 | 15 |
| chr3:197592983\|197598333 | hsa_circ_0002319 | 20 | 17 | 4 | 29 | 38 | 9 | 33 | 44 | 10 | 172 | 99 | 27 |
| chr3:197592294\|197602646 | hsa_circ_0008351 | 129 | 136 | 47 | 120 | 141 | 73 | 151 | 202 | 86 | 1393 | 853 | 196 |
| chr3:197592294\|197598333 | hsa_circ_0008106 | 23 | 20 | 6 | 26 | 14 | 11 | 26 | 21 | 18 | 176 | 94 | 32 |
| chr3:197592294\|197593090 | hsa_circ_0008439 | 31 | 30 | 20 | 36 | 66 | 23 | 7 | 13 | 10 | 380 | 162 | 59 |
| chr3:197541779\|197547301 | hsa_circ_0002266 | 36 | 35 | 8 | 21 | 26 | 13 | 9 | 27 | 7 | 330 | 88 | 38 |
| chr3:196863413\|196876667 | #N/A | 17 | 15 | 4 | 10 | 8 | 17 | 12 | 22 | 14 | 125 | 47 | 12 |
| chr3:196533450\|196539722 | hsa_circ_0004950 | 29 | 22 | 4 | 3 | 4 | 6 | 8 | 12 | 12 | 142 | 63 | 10 |
| chr3:196533450\|196534785 | hsa_circ_0003036 | 4 | 3 | 4 | 5 | 5 | 4 | 6 | 4 | 3 | 27 | 34 | 12 |
| chr3:196214270\|196215554 | #N/A | 12 | 14 | 2 | 20 | 25 | 14 | 7 | 18 | 8 | 34 | 24 | 3 |
| chr3:196118684\|196134264 | #N/A | 11 | 3 | 6 | 10 | 13 | 6 | 15 | 9 | 5 | 98 | 50 | 14 |
| chr3:196118684\|196129890 | hsa_circ_0001380 | 361 | 317 | 104 | 312 | 298 | 168 | 98 | 146 | 77 | 2584 | 1224 | 359 |
| chr3:196118684\|196120490 | hsa_circ_0005051 | 46 | 44 | 19 | 45 | 49 | 26 | 12 | 9 | 2 | 592 | 237 | 36 |
| chr3:195800801\|195803993 | hsa_circ_0068630 | 8 | 13 | 4 | 12 | 21 | 7 | 32 | 33 | 8 | 138 | 60 | 29 |
| chr3:195800801\|195802231 | hsa_circ_0068629 | 20 | 31 | 5 | 14 | 24 | 5 | 22 | 24 | 8 | 185 | 103 | 38 |
| chr3:195798874\|195803993 | hsa_circ_0068628 | 10 | 5 | 2 | 6 | 6 | 4 | 8 | 17 | 8 | 89 | 53 | 17 |
| chr3:195791180\|195796439 | #N/A | 15 | 18 | 10 | 16 | 19 | 7 | 26 | 36 | 26 | 212 | 119 | 52 |
| chr3:195785155\|195787118 | hsa_circ_0068610 | 101 | 96 | 38 | 102 | 107 | 62 | 32 | 31 | 23 | 686 | 396 | 100 |
| chr3:195780289\|195803993 | hsa_circ_0068606 | 56 | 47 | 19 | 9 | 14 | 5 | 11 | 16 | 13 | 124 | 70 | 16 |
| chr3:195780289\|195789516 | hsa_circ_0068605 | 14 | 12 | 5 | 9 | 6 | 2 | 11 | 15 | 10 | 123 | 55 | 22 |
| chr3:195780289\|195787118 | hsa_circ_0068604 | 9 | 16 | 2 | 12 | 9 | 5 | 15 | 17 | 5 | 132 | 66 | 15 |
| chr3:195101738\|195133645 | hsa_circ_0009013 | 16 | 10 | 3 | 9 | 11 | 3 | 17 | 10 | 11 | 124 | 66 | 27 |
| chr3:195101738\|195112876 | hsa_circ_0007331 | 33 | 29 | 9 | 29 | 26 | 16 | 20 | 19 | 14 | 188 | 126 | 58 |
| chr3:195047614\|195066046 | hsa_circ_0007347 | 6 | 10 | 2 | 5 | 5 | 3 | 8 | 12 | 7 | 50 | 37 | 15 |
| chr3:193374869\|193385069 | #N/A | 40 | 48 | 9 | 18 | 12 | 8 | 20 | 11 | 6 | 195 | 80 | 28 |
| chr3:182681669\|182683541 | #N/A | 7 | 15 | 7 | 2 | 7 | 2 | 11 | 25 | 4 | 116 | 66 | 29 |
| chr3:182679014\|182683541 | #N/A | 46 | 28 | 17 | 18 | 18 | 18 | 30 | 31 | 22 | 497 | 171 | 87 |
| chr3:180651122\|180653019 | hsa_circ_0068129 | 25 | 37 | 6 | 24 | 25 | 13 | 8 | 10 | 3 | 277 | 114 | 15 |
| chr3:179310432\|179319578 | hsa_circ_0005292 | 31 | 20 | 6 | 8 | 16 | 4 | 16 | 27 | 20 | 149 | 87 | 49 |
| chr3:179131200\|179137293 | hsa_circ_0001364 | 8 | 7 | 8 | 12 | 2 | 3 | 3 | 9 | 10 | 61 | 37 | 6 |
| chr3:171965323\|171969331 | hsa_circ_0006156 | 37 | 50 | 12 | 59 | 69 | 46 | 42 | 46 | 29 | 287 | 184 | 56 |
| chr3:169863211\|169867032 | hsa_circ_0001360 | 116 | 94 | 28 | 206 | 199 | 143 | 132 | 73 | 78 | 558 | 339 | 102 |
| chr3:169854207\|169867032 | hsa_circ_0001359 | 211 | 192 | 70 | 143 | 167 | 87 | 193 | 282 | 156 | 1738 | 992 | 287 |
| chr3:169854207\|169863309 | hsa_circ_0001965 | 15 | 18 | 7 | 14 | 18 | 14 | 42 | 41 | 14 | 243 | 138 | 45 |
| chr3:169840379\|169843795 | hsa_circ_0007854 | 68 | 72 | 29 | 69 | 62 | 43 | 68 | 62 | 36 | 499 | 296 | 69 |
| chr3:169835014\|169840532 | hsa_circ_0004616 | 10 | 6 | 2 | 12 | 12 | 8 | 25 | 11 | 9 | 136 | 49 | 24 |
| chr3:167754624\|167759262 | hsa_circ_0004086 | 31 | 21 | 10 | 10 | 23 | 13 | 25 | 29 | 15 | 220 | 108 | 29 |
| chr3:167422630\|167443261 | hsa_circ_0067865 | 4 | 9 | 2 | 6 | 7 | 4 | 9 | 15 | 11 | 91 | 35 | 16 |
| chr3:167422630\|167438061 | hsa_circ_0067864 | 8 | 9 | 2 | 4 | 8 | 5 | 4 | 7 | 4 | 66 | 36 | 12 |
| chr3:167413384\|167443261 | #N/A | 10 | 16 | 4 | 5 | 8 | 9 | 5 | 13 | 7 | 110 | 56 | 20 |
| chr3:157839892\|157841780 | hsa_circ_0001355 | 43 | 68 | 12 | 32 | 28 | 15 | 47 | 41 | 30 | 333 | 202 | 79 |
| chr3:156411809\|156413814 | hsa_circ_0006999 | 8 | 9 | 2 | 16 | 11 | 4 | 14 | 6 | 11 | 118 | 48 | 16 |
| chr3:155628481\|155632359 | hsa_circ_0002832 | 11 | 7 | 6 | 4 | 4 | 7 | 16 | 24 | 7 | 127 | 54 | 19 |
| chr3:152132730\|152150709 | hsa_circ_0001349 | 7 | 19 | 7 | 15 | 15 | 8 | 10 | 28 | 10 | 174 | 113 | 34 |
| chr3:150834125\|150845771 | hsa_circ_0067735 | 15 | 14 | 5 | 11 | 9 | 3 | 12 | 26 | 16 | 167 | 96 | 31 |
| chr3:149563798\|149639014 | hsa_circ_0001346 | 54 | 62 | 22 | 29 | 17 | 8 | 25 | 26 | 18 | 323 | 159 | 46 |
| chr3:149563798\|149589941 | hsa_circ_0067716 | 15 | 21 | 8 | 16 | 21 | 7 | 25 | 15 | 11 | 248 | 120 | 41 |
| chr3:148309971\|148384889 | #N/A | 8 | 7 | 6 | 9 | 8 | 4 | 5 | 7 | 4 | 39 | 27 | 7 |
| chr3:148303909\|148384889 | #N/A | 4 | 7 | 4 | 4 | 2 | 4 | 8 | 9 | 2 | 51 | 33 | 8 |
| chr3:148303909\|148310052 | #N/A | 38 | 32 | 10 | 37 | 40 | 15 | 16 | 16 | 4 | 265 | 115 | 25 |
| chr3:148164053\|148216757 | #N/A | 15 | 11 | 5 | 7 | 13 | 4 | 22 | 30 | 16 | 148 | 88 | 32 |
| chr3:148164053\|148173318 | #N/A | 124 | 93 | 22 | 86 | 94 | 40 | 103 | 118 | 49 | 1186 | 441 | 142 |
| chr3:143704385\|143708679 | hsa_circ_0006731 | 34 | 39 | 20 | 27 | 28 | 20 | 65 | 95 | 45 | 504 | 224 | 147 |
| chr3:142144064\|142145683 | hsa_circ_0067620 | 50 | 45 | 14 | 44 | 33 | 16 | 38 | 43 | 30 | 389 | 158 | 70 |
| chr3:142116171\|142123918 | hsa_circ_0067619 | 40 | 40 | 16 | 30 | 13 | 13 | 17 | 13 | 14 | 295 | 108 | 56 |
| chr3:141811903\|141820683 | hsa_circ_0008304 | 76 | 68 | 22 | 63 | 63 | 56 | 10 | 23 | 13 | 803 | 261 | 59 |
| chr3:141678516\|141688983 | hsa_circ_0067593 | 9 | 5 | 8 | 7 | 7 | 3 | 11 | 10 | 5 | 89 | 36 | 31 |
| chr3:141272699\|141278837 | hsa_circ_0005372 | 20 | 17 | 7 | 22 | 15 | 5 | 17 | 21 | 19 | 136 | 97 | 35 |
| chr3:141231005\|141259451 | hsa_circ_0067582 | 10 | 5 | 2 | 2 | 5 | 4 | 4 | 5 | 3 | 75 | 35 | 14 |
| chr3:136323151\|136323315 | hsa_circ_0001342 | 20 | 13 | 12 | 20 | 12 | 5 | 23 | 44 | 32 | 149 | 93 | 43 |
| chr3:133907681\|133914026 | hsa_circ_0005768 | 25 | 8 | 4 | 11 | 11 | 5 | 11 | 13 | 7 | 151 | 67 | 25 |
| chr3:129177442\|129183624 | #N/A | 5 | 8 | 3 | 13 | 5 | 6 | 4 | 15 | 10 | 56 | 55 | 15 |
| chr3:121563300\|121576003 | hsa_circ_0066970 | 3 | 6 | 4 | 3 | 4 | 3 | 3 | 3 | 3 | 56 | 24 | 8 |
| chr3:121215655\|121217517 | #N/A | 15 | 10 | 3 | 9 | 9 | 6 | 6 | 19 | 14 | 120 | 71 | 34 |
| chr3:119219542\|119222868 | hsa_circ_0001329 | 41 | 43 | 23 | 38 | 52 | 25 | 91 | 69 | 39 | 552 | 239 | 88 |
| chr3:107429299\|107435696 | hsa_circ_0001324 | 12 | 9 | 4 | 11 | 9 | 3 | 14 | 17 | 7 | 137 | 61 | 37 |
| chr3:101389974\|101391057 | hsa_circ_0003179 | 23 | 16 | 5 | 16 | 23 | 2 | 36 | 44 | 18 | 277 | 99 | 71 |
| chr3:100451352\|100455560 | hsa_circ_0008839 | 3 | 8 | 2 | 3 | 2 | 3 | 10 | 3 | 4 | 49 | 24 | 7 |
| chr3:10015312\|10019130 | hsa_circ_0064196 | 7 | 3 | 6 | 2 | 4 | 2 | 6 | 8 | 2 | 55 | 16 | 7 |
| chr22:50810449\|50832564 | hsa_circ_0001258 | 9 | 6 | 2 | 4 | 7 | 2 | 9 | 12 | 5 | 106 | 53 | 14 |
| chr22:45574119\|45574781 | hsa_circ_0002077 | 23 | 18 | 14 | 16 | 30 | 10 | 14 | 17 | 7 | 175 | 61 | 21 |
| chr22:41738533\|41739580 | hsa_circ_0001236 | 9 | 15 | 10 | 15 | 9 | 11 | 7 | 7 | 9 | 77 | 40 | 9 |
| chr22:41531817\|41536261 | hsa_circ_0003184 | 42 | 57 | 16 | 51 | 69 | 35 | 98 | 88 | 48 | 536 | 296 | 87 |
| chr22:38895405\|38897285 | hsa_circ_0001230 | 6 | 2 | 5 | 9 | 10 | 5 | 8 | 6 | 4 | 63 | 44 | 9 |
| chr22:32874968\|32875262 | hsa_circ_0008832 | 26 | 32 | 9 | 18 | 28 | 12 | 19 | 25 | 21 | 235 | 90 | 27 |
| chr22:30374431\|30394846 | hsa_circ_0009055 | 22 | 9 | 5 | 6 | 13 | 6 | 21 | 34 | 12 | 159 | 93 | 30 |
| chr22:30374431\|30387659 | hsa_circ_0002954 | 21 | 13 | 8 | 18 | 16 | 3 | 36 | 39 | 21 | 249 | 142 | 45 |
| chr22:30366965\|30375041 | hsa_circ_0006303 | 4 | 4 | 2 | 4 | 2 | 5 | 2 | 7 | 2 | 51 | 20 | 12 |
| chr22:29682912\|29683123 | hsa_circ_0008044 | 6 | 14 | 6 | 10 | 20 | 6 | 8 | 6 | 4 | 37 | 42 | 15 |
| chr22:29120965\|29121355 | hsa_circ_0002931 | 35 | 29 | 5 | 26 | 26 | 17 | 28 | 28 | 12 | 193 | 100 | 40 |
| chr22:28306952\|28310335 | hsa_circ_0003056 | 24 | 12 | 8 | 17 | 16 | 18 | 15 | 16 | 12 | 168 | 105 | 15 |
| chr22:26853825\|26854543 | hsa_circ_0005175 | 21 | 27 | 6 | 9 | 21 | 10 | 12 | 13 | 6 | 77 | 53 | 21 |
| chr22:24698163\|24709434 | hsa_circ_0007312 | 16 | 28 | 6 | 19 | 11 | 8 | 16 | 14 | 18 | 149 | 100 | 30 |
| chr22:22160139\|22162135 | hsa_circ_0008870 | 171 | 138 | 63 | 139 | 133 | 68 | 168 | 209 | 121 | 1496 | 700 | 234 |
| chr22:21288067\|21288532 | hsa_circ_0001206 | 14 | 9 | 6 | 12 | 7 | 3 | 9 | 11 | 12 | 89 | 52 | 13 |
| chr21:47768926\|47769734 | hsa_circ_0002903 | 466 | 372 | 144 | 463 | 472 | 226 | 422 | 575 | 347 | 5136 | 2494 | 688 |
| chr21:46275125\|46281186 | hsa_circ_0001200 | 47 | 49 | 17 | 37 | 46 | 21 | 64 | 111 | 65 | 417 | 210 | 91 |
| chr21:40600426\|40601362 | hsa_circ_0001195 | 17 | 24 | 10 | 8 | 17 | 14 | 6 | 3 | 14 | 106 | 48 | 9 |
| chr21:40578034\|40584633 | #N/A | 99 | 79 | 35 | 45 | 46 | 25 | 62 | 77 | 31 | 887 | 349 | 105 |
| chr21:38792601\|38845182 | hsa_circ_0005955 | 35 | 29 | 6 | 19 | 22 | 12 | 26 | 56 | 16 | 233 | 104 | 47 |
| chr21:38792601\|38794168 | hsa_circ_0001190 | 26 | 15 | 9 | 22 | 22 | 15 | 5 | 11 | 6 | 205 | 136 | 38 |
| chr21:37734481\|37736557 | #N/A | 49 | 65 | 6 | 48 | 40 | 32 | 15 | 17 | 10 | 297 | 187 | 61 |
| chr21:37716877\|37721706 | #N/A | 15 | 11 | 10 | 8 | 9 | 6 | 10 | 17 | 7 | 129 | 61 | 18 |
| chr21:37713493\|37717005 | #N/A | 3 | 5 | 4 | 11 | 2 | 6 | 8 | 5 | 4 | 51 | 21 | 5 |
| chr21:37711077\|37717005 | hsa_circ_0001189 | 746 | 614 | 293 | 413 | 494 | 248 | 634 | 955 | 496 | 7152 | 3178 | 1140 |
| chr21:37619815\|37620866 | hsa_circ_0001187 | 27 | 14 | 8 | 16 | 24 | 10 | 25 | 24 | 19 | 186 | 86 | 21 |
| chr21:36206707\|36231875 | hsa_circ_0002360 | 11 | 8 | 4 | 38 | 38 | 14 | 11 | 13 | 11 | 49 | 44 | 12 |
| chr21:35138179\|35140132 | #N/A | 28 | 20 | 4 | 11 | 9 | 11 | 6 | 8 | 5 | 122 | 52 | 23 |
| chr21:34931536\|34932081 | #N/A | 17 | 27 | 10 | 11 | 13 | 15 | 28 | 22 | 24 | 205 | 107 | 28 |
| chr21:34804484\|34805178 | hsa_circ_0001185 | 13 | 16 | 4 | 11 | 11 | 3 | 13 | 16 | 5 | 156 | 57 | 17 |
| chr21:17205667\|17214859 | hsa_circ_0005238 | 77 | 59 | 14 | 39 | 51 | 26 | 79 | 59 | 36 | 602 | 260 | 89 |
| chr21:17135210\|17138460 | hsa_circ_0001178 | 190 | 159 | 58 | 126 | 149 | 86 | 152 | 183 | 127 | 1281 | 632 | 207 |
| chr21:16386665\|16415895 | hsa_circ_0004771 | 18 | 21 | 9 | 14 | 22 | 10 | 15 | 10 | 5 | 152 | 97 | 22 |
| chr20:62559688\|62562375 | hsa_circ_0007609 | 12 | 20 | 4 | 21 | 23 | 4 | 27 | 31 | 21 | 130 | 124 | 37 |
| chr20:60714131\|60716000 | hsa_circ_0003456 | 7 | 8 | 4 | 11 | 6 | 5 | 14 | 25 | 11 | 98 | 54 | 32 |
| chr20:57014001\|57016139 | hsa_circ_0001173 | 160 | 166 | 59 | 113 | 129 | 87 | 130 | 161 | 80 | 1157 | 512 | 153 |
| chr20:5538591\|5539465 | #N/A | 11 | 4 | 4 | 5 | 5 | 11 | 14 | 14 | 3 | 101 | 44 | 9 |
| chr20:54961313\|54963258 | hsa_circ_0060950 | 24 | 33 | 8 | 18 | 15 | 8 | 37 | 35 | 25 | 214 | 152 | 40 |
| chr20:54956489\|54961589 | hsa_circ_0008777 | 11 | 4 | 6 | 3 | 5 | 3 | 14 | 9 | 6 | 85 | 41 | 14 |
| chr20:54956489\|54959380 | hsa_circ_0004887 | 105 | 78 | 22 | 41 | 49 | 32 | 64 | 71 | 45 | 837 | 246 | 115 |
| chr20:47782534\|47790806 | hsa_circ_0060778 | 10 | 8 | 2 | 7 | 11 | 5 | 7 | 10 | 12 | 104 | 41 | 11 |
| chr20:45923410\|45927691 | hsa_circ_0060624 | 14 | 16 | 3 | 9 | 14 | 2 | 11 | 15 | 7 | 152 | 73 | 24 |
| chr20:45905058\|45916028 | hsa_circ_0004212 | 35 | 20 | 7 | 20 | 17 | 9 | 30 | 41 | 34 | 246 | 105 | 54 |
| chr20:45891032\|45912392 | hsa_circ_0060611 | 7 | 2 | 3 | 2 | 5 | 3 | 10 | 7 | 3 | 38 | 9 | 10 |
| chr20:45874752\|45878183 | hsa_circ_0001163 | 17 | 12 | 7 | 6 | 11 | 4 | 18 | 30 | 9 | 103 | 41 | 33 |
| chr20:45874752\|45875261 | hsa_circ_0005996 | 19 | 18 | 8 | 23 | 17 | 6 | 26 | 32 | 27 | 179 | 84 | 41 |
| chr20:45855946\|45865260 | #N/A | 13 | 7 | 5 | 19 | 18 | 6 | 12 | 7 | 5 | 96 | 45 | 14 |
| chr20:43132456\|43135637 | hsa_circ_0004860 | 6 | 7 | 3 | 8 | 13 | 7 | 19 | 5 | 9 | 90 | 39 | 10 |
| chr20:42331130\|42333998 | hsa_circ_0006332 | 16 | 31 | 4 | 39 | 42 | 25 | 38 | 86 | 40 | 202 | 189 | 44 |
| chr20:42331130\|42331543 | hsa_circ_0060461 | 17 | 6 | 4 | 21 | 30 | 7 | 25 | 27 | 14 | 94 | 103 | 40 |
| chr20:40161689\|40179999 | hsa_circ_0001159 | 25 | 18 | 9 | 16 | 16 | 7 | 13 | 13 | 13 | 153 | 67 | 24 |
| chr20:39741422\|39742795 | #N/A | 11 | 9 | 3 | 12 | 2 | 4 | 5 | 13 | 2 | 52 | 32 | 13 |
| chr20:39721112\|39729993 | #N/A | 128 | 94 | 48 | 47 | 35 | 22 | 21 | 38 | 26 | 456 | 166 | 55 |
| chr20:3925824\|3955047 | hsa_circ_0005736 | 30 | 29 | 3 | 28 | 32 | 20 | 28 | 22 | 10 | 279 | 118 | 27 |
| chr20:36694205\|36694658 | hsa_circ_0001155 | 5 | 9 | 2 | 10 | 8 | 2 | 12 | 11 | 4 | 69 | 23 | 12 |
| chr20:35695127\|35696589 | hsa_circ_0060238 | 9 | 22 | 11 | 6 | 10 | 9 | 11 | 7 | 5 | 195 | 57 | 20 |
| chr20:35693827\|35696589 | #N/A | 14 | 15 | 7 | 8 | 10 | 3 | 12 | 6 | 7 | 167 | 46 | 25 |
| chr20:35689506\|35693876 | hsa_circ_0060237 | 22 | 13 | 6 | 13 | 14 | 9 | 11 | 28 | 13 | 208 | 90 | 39 |
| chr20:35689506\|35690673 | hsa_circ_0060236 | 18 | 11 | 7 | 10 | 22 | 6 | 29 | 35 | 16 | 188 | 90 | 26 |
| chr20:35684549\|35693876 | hsa_circ_0001153 | 29 | 14 | 6 | 9 | 16 | 6 | 13 | 16 | 2 | 203 | 44 | 18 |
| chr20:34388016\|34389527 | #N/A | 11 | 9 | 7 | 12 | 6 | 8 | 9 | 5 | 4 | 58 | 25 | 6 |
| chr20:34317234\|34320057 | hsa_circ_0008817 | 178 | 178 | 72 | 119 | 141 | 71 | 109 | 162 | 89 | 1216 | 615 | 221 |
| chr20:34309662\|34313077 | hsa_circ_0001148 | 21 | 10 | 2 | 8 | 17 | 8 | 23 | 30 | 25 | 204 | 76 | 48 |
| chr20:34304662\|34313077 | hsa_circ_0001147 | 20 | 15 | 9 | 12 | 10 | 11 | 23 | 38 | 24 | 214 | 116 | 47 |
| chr20:34302107\|34313077 | hsa_circ_0004870 | 28 | 28 | 6 | 16 | 23 | 12 | 33 | 47 | 30 | 260 | 118 | 45 |
| chr20:33954360\|33971936 | hsa_circ_0004994 | 6 | 4 | 3 | 2 | 6 | 4 | 7 | 10 | 2 | 36 | 23 | 5 |
| chr20:33068404\|33069011 | hsa_circ_0008686 | 7 | 8 | 3 | 3 | 8 | 2 | 3 | 5 | 2 | 46 | 15 | 7 |
| chr20:33065577\|33067594 | hsa_circ_0059958 | 6 | 13 | 3 | 5 | 6 | 6 | 3 | 3 | 9 | 78 | 38 | 9 |
| chr20:33057853\|33069011 | hsa_circ_0005868 | 15 | 6 | 9 | 7 | 11 | 3 | 7 | 7 | 5 | 128 | 55 | 15 |
| chr20:32619328\|32621124 | hsa_circ_0004133 | 19 | 23 | 8 | 26 | 24 | 11 | 13 | 4 | 7 | 287 | 162 | 45 |
| chr20:32617575\|32619410 | #N/A | 13 | 13 | 2 | 15 | 12 | 2 | 3 | 9 | 4 | 32 | 25 | 9 |
| chr20:30954187\|30956926 | hsa_circ_0001136 | 36 | 42 | 18 | 45 | 57 | 31 | 8 | 25 | 11 | 316 | 169 | 58 |
| chr20:30380538\|30382351 | hsa_circ_0009098 | 12 | 2 | 6 | 5 | 7 | 3 | 10 | 11 | 6 | 74 | 41 | 13 |
| chr20:2967411\|2969120 | hsa_circ_0001127 | 3 | 4 | 3 | 7 | 3 | 4 | 13 | 6 | 4 | 26 | 14 | 9 |
| chr20:2944918\|2945848 | hsa_circ_0006117 | 389 | 295 | 141 | 279 | 343 | 157 | 455 | 530 | 297 | 3660 | 1677 | 573 |
| chr20:2928628\|2945848 | hsa_circ_0005265 | 73 | 49 | 30 | 42 | 46 | 24 | 75 | 76 | 44 | 586 | 258 | 91 |
| chr20:10536879\|10541468 | hsa_circ_0005704 | 26 | 13 | 10 | 11 | 17 | 6 | 11 | 18 | 22 | 135 | 73 | 35 |
| chr2:99802640\|99812219 | hsa_circ_0001051 | 11 | 13 | 8 | 3 | 7 | 3 | 4 | 4 | 2 | 97 | 28 | 12 |
| chr2:99786013\|99787892 | hsa_circ_0001050 | 47 | 35 | 27 | 41 | 41 | 26 | 22 | 25 | 19 | 462 | 152 | 50 |
| chr2:98430434\|98435184 | #N/A | 17 | 17 | 6 | 9 | 7 | 2 | 16 | 7 | 6 | 93 | 27 | 13 |
| chr2:97024783\|97026448 | hsa_circ_0007532 | 6 | 9 | 2 | 4 | 3 | 2 | 5 | 3 | 2 | 64 | 25 | 5 |
| chr2:9083316\|9137472 | #N/A | 6 | 11 | 7 | 7 | 3 | 3 | 8 | 17 | 7 | 120 | 31 | 10 |
| chr2:9083316\|9127890 | #N/A | 21 | 13 | 7 | 22 | 12 | 8 | 16 | 19 | 10 | 181 | 96 | 42 |
| chr2:9083316\|9114564 | #N/A | 52 | 58 | 19 | 57 | 62 | 28 | 70 | 71 | 42 | 530 | 236 | 100 |
| chr2:9083316\|9102747 | #N/A | 35 | 23 | 14 | 32 | 30 | 17 | 39 | 48 | 17 | 272 | 134 | 42 |
| chr2:9083316\|9098771 | hsa_circ_0007334 | 740 | 745 | 219 | 575 | 655 | 447 | 122 | 179 | 123 | 2991 | 1792 | 632 |
| chr2:9079950\|9098771 | hsa_circ_0000973 | 182 | 134 | 40 | 110 | 116 | 44 | 186 | 206 | 88 | 1706 | 681 | 300 |
| chr2:9048751\|9114564 | #N/A | 4 | 13 | 5 | 6 | 8 | 4 | 7 | 15 | 3 | 99 | 41 | 7 |
| chr2:9048751\|9098771 | hsa_circ_0000972 | 363 | 385 | 123 | 332 | 308 | 162 | 303 | 336 | 203 | 3221 | 1604 | 555 |
| chr2:9028148\|9098771 | hsa_circ_0008847 | 28 | 24 | 6 | 15 | 19 | 14 | 21 | 29 | 19 | 190 | 128 | 49 |
| chr2:8910800\|8917022 | hsa_circ_0005315 | 14 | 21 | 4 | 13 | 19 | 8 | 15 | 11 | 15 | 148 | 64 | 21 |
| chr2:89100616\|89104394 | #N/A | 47 | 33 | 8 | 24 | 21 | 22 | 16 | 26 | 15 | 358 | 139 | 48 |
| chr2:86992150\|86993147 | #N/A | 3 | 2 | 2 | 4 | 10 | 6 | 9 | 8 | 4 | 60 | 37 | 9 |
| chr2:74300676\|74307718 | hsa_circ_0001032 | 18 | 15 | 7 | 11 | 14 | 9 | 20 | 25 | 7 | 135 | 74 | 38 |
| chr2:73784347\|73786269 | #N/A | 11 | 11 | 3 | 2 | 6 | 3 | 5 | 9 | 4 | 85 | 48 | 13 |
| chr2:72945232\|72960247 | hsa_circ_0009043 | 10 | 15 | 9 | 9 | 15 | 8 | 18 | 42 | 17 | 179 | 76 | 30 |
| chr2:71623271\|71645769 | hsa_circ_0055151 | 39 | 25 | 15 | 9 | 10 | 8 | 23 | 20 | 7 | 190 | 70 | 22 |
| chr2:70451686\|70463307 | hsa_circ_0006138 | 10 | 7 | 2 | 6 | 4 | 2 | 8 | 10 | 7 | 67 | 34 | 21 |
| chr2:68717322\|68753374 | hsa_circ_0055019 | 24 | 18 | 9 | 7 | 12 | 5 | 6 | 3 | 3 | 102 | 33 | 12 |
| chr2:68717322\|68740812 | hsa_circ_0003874 | 17 | 14 | 4 | 6 | 7 | 3 | 13 | 3 | 8 | 95 | 34 | 13 |
| chr2:68613634\|68621308 | hsa_circ_0055014 | 7 | 8 | 2 | 8 | 2 | 8 | 6 | 13 | 10 | 63 | 44 | 18 |
| chr2:64800080\|64812679 | hsa_circ_0006037 | 18 | 19 | 5 | 12 | 30 | 5 | 30 | 27 | 14 | 220 | 127 | 35 |
| chr2:64796226\|64800202 | hsa_circ_0054963 | 3 | 10 | 3 | 2 | 5 | 2 | 2 | 5 | 5 | 42 | 27 | 8 |
| chr2:64083440\|64085070 | hsa_circ_0001020 | 25 | 19 | 6 | 21 | 32 | 11 | 21 | 35 | 14 | 199 | 119 | 22 |
| chr2:61749746\|61761038 | hsa_circ_0001017 | 581 | 516 | 165 | 399 | 418 | 209 | 384 | 363 | 233 | 5622 | 1901 | 674 |
| chr2:61749746\|61753656 | hsa_circ_0001016 | 66 | 64 | 24 | 48 | 72 | 38 | 37 | 46 | 18 | 514 | 204 | 90 |
| chr2:61726848\|61729445 | #N/A | 18 | 8 | 10 | 7 | 15 | 3 | 11 | 17 | 4 | 105 | 58 | 15 |
| chr2:61505300\|61508377 | hsa_circ_0007493 | 41 | 25 | 18 | 20 | 34 | 10 | 44 | 36 | 26 | 288 | 166 | 39 |
| chr2:61406116\|61413632 | #N/A | 26 | 14 | 10 | 9 | 12 | 7 | 16 | 24 | 14 | 110 | 86 | 16 |
| chr2:61343114\|61345251 | hsa_circ_0007793 | 49 | 60 | 23 | 41 | 39 | 28 | 53 | 63 | 39 | 412 | 179 | 82 |
| chr2:61340905\|61345251 | hsa_circ_0001014 | 49 | 49 | 23 | 29 | 39 | 23 | 51 | 76 | 50 | 456 | 203 | 90 |
| chr2:61295984\|61297635 | #N/A | 6 | 6 | 2 | 11 | 4 | 3 | 7 | 14 | 4 | 78 | 34 | 19 |
| chr2:61118818\|61128218 | hsa_circ_0001011 | 12 | 11 | 7 | 6 | 11 | 8 | 6 | 8 | 2 | 80 | 34 | 5 |
| chr2:61009020\|61010344 | hsa_circ_0054683 | 14 | 7 | 4 | 15 | 15 | 8 | 5 | 7 | 6 | 88 | 42 | 18 |
| chr2:58449077\|58459247 | hsa_circ_0001009 | 157 | 176 | 40 | 110 | 132 | 70 | 151 | 167 | 101 | 1197 | 573 | 250 |
| chr2:58425729\|58459247 | hsa_circ_0004435 | 20 | 14 | 3 | 14 | 15 | 5 | 25 | 39 | 15 | 234 | 100 | 34 |
| chr2:58311224\|58316858 | #N/A | 119 | 92 | 33 | 61 | 63 | 33 | 87 | 72 | 44 | 1078 | 380 | 137 |
| chr2:55209651\|55214834 | hsa_circ_0001006 | 3 | 5 | 5 | 7 | 12 | 4 | 11 | 26 | 10 | 113 | 43 | 19 |
| chr2:54278095\|54284497 | hsa_circ_0004462 | 11 | 13 | 2 | 22 | 16 | 7 | 5 | 6 | 3 | 67 | 47 | 14 |
| chr2:48065998\|48066908 | hsa_circ_0003872 | 16 | 7 | 3 | 7 | 7 | 4 | 26 | 34 | 22 | 230 | 79 | 33 |
| chr2:45773871\|45789895 | hsa_circ_0000997 | 7 | 7 | 4 | 4 | 11 | 4 | 6 | 10 | 5 | 92 | 33 | 14 |
| chr2:44931422\|44942492 | hsa_circ_0006530 | 3 | 2 | 2 | 5 | 3 | 4 | 5 | 5 | 4 | 41 | 20 | 4 |
| chr2:44729828\|44732869 | hsa_circ_0000996 | 18 | 12 | 10 | 22 | 21 | 10 | 13 | 10 | 8 | 127 | 63 | 22 |
| chr2:44436349\|44436466 | hsa_circ_0054406 | 41 | 25 | 10 | 43 | 29 | 17 | 18 | 2 | 4 | 318 | 189 | 14 |
| chr2:32312561\|32314674 | hsa_circ_0053406 | 8 | 10 | 11 | 16 | 13 | 3 | 13 | 10 | 8 | 146 | 76 | 32 |
| chr2:26502862\|26507072 | hsa_circ_0053082 | 2 | 5 | 2 | 3 | 2 | 2 | 6 | 6 | 5 | 56 | 27 | 4 |
| chr2:26486248\|26500028 | hsa_circ_0008257 | 16 | 18 | 7 | 24 | 16 | 9 | 24 | 22 | 20 | 148 | 81 | 35 |
| chr2:26350013\|26350820 | hsa_circ_0006596 | 13 | 5 | 4 | 4 | 5 | 3 | 6 | 4 | 4 | 57 | 23 | 10 |
| chr2:26321531\|26332775 | #N/A | 22 | 21 | 6 | 12 | 10 | 10 | 10 | 4 | 3 | 115 | 78 | 15 |
| chr2:26022254\|26029206 | hsa_circ_0004476 | 6 | 4 | 5 | 8 | 11 | 6 | 9 | 11 | 9 | 49 | 25 | 9 |
| chr2:24778884\|24807429 | #N/A | 6 | 4 | 3 | 4 | 6 | 5 | 2 | 3 | 3 | 59 | 19 | 6 |
| chr2:24778884\|24787299 | #N/A | 11 | 9 | 3 | 9 | 7 | 7 | 9 | 7 | 8 | 99 | 54 | 14 |
| chr2:24357989\|24369956 | hsa_circ_0000982 | 43 | 31 | 11 | 16 | 20 | 11 | 14 | 16 | 4 | 247 | 86 | 40 |
| chr2:242099747\|242102816 | #N/A | 12 | 12 | 5 | 14 | 8 | 7 | 8 | 17 | 4 | 140 | 44 | 16 |
| chr2:24181171\|24207701 | hsa_circ_0052967 | 7 | 7 | 11 | 2 | 7 | 5 | 4 | 8 | 3 | 87 | 32 | 10 |
| chr2:24181171\|24199945 | hsa_circ_0005970 | 26 | 21 | 9 | 13 | 20 | 10 | 20 | 30 | 17 | 240 | 102 | 36 |
| chr2:24103509\|24108699 | hsa_circ_0052963 | 40 | 30 | 16 | 21 | 22 | 12 | 16 | 17 | 12 | 246 | 72 | 43 |
| chr2:240929491\|240954277 | hsa_circ_0001119 | 9 | 5 | 5 | 18 | 16 | 4 | 14 | 15 | 7 | 100 | 56 | 30 |
| chr2:240929491\|240946787 | hsa_circ_0001118 | 22 | 24 | 13 | 17 | 29 | 8 | 16 | 23 | 19 | 171 | 99 | 38 |
| chr2:24046128\|24046439 | hsa_circ_0003828 | 46 | 44 | 11 | 55 | 46 | 12 | 53 | 42 | 43 | 334 | 209 | 56 |
| chr2:239090706\|239093928 | hsa_circ_0001116 | 49 | 37 | 23 | 28 | 34 | 13 | 37 | 81 | 36 | 516 | 277 | 101 |
| chr2:234296903\|234299129 | hsa_circ_0001112 | 23 | 15 | 6 | 22 | 31 | 8 | 24 | 28 | 6 | 191 | 104 | 28 |
| chr2:233675954\|233677201 | hsa_circ_0003937 | 6 | 8 | 2 | 11 | 7 | 8 | 6 | 2 | 5 | 29 | 36 | 10 |
| chr2:233651860\|233660931 | hsa_circ_0058733 | 15 | 14 | 8 | 6 | 2 | 2 | 8 | 3 | 6 | 33 | 19 | 11 |
| chr2:231307652\|231314970 | hsa_circ_0003922 | 33 | 20 | 19 | 23 | 20 | 9 | 28 | 41 | 17 | 254 | 131 | 49 |
| chr2:231222520\|231226412 | hsa_circ_0008382 | 8 | 9 | 6 | 14 | 11 | 3 | 6 | 23 | 14 | 123 | 66 | 19 |
| chr2:230723488\|230744844 | hsa_circ_0003273 | 10 | 8 | 2 | 6 | 3 | 2 | 5 | 4 | 5 | 54 | 26 | 16 |
| chr2:230723488\|230725247 | hsa_circ_0005689 | 12 | 15 | 3 | 5 | 10 | 3 | 10 | 7 | 8 | 79 | 24 | 13 |
| chr2:228193394\|228194499 | #N/A | 17 | 15 | 4 | 6 | 8 | 2 | 9 | 10 | 5 | 102 | 30 | 10 |
| chr2:227771509\|227779067 | hsa_circ_0058497 | 32 | 38 | 6 | 22 | 28 | 8 | 39 | 29 | 24 | 257 | 126 | 50 |
| chr2:227729320\|227779067 | hsa_circ_0058495 | 58 | 55 | 23 | 36 | 27 | 15 | 50 | 51 | 36 | 386 | 260 | 83 |
| chr2:227729320\|227732034 | hsa_circ_0058493 | 75 | 67 | 40 | 69 | 73 | 43 | 100 | 112 | 72 | 948 | 462 | 157 |
| chr2:225400245\|225422573 | hsa_circ_0008309 | 14 | 7 | 3 | 16 | 17 | 5 | 21 | 19 | 15 | 125 | 36 | 14 |
| chr2:208589524\|208607066 | hsa_circ_0002556 | 29 | 22 | 5 | 19 | 25 | 12 | 17 | 16 | 11 | 210 | 89 | 23 |
| chr2:203817282\|203820481 | hsa_circ_0004919 | 12 | 10 | 3 | 4 | 4 | 5 | 10 | 22 | 8 | 102 | 47 | 20 |
| chr2:203329532\|203332412 | hsa_circ_0003218 | 35 | 51 | 7 | 35 | 42 | 17 | 42 | 39 | 31 | 416 | 206 | 70 |
| chr2:203162102\|203162629 | hsa_circ_0003337 | 13 | 12 | 5 | 15 | 11 | 14 | 14 | 9 | 6 | 130 | 40 | 18 |
| chr2:202208893\|202216174 | #N/A | 83 | 81 | 43 | 66 | 82 | 41 | 67 | 74 | 55 | 592 | 321 | 85 |
| chr2:202207093\|202228896 | #N/A | 12 | 12 | 5 | 14 | 9 | 8 | 11 | 18 | 9 | 94 | 53 | 14 |
| chr2:202207093\|202216174 | #N/A | 52 | 42 | 22 | 25 | 31 | 24 | 34 | 50 | 26 | 305 | 153 | 52 |
| chr2:202195193\|202228896 | #N/A | 50 | 22 | 17 | 28 | 29 | 17 | 18 | 40 | 18 | 264 | 116 | 49 |
| chr2:202195193\|202216174 | #N/A | 17 | 12 | 6 | 7 | 15 | 8 | 6 | 10 | 3 | 82 | 39 | 6 |
| chr2:202195193\|202195556 | hsa_circ_0057753 | 49 | 28 | 11 | 27 | 29 | 25 | 32 | 15 | 10 | 409 | 184 | 31 |
| chr2:202172242\|202228896 | #N/A | 27 | 21 | 10 | 5 | 10 | 2 | 11 | 12 | 9 | 145 | 72 | 12 |
| chr2:202163961\|202228896 | #N/A | 15 | 17 | 6 | 11 | 7 | 4 | 5 | 9 | 4 | 43 | 26 | 13 |
| chr2:202163961\|202173973 | #N/A | 13 | 15 | 7 | 12 | 9 | 7 | 13 | 24 | 12 | 130 | 51 | 23 |
| chr2:202163468\|202164023 | #N/A | 21 | 15 | 8 | 18 | 35 | 16 | 24 | 8 | 5 | 324 | 131 | 15 |
| chr2:202010101\|202014558 | hsa_circ_0001092 | 86 | 78 | 33 | 64 | 66 | 42 | 34 | 29 | 19 | 711 | 356 | 66 |
| chr2:201721405\|201721708 | hsa_circ_0004001 | 74 | 82 | 24 | 62 | 57 | 41 | 20 | 20 | 19 | 482 | 190 | 83 |
| chr2:201718626\|201719809 | hsa_circ_0003941 | 14 | 16 | 6 | 10 | 14 | 10 | 13 | 7 | 9 | 82 | 66 | 6 |
| chr2:197027986\|197030458 | #N/A | 16 | 8 | 3 | 3 | 4 | 2 | 4 | 2 | 3 | 95 | 27 | 11 |
| chr2:191765290\|191789319 | hsa_circ_0001085 | 23 | 18 | 3 | 4 | 4 | 4 | 9 | 13 | 12 | 99 | 42 | 16 |
| chr2:183993015\|183998351 | hsa_circ_0057298 | 11 | 15 | 5 | 15 | 5 | 3 | 16 | 12 | 7 | 76 | 38 | 16 |
| chr2:183993015\|183995273 | hsa_circ_0004820 | 14 | 27 | 2 | 9 | 22 | 5 | 15 | 26 | 23 | 193 | 82 | 17 |
| chr2:182346325\|182350719 | #N/A | 11 | 7 | 3 | 5 | 13 | 3 | 19 | 14 | 9 | 67 | 42 | 9 |
| chr2:178096617\|178098999 | #N/A | 68 | 48 | 24 | 54 | 37 | 24 | 49 | 59 | 32 | 540 | 266 | 103 |
| chr2:176835492\|176860374 | hsa_circ_0005775 | 25 | 20 | 10 | 13 | 15 | 9 | 14 | 28 | 8 | 297 | 97 | 35 |
| chr2:175976296\|175986268 | hsa_circ_0007967 | 25 | 14 | 6 | 12 | 14 | 7 | 20 | 18 | 15 | 151 | 94 | 23 |
| chr2:172782047\|172809519 | hsa_circ_0008032 | 91 | 81 | 33 | 76 | 99 | 54 | 97 | 112 | 81 | 800 | 346 | 125 |
| chr2:172305191\|172314585 | hsa_circ_0005904 | 10 | 7 | 2 | 10 | 12 | 6 | 12 | 23 | 15 | 119 | 82 | 33 |
| chr2:172193963\|172196064 | hsa_circ_0057041 | 11 | 7 | 4 | 5 | 8 | 3 | 13 | 14 | 6 | 130 | 76 | 28 |
| chr2:170394523\|170401345 | #N/A | 10 | 13 | 9 | 14 | 10 | 3 | 18 | 15 | 4 | 138 | 62 | 13 |
| chr2:169018297\|169038600 | hsa_circ_0001079 | 6 | 10 | 3 | 8 | 3 | 4 | 13 | 16 | 14 | 108 | 61 | 20 |
| chr2:162036152\|162061304 | #N/A | 9 | 3 | 3 | 4 | 4 | 5 | 10 | 4 | 2 | 79 | 14 | 8 |
| chr2:162036125\|162061304 | hsa_circ_0005227 | 64 | 75 | 29 | 34 | 41 | 21 | 58 | 60 | 46 | 706 | 290 | 98 |
| chr2:15693550\|15698758 | hsa_circ_0005556 | 12 | 11 | 3 | 6 | 11 | 9 | 6 | 5 | 7 | 92 | 43 | 9 |
| chr2:15691617\|15698758 | hsa_circ_0003287 | 96 | 81 | 20 | 73 | 82 | 35 | 102 | 66 | 56 | 888 | 383 | 127 |
| chr2:15629018\|15674765 | hsa_circ_0007128 | 7 | 9 | 8 | 7 | 7 | 3 | 4 | 21 | 8 | 95 | 42 | 20 |
| chr2:15629018\|15651474 | hsa_circ_0000978 | 12 | 28 | 6 | 13 | 8 | 3 | 23 | 16 | 14 | 174 | 88 | 33 |
| chr2:148730308\|148739650 | hsa_circ_0004491 | 34 | 24 | 8 | 17 | 24 | 2 | 32 | 47 | 24 | 326 | 148 | 56 |
| chr2:148730308\|148733544 | hsa_circ_0001074 | 101 | 90 | 16 | 61 | 100 | 43 | 20 | 28 | 17 | 519 | 252 | 121 |
| chr2:148653870\|148657467 | hsa_circ_0001073 | 15 | 12 | 9 | 15 | 14 | 5 | 17 | 28 | 7 | 183 | 85 | 31 |
| chr2:144966170\|144969146 | hsa_circ_0001072 | 44 | 40 | 18 | 44 | 45 | 29 | 37 | 52 | 28 | 461 | 228 | 62 |
| chr2:136505837\|136519481 | hsa_circ_0002981 | 18 | 13 | 9 | 13 | 16 | 8 | 17 | 27 | 16 | 149 | 81 | 21 |
| chr2:128944257\|128945188 | hsa_circ_0056439 | 25 | 14 | 14 | 9 | 22 | 7 | 20 | 40 | 11 | 158 | 63 | 26 |
| chr2:122514816\|122519100 | hsa_circ_0002847 | 37 | 25 | 14 | 20 | 29 | 13 | 29 | 31 | 31 | 338 | 135 | 40 |
| chr2:120684174\|120702816 | hsa_circ_0056248 | 38 | 26 | 13 | 21 | 7 | 5 | 25 | 20 | 16 | 225 | 126 | 38 |
| chr2:120684174\|120692534 | hsa_circ_0056247 | 40 | 29 | 11 | 22 | 33 | 13 | 32 | 46 | 17 | 289 | 166 | 42 |
| chr2:120677645\|120684242 | hsa_circ_0056244 | 11 | 4 | 2 | 3 | 3 | 9 | 6 | 5 | 4 | 47 | 16 | 5 |
| chr2:114697536\|114699936 | hsa_circ_0008712 | 12 | 9 | 5 | 3 | 4 | 3 | 5 | 13 | 14 | 119 | 37 | 34 |
| chr2:11426665\|11427862 | hsa_circ_0006526 | 8 | 8 | 2 | 5 | 8 | 12 | 8 | 5 | 3 | 173 | 40 | 13 |
| chr2:113057426\|113057606 | hsa_circ_0001062 | 100 | 105 | 51 | 28 | 20 | 23 | 10 | 7 | 7 | 224 | 79 | 5 |
| chr2:109388157\|109389502 | hsa_circ_0006965 | 25 | 15 | 8 | 8 | 9 | 8 | 22 | 16 | 25 | 180 | 85 | 19 |
| chr2:10740979\|10747437 | hsa_circ_0007344 | 5 | 4 | 4 | 6 | 3 | 3 | 14 | 9 | 3 | 91 | 48 | 12 |
| chr2:106774514\|106782539 | hsa_circ_0001060 | 131 | 93 | 45 | 84 | 99 | 51 | 39 | 33 | 40 | 1234 | 391 | 123 |
| chr2:106761646\|106782539 | hsa_circ_0004029 | 9 | 15 | 6 | 13 | 12 | 9 | 22 | 26 | 10 | 187 | 83 | 35 |
| chr2:101874253\|101881509 | hsa_circ_0001057 | 6 | 2 | 2 | 4 | 11 | 2 | 8 | 4 | 4 | 41 | 16 | 10 |
| chr2:101874253\|101879153 | hsa_circ_0002401 | 15 | 7 | 3 | 13 | 12 | 4 | 6 | 10 | 3 | 143 | 45 | 14 |
| chr2:100065798\|100081447 | hsa_circ_0007092 | 3 | 5 | 2 | 4 | 8 | 2 | 10 | 14 | 6 | 41 | 23 | 11 |
| chr19:8538548\|8539128 | hsa_circ_0049075 | 12 | 16 | 4 | 20 | 15 | 31 | 13 | 10 | 7 | 89 | 48 | 4 |
| chr19:8520289\|8528570 | hsa_circ_0006382 | 313 | 241 | 69 | 132 | 192 | 95 | 185 | 230 | 116 | 1896 | 708 | 299 |
| chr19:7184327\|7184648 | hsa_circ_0048965 | 6 | 6 | 7 | 8 | 12 | 7 | 18 | 25 | 7 | 76 | 51 | 13 |
| chr19:57967021\|57967550 | hsa_circ_0000963 | 47 | 53 | 25 | 54 | 79 | 30 | 16 | 17 | 14 | 446 | 234 | 39 |
| chr19:5653126\|5654467 | hsa_circ_0000881 | 16 | 16 | 7 | 6 | 10 | 6 | 18 | 29 | 14 | 133 | 76 | 23 |
| chr19:5604594\|5604947 | hsa_circ_0000880 | 10 | 7 | 2 | 8 | 6 | 3 | 9 | 10 | 11 | 78 | 59 | 11 |
| chr19:52785364\|52786666 | hsa_circ_0052095 | 22 | 14 | 5 | 9 | 10 | 6 | 20 | 18 | 19 | 130 | 64 | 35 |
| chr19:5047487\|5082515 | hsa_circ_0002926 | 31 | 41 | 9 | 40 | 39 | 13 | 55 | 92 | 47 | 354 | 312 | 90 |
| chr19:47767860\|47768203 | hsa_circ_0000944 | 25 | 30 | 4 | 15 | 28 | 14 | 27 | 37 | 26 | 118 | 87 | 19 |
| chr19:45766371\|45766625 | hsa_circ_0000940 | 8 | 6 | 5 | 3 | 6 | 5 | 7 | 7 | 2 | 52 | 32 | 7 |
| chr19:4418017\|4423871 | #N/A | 15 | 17 | 4 | 15 | 11 | 4 | 5 | 9 | 2 | 4 | 10 | 3 |
| chr19:41089304\|41089623 | hsa_circ_0000936 | 20 | 19 | 8 | 12 | 19 | 13 | 7 | 7 | 8 | 49 | 42 | 19 |
| chr19:39943996\|39944161 | hsa_circ_0000932 | 26 | 26 | 4 | 18 | 26 | 6 | 33 | 26 | 20 | 108 | 96 | 26 |
| chr19:37916770\|37917280 | hsa_circ_0004906 | 11 | 14 | 6 | 11 | 19 | 8 | 19 | 15 | 19 | 96 | 52 | 31 |
| chr19:3660964\|3661999 | hsa_circ_0000871 | 12 | 9 | 3 | 14 | 13 | 3 | 17 | 13 | 11 | 68 | 68 | 19 |
| chr19:33604673\|33605325 | hsa_circ_0008287 | 45 | 48 | 18 | 41 | 54 | 25 | 33 | 29 | 25 | 292 | 169 | 36 |
| chr19:30476130\|30477324 | hsa_circ_0000921 | 13 | 10 | 2 | 3 | 7 | 6 | 3 | 5 | 4 | 64 | 28 | 8 |
| chr19:30462100\|30477324 | hsa_circ_0050334 | 4 | 4 | 2 | 2 | 6 | 5 | 2 | 3 | 3 | 33 | 21 | 7 |
| chr19:2189731\|2194576 | #N/A | 7 | 15 | 6 | 11 | 10 | 3 | 18 | 31 | 15 | 103 | 91 | 34 |
| chr19:2180712\|2194576 | #N/A | 22 | 14 | 4 | 7 | 8 | 2 | 12 | 15 | 9 | 97 | 67 | 20 |
| chr19:21216262\|21216990 | hsa_circ_0008838 | 26 | 20 | 8 | 29 | 28 | 15 | 10 | 18 | 17 | 225 | 101 | 42 |
| chr19:19603115\|19603521 | hsa_circ_0006804 | 39 | 26 | 14 | 27 | 41 | 18 | 38 | 22 | 23 | 185 | 104 | 36 |
| chr19:18648411\|18649246 | hsa_circ_0000914 | 73 | 61 | 29 | 102 | 98 | 44 | 124 | 217 | 137 | 632 | 596 | 248 |
| chr19:18648411\|18649243 | hsa_circ_0050119 | 45 | 49 | 14 | 49 | 40 | 21 | 70 | 119 | 75 | 337 | 323 | 146 |
| chr19:17387304\|17387718 | hsa_circ_0003253 | 6 | 4 | 2 | 12 | 13 | 7 | 22 | 17 | 15 | 89 | 68 | 15 |
| chr19:17212470\|17213367 | hsa_circ_0000907 | 10 | 11 | 2 | 13 | 8 | 6 | 17 | 18 | 9 | 64 | 73 | 22 |
| chr19:13039156\|13039661 | hsa_circ_0000896 | 29 | 28 | 9 | 55 | 45 | 20 | 57 | 72 | 32 | 279 | 182 | 63 |
| chr19:11015627\|11019883 | hsa_circ_0004552 | 11 | 23 | 9 | 20 | 23 | 4 | 21 | 19 | 15 | 101 | 85 | 22 |
| chr19:10284547\|10288043 | hsa_circ_0006102 | 19 | 23 | 7 | 14 | 14 | 15 | 11 | 5 | 4 | 44 | 34 | 18 |
| chr19:10283766\|10288043 | hsa_circ_0000889 | 15 | 21 | 4 | 11 | 12 | 7 | 8 | 12 | 11 | 64 | 51 | 15 |
| chr19:10279006\|10288043 | hsa_circ_0049221 | 60 | 62 | 28 | 42 | 69 | 26 | 30 | 66 | 38 | 350 | 181 | 94 |
| chr19:10274002\|10288043 | #N/A | 58 | 60 | 28 | 23 | 36 | 26 | 30 | 52 | 28 | 322 | 125 | 54 |
| chr19:10274002\|10284581 | #N/A | 47 | 23 | 10 | 14 | 21 | 18 | 11 | 16 | 11 | 127 | 60 | 31 |
| chr19:10273343\|10288043 | #N/A | 13 | 9 | 2 | 7 | 6 | 6 | 5 | 7 | 3 | 69 | 18 | 8 |
| chr19:10273343\|10277361 | #N/A | 17 | 8 | 4 | 12 | 17 | 15 | 14 | 9 | 7 | 77 | 38 | 18 |
| chr18:9931807\|9937063 | hsa_circ_0006990 | 55 | 47 | 22 | 38 | 40 | 29 | 46 | 54 | 30 | 561 | 223 | 78 |
| chr18:9583115\|9595151 | hsa_circ_0007509 | 7 | 8 | 2 | 5 | 2 | 3 | 4 | 4 | 2 | 43 | 27 | 11 |
| chr18:9583115\|9595100 | hsa_circ_0009022 | 16 | 10 | 4 | 4 | 9 | 4 | 10 | 10 | 5 | 108 | 79 | 13 |
| chr18:9524592\|9525849 | hsa_circ_0005158 | 66 | 75 | 21 | 55 | 78 | 20 | 83 | 97 | 69 | 841 | 325 | 125 |
| chr18:9182380\|9221997 | hsa_circ_0000826 | 65 | 54 | 27 | 16 | 19 | 15 | 19 | 6 | 4 | 183 | 61 | 26 |
| chr18:77668146\|77668309 | #N/A | 35 | 11 | 20 | 18 | 20 | 15 | 12 | 8 | 8 | 253 | 139 | 23 |
| chr18:77488907\|77496521 | hsa_circ_0008435 | 13 | 6 | 4 | 14 | 19 | 4 | 15 | 20 | 18 | 63 | 47 | 19 |
| chr18:76953183\|76974038 | hsa_circ_0003275 | 14 | 15 | 4 | 15 | 11 | 4 | 3 | 11 | 7 | 105 | 58 | 9 |
| chr18:76886267\|76914555 | hsa_circ_0007134 | 14 | 19 | 8 | 15 | 15 | 9 | 14 | 11 | 11 | 107 | 85 | 17 |
| chr18:756557\|756835 | hsa_circ_0046702 | 8 | 3 | 2 | 8 | 9 | 5 | 4 | 10 | 3 | 55 | 32 | 16 |
| chr18:74561482\|74563895 | hsa_circ_0004979 | 72 | 69 | 20 | 91 | 96 | 57 | 93 | 91 | 57 | 773 | 365 | 70 |
| chr18:60206914\|60217693 | hsa_circ_0000854 | 136 | 110 | 60 | 101 | 86 | 35 | 131 | 159 | 91 | 1130 | 586 | 219 |
| chr18:55833020\|55919286 | #N/A | 29 | 25 | 8 | 13 | 11 | 8 | 11 | 14 | 10 | 189 | 68 | 36 |
| chr18:55278869\|55283207 | hsa_circ_0047769 | 35 | 28 | 8 | 24 | 15 | 14 | 28 | 49 | 24 | 218 | 84 | 29 |
| chr18:55238624\|55247431 | #N/A | 4 | 10 | 4 | 8 | 10 | 3 | 11 | 28 | 13 | 72 | 37 | 19 |
| chr18:55233679\|55240597 | #N/A | 5 | 2 | 3 | 3 | 4 | 8 | 11 | 9 | 5 | 38 | 20 | 5 |
| chr18:51797730\|51800460 | hsa_circ_0007180 | 12 | 4 | 3 | 5 | 6 | 2 | 6 | 11 | 5 | 82 | 27 | 9 |
| chr18:47017996\|47018203 | hsa_circ_0007956 | 20 | 14 | 13 | 13 | 19 | 13 | 11 | 16 | 5 | 174 | 96 | 24 |
| chr18:45391430\|45423180 | hsa_circ_0000847 | 40 | 19 | 18 | 17 | 31 | 12 | 25 | 21 | 10 | 188 | 118 | 44 |
| chr18:45391430\|45396935 | hsa_circ_0003694 | 37 | 13 | 3 | 26 | 17 | 14 | 13 | 25 | 12 | 227 | 122 | 49 |
| chr18:44470543\|44483598 | #N/A | 15 | 15 | 4 | 12 | 13 | 6 | 22 | 25 | 15 | 152 | 49 | 35 |
| chr18:43705710\|43708803 | #N/A | 16 | 8 | 6 | 8 | 7 | 6 | 3 | 8 | 9 | 37 | 21 | 12 |
| chr18:43698147\|43700026 | hsa_circ_0047581 | 6 | 3 | 3 | 6 | 9 | 2 | 4 | 7 | 5 | 50 | 28 | 6 |
| chr18:43568668\|43572168 | #N/A | 5 | 3 | 4 | 5 | 6 | 2 | 3 | 6 | 4 | 42 | 22 | 12 |
| chr18:43319128\|43319627 | #N/A | 20 | 22 | 5 | 15 | 23 | 7 | 11 | 10 | 13 | 192 | 105 | 47 |
| chr18:39623697\|39629569 | hsa_circ_0006527 | 35 | 17 | 11 | 19 | 20 | 7 | 11 | 10 | 7 | 327 | 165 | 27 |
| chr18:39607407\|39629569 | hsa_circ_0007765 | 41 | 23 | 15 | 8 | 16 | 3 | 27 | 34 | 21 | 208 | 157 | 41 |
| chr18:39595440\|39609405 | hsa_circ_0002560 | 11 | 11 | 4 | 4 | 10 | 5 | 10 | 12 | 3 | 74 | 48 | 11 |
| chr18:33606863\|33613800 | hsa_circ_0000842 | 17 | 11 | 4 | 10 | 15 | 5 | 2 | 3 | 8 | 119 | 44 | 29 |
| chr18:29691717\|29693823 | hsa_circ_0005729 | 15 | 21 | 2 | 13 | 9 | 2 | 10 | 8 | 7 | 72 | 34 | 8 |
| chr18:29412047\|29419420 | hsa_circ_0003805 | 27 | 27 | 9 | 11 | 22 | 7 | 9 | 4 | 6 | 154 | 83 | 26 |
| chr18:23895193\|23915195 | hsa_circ_0047322 | 11 | 5 | 2 | 2 | 3 | 3 | 5 | 3 | 3 | 46 | 36 | 11 |
| chr18:21644104\|21649235 | hsa_circ_0000839 | 13 | 23 | 14 | 14 | 24 | 8 | 13 | 8 | 8 | 201 | 87 | 29 |
| chr18:20516717\|20529676 | #N/A | 35 | 32 | 11 | 11 | 14 | 11 | 18 | 18 | 13 | 192 | 79 | 25 |
| chr18:196637\|204692 | hsa_circ_0005653 | 13 | 5 | 3 | 4 | 7 | 4 | 9 | 8 | 2 | 92 | 27 | 6 |
| chr18:19345733\|19359646 | hsa_circ_0000835 | 31 | 7 | 8 | 6 | 9 | 6 | 29 | 23 | 20 | 203 | 113 | 50 |
| chr18:13099475\|13103587 | hsa_circ_0005863 | 9 | 10 | 9 | 14 | 18 | 6 | 9 | 18 | 2 | 162 | 74 | 15 |
| chr18:12999420\|13019205 | hsa_circ_0000831 | 21 | 13 | 6 | 7 | 14 | 6 | 13 | 13 | 8 | 128 | 60 | 20 |
| chr17:80521230\|80526077 | hsa_circ_0000816 | 17 | 17 | 6 | 20 | 21 | 9 | 16 | 21 | 6 | 130 | 88 | 31 |
| chr17:79571574\|79575848 | hsa_circ_0000814 | 10 | 12 | 5 | 12 | 17 | 6 | 16 | 24 | 13 | 164 | 84 | 44 |
| chr17:76082584\|76083174 | hsa_circ_0045862 | 9 | 8 | 2 | 17 | 18 | 6 | 11 | 14 | 6 | 45 | 25 | 7 |
| chr17:76000251\|76027923 | hsa_circ_0008622 | 22 | 18 | 11 | 14 | 21 | 7 | 23 | 30 | 16 | 170 | 89 | 33 |
| chr17:73238417\|73239651 | hsa_circ_0003684 | 21 | 9 | 4 | 12 | 6 | 9 | 13 | 26 | 12 | 108 | 66 | 17 |
| chr17:65941525\|65972074 | hsa_circ_0000799 | 52 | 36 | 16 | 25 | 13 | 11 | 3 | 3 | 2 | 34 | 18 | 5 |
| chr17:65941525\|65944422 | hsa_circ_0000798 | 27 | 46 | 12 | 28 | 34 | 17 | 17 | 13 | 8 | 71 | 64 | 15 |
| chr17:65924471\|65928135 | hsa_circ_0045462 | 21 | 25 | 6 | 10 | 9 | 5 | 12 | 16 | 13 | 102 | 56 | 33 |
| chr17:65916131\|65919106 | hsa_circ_0003796 | 15 | 12 | 3 | 6 | 15 | 5 | 8 | 7 | 4 | 138 | 51 | 29 |
| chr17:65887960\|65890281 | hsa_circ_0005347 | 17 | 7 | 2 | 8 | 4 | 4 | 4 | 8 | 8 | 83 | 27 | 6 |
| chr17:61841376\|61842207 | hsa_circ_0009168 | 21 | 19 | 7 | 16 | 21 | 6 | 11 | 16 | 10 | 186 | 58 | 24 |
| chr17:60111148\|60112969 | hsa_circ_0004273 | 117 | 94 | 33 | 97 | 81 | 46 | 84 | 91 | 68 | 1084 | 418 | 125 |
| chr17:60106902\|60112969 | hsa_circ_0045096 | 20 | 21 | 11 | 5 | 13 | 4 | 9 | 10 | 8 | 127 | 59 | 25 |
| chr17:60061532\|60062451 | hsa_circ_0002220 | 57 | 54 | 23 | 37 | 39 | 21 | 42 | 48 | 26 | 574 | 217 | 68 |
| chr17:60059559\|60060475 | #N/A | 13 | 7 | 3 | 6 | 10 | 3 | 15 | 5 | 9 | 66 | 16 | 10 |
| chr17:59853762\|59857762 | hsa_circ_0006968 | 31 | 14 | 4 | 8 | 9 | 8 | 16 | 18 | 7 | 145 | 58 | 28 |
| chr17:59821793\|59857762 | hsa_circ_0000794 | 15 | 14 | 3 | 11 | 9 | 6 | 15 | 22 | 17 | 206 | 72 | 32 |
| chr17:58346811\|58348842 | hsa_circ_0007272 | 53 | 39 | 12 | 36 | 40 | 36 | 44 | 36 | 32 | 331 | 180 | 53 |
| chr17:58342773\|58372162 | hsa_circ_0044949 | 8 | 14 | 4 | 4 | 6 | 2 | 9 | 8 | 6 | 107 | 37 | 18 |
| chr17:58342773\|58348842 | hsa_circ_0000793 | 35 | 34 | 12 | 21 | 21 | 12 | 40 | 40 | 36 | 291 | 123 | 39 |
| chr17:58012554\|58013902 | #N/A | 4 | 4 | 2 | 2 | 3 | 5 | 6 | 2 | 3 | 20 | 14 | 5 |
| chr17:57987923\|57992064 | #N/A | 5 | 5 | 3 | 9 | 7 | 3 | 10 | 11 | 2 | 32 | 29 | 5 |
| chr17:57808782\|57816308 | hsa_circ_0006508 | 40 | 37 | 10 | 36 | 29 | 8 | 45 | 50 | 23 | 411 | 158 | 55 |
| chr17:57430576\|57430887 | hsa_circ_0005600 | 48 | 33 | 18 | 65 | 62 | 27 | 75 | 71 | 38 | 357 | 239 | 83 |
| chr17:56780557\|56787351 | #N/A | 15 | 16 | 3 | 7 | 8 | 6 | 8 | 8 | 3 | 129 | 51 | 14 |
| chr17:56293449\|56294097 | hsa_circ_0044767 | 9 | 5 | 4 | 4 | 5 | 8 | 5 | 6 | 6 | 33 | 29 | 7 |
| chr17:53478830\|53481229 | hsa_circ_0002015 | 9 | 20 | 7 | 13 | 23 | 3 | 7 | 6 | 4 | 105 | 60 | 16 |
| chr17:53108529\|53124515 | #N/A | 8 | 7 | 2 | 5 | 4 | 3 | 12 | 7 | 8 | 78 | 26 | 10 |
| chr17:48814321\|48819092 | #N/A | 5 | 11 | 3 | 7 | 9 | 5 | 3 | 8 | 6 | 72 | 37 | 18 |
| chr17:47388674\|47390207 | hsa_circ_0000782 | 11 | 5 | 3 | 8 | 6 | 3 | 5 | 16 | 7 | 119 | 52 | 19 |
| chr17:47388674\|47389404 | hsa_circ_0003258 | 28 | 33 | 9 | 35 | 29 | 21 | 11 | 24 | 16 | 244 | 131 | 28 |
| chr17:45479498\|45492285 | hsa_circ_0000778 | 27 | 6 | 5 | 6 | 6 | 6 | 13 | 14 | 8 | 152 | 50 | 25 |
| chr17:45247283\|45249430 | hsa_circ_0044234 | 29 | 29 | 5 | 15 | 23 | 15 | 23 | 24 | 18 | 299 | 121 | 61 |
| chr17:45232038\|45235669 | hsa_circ_0002502 | 24 | 31 | 9 | 9 | 12 | 10 | 13 | 10 | 6 | 130 | 78 | 13 |
| chr17:44248221\|44249598 | hsa_circ_0005455 | 16 | 22 | 5 | 12 | 6 | 7 | 20 | 8 | 15 | 95 | 69 | 12 |
| chr17:44197334\|44198870 | #N/A | 6 | 10 | 8 | 3 | 8 | 6 | 11 | 7 | 10 | 87 | 38 | 7 |
| chr17:43552466\|43553092 | hsa_circ_0044177 | 23 | 48 | 15 | 26 | 52 | 11 | 57 | 59 | 42 | 257 | 251 | 72 |
| chr17:4186093\|4210418 | hsa_circ_0004805 | 12 | 12 | 3 | 5 | 5 | 8 | 16 | 11 | 7 | 147 | 45 | 19 |
| chr17:4186093\|4200109 | hsa_circ_0003239 | 41 | 31 | 9 | 24 | 41 | 13 | 53 | 62 | 27 | 513 | 231 | 60 |
| chr17:41247863\|41276132 | hsa_circ_0008673 | 7 | 6 | 4 | 6 | 6 | 6 | 10 | 5 | 5 | 84 | 37 | 10 |
| chr17:41215350\|41215968 | #N/A | 17 | 9 | 3 | 10 | 19 | 11 | 17 | 9 | 7 | 239 | 86 | 18 |
| chr17:40879653\|40882936 | hsa_circ_0043898 | 42 | 44 | 7 | 33 | 36 | 25 | 40 | 51 | 23 | 418 | 160 | 57 |
| chr17:40652725\|40653322 | hsa_circ_0008179 | 54 | 53 | 23 | 51 | 47 | 34 | 60 | 58 | 17 | 762 | 316 | 99 |
| chr17:40650942\|40653322 | hsa_circ_0043837 | 97 | 84 | 31 | 66 | 66 | 46 | 85 | 93 | 50 | 802 | 417 | 145 |
| chr17:38551701\|38552717 | hsa_circ_0005877 | 18 | 12 | 9 | 13 | 18 | 15 | 14 | 32 | 12 | 127 | 72 | 17 |
| chr17:38547758\|38548989 | #N/A | 29 | 35 | 12 | 15 | 24 | 13 | 16 | 26 | 13 | 228 | 91 | 29 |
| chr17:37866066\|37872192 | #N/A | 7 | 11 | 8 | 9 | 12 | 2 | 15 | 37 | 17 | 107 | 100 | 53 |
| chr17:37579580\|37580991 | hsa_circ_0043427 | 11 | 14 | 2 | 15 | 24 | 9 | 13 | 11 | 5 | 138 | 81 | 19 |
| chr17:3717616\|3719564 | hsa_circ_0041462 | 19 | 15 | 4 | 7 | 8 | 4 | 16 | 26 | 13 | 179 | 137 | 20 |
| chr17:35800606\|35822225 | hsa_circ_0043283 | 12 | 10 | 3 | 8 | 7 | 6 | 10 | 9 | 4 | 84 | 23 | 12 |
| chr17:30689933\|30695033 | hsa_circ_0005565 | 9 | 5 | 3 | 15 | 11 | 5 | 5 | 7 | 6 | 52 | 33 | 6 |
| chr17:30498062\|30503232 | hsa_circ_0008604 | 50 | 37 | 21 | 37 | 26 | 28 | 29 | 37 | 21 | 337 | 120 | 42 |
| chr17:29170931\|29171934 | hsa_circ_0042867 | 26 | 11 | 7 | 23 | 17 | 14 | 8 | 6 | 4 | 104 | 35 | 19 |
| chr17:28811232\|28819771 | hsa_circ_0007460 | 18 | 22 | 13 | 20 | 26 | 9 | 40 | 45 | 22 | 358 | 146 | 49 |
| chr17:28808161\|28819771 | hsa_circ_0042839 | 17 | 12 | 3 | 11 | 12 | 6 | 17 | 17 | 11 | 158 | 52 | 34 |
| chr17:28808161\|28817235 | hsa_circ_0005194 | 2 | 7 | 4 | 4 | 5 | 4 | 9 | 6 | 2 | 47 | 30 | 8 |
| chr17:28808161\|28811788 | hsa_circ_0002167 | 10 | 14 | 6 | 26 | 17 | 19 | 30 | 22 | 10 | 139 | 59 | 20 |
| chr17:28011581\|28030080 | hsa_circ_0000754 | 11 | 22 | 7 | 9 | 14 | 6 | 18 | 21 | 12 | 96 | 51 | 22 |
| chr17:27809215\|27818883 | hsa_circ_0007882 | 13 | 9 | 8 | 14 | 6 | 6 | 12 | 8 | 6 | 95 | 27 | 6 |
| chr17:27778473\|27778698 | hsa_circ_0002839 | 14 | 17 | 2 | 11 | 10 | 8 | 15 | 9 | 7 | 80 | 59 | 12 |
| chr17:26490569\|26499644 | hsa_circ_0003638 | 24 | 25 | 7 | 19 | 31 | 11 | 34 | 48 | 21 | 260 | 170 | 38 |
| chr17:20107646\|20109225 | hsa_circ_0000745 | 834 | 760 | 347 | 232 | 253 | 127 | 189 | 187 | 109 | 2149 | 1001 | 263 |
| chr17:19921300\|20013875 | #N/A | 10 | 15 | 6 | 13 | 10 | 7 | 34 | 22 | 11 | 90 | 48 | 39 |
| chr17:19861327\|19861884 | hsa_circ_0004719 | 4 | 9 | 3 | 5 | 4 | 3 | 13 | 17 | 7 | 69 | 23 | 12 |
| chr17:18768782\|18769265 | hsa_circ_0002278 | 15 | 14 | 4 | 14 | 7 | 11 | 11 | 5 | 5 | 93 | 43 | 12 |
| chr17:1746097\|1756483 | hsa_circ_0000734 | 31 | 29 | 14 | 17 | 24 | 6 | 46 | 58 | 30 | 253 | 123 | 36 |
| chr17:1746097\|1747980 | hsa_circ_0005946 | 36 | 20 | 14 | 23 | 37 | 20 | 35 | 16 | 14 | 104 | 94 | 23 |
| chr17:16042322\|16047010 | #N/A | 3 | 4 | 2 | 4 | 3 | 5 | 4 | 4 | 6 | 38 | 24 | 8 |
| chr17:16004564\|16005119 | hsa_circ_0042174 | 24 | 33 | 9 | 18 | 30 | 7 | 27 | 63 | 23 | 159 | 158 | 32 |
| chr17:1264386\|1265302 | hsa_circ_0007643 | 17 | 17 | 4 | 10 | 17 | 11 | 19 | 20 | 3 | 172 | 81 | 27 |
| chr16:9009111\|9011013 | hsa_circ_0005152 | 61 | 31 | 18 | 21 | 17 | 12 | 18 | 20 | 16 | 253 | 96 | 31 |
| chr16:90045218\|90046744 | #N/A | 15 | 14 | 4 | 14 | 13 | 6 | 17 | 9 | 8 | 86 | 35 | 14 |
| chr16:8952207\|8953192 | hsa_circ_0000669 | 13 | 15 | 6 | 25 | 41 | 18 | 30 | 27 | 14 | 110 | 63 | 29 |
| chr16:88675342\|88677944 | hsa_circ_0004640 | 9 | 8 | 2 | 14 | 2 | 5 | 9 | 17 | 17 | 44 | 19 | 3 |
| chr16:88066715\|88071617 | hsa_circ_0040827 | 9 | 12 | 2 | 10 | 4 | 5 | 2 | 9 | 9 | 65 | 46 | 21 |
| chr16:88061089\|88071617 | hsa_circ_0040823 | 32 | 25 | 10 | 36 | 47 | 24 | 66 | 73 | 52 | 304 | 322 | 136 |
| chr16:88008654\|88017865 | hsa_circ_0040809 | 20 | 19 | 5 | 11 | 19 | 10 | 37 | 43 | 25 | 207 | 148 | 47 |
| chr16:85667520\|85667738 | hsa_circ_0000722 | 956 | 1118 | 242 | 2085 | 2104 | 1244 | 864 | 712 | 667 | 2378 | 2549 | 817 |
| chr16:81058320\|81060243 | hsa_circ_0007405 | 5 | 4 | 4 | 5 | 9 | 5 | 3 | 3 | 4 | 67 | 23 | 3 |
| chr16:80718435\|80719026 | hsa_circ_0004087 | 8 | 4 | 5 | 7 | 2 | 2 | 5 | 10 | 9 | 50 | 38 | 11 |
| chr16:74982417\|74990508 | hsa_circ_0040533 | 5 | 9 | 2 | 16 | 6 | 4 | 7 | 17 | 5 | 55 | 43 | 10 |
| chr16:74493580\|74497377 | hsa_circ_0003315 | 11 | 8 | 3 | 5 | 9 | 3 | 8 | 17 | 7 | 83 | 73 | 17 |
| chr16:72122886\|72124685 | hsa_circ_0040414 | 46 | 36 | 15 | 31 | 40 | 16 | 59 | 78 | 36 | 470 | 212 | 94 |
| chr16:71748415\|71748704 | hsa_circ_0040364 | 15 | 4 | 5 | 14 | 10 | 5 | 14 | 10 | 5 | 87 | 52 | 16 |
| chr16:71712658\|71715808 | hsa_circ_0000715 | 47 | 40 | 14 | 26 | 27 | 12 | 54 | 47 | 18 | 319 | 186 | 59 |
| chr16:71710350\|71713438 | hsa_circ_0040356 | 23 | 18 | 7 | 8 | 15 | 9 | 30 | 26 | 21 | 174 | 95 | 35 |
| chr16:71692136\|71713438 | hsa_circ_0005638 | 17 | 15 | 5 | 2 | 3 | 3 | 5 | 4 | 5 | 67 | 31 | 6 |
| chr16:70598968\|70601439 | hsa_circ_0009163 | 11 | 7 | 5 | 12 | 10 | 4 | 17 | 22 | 16 | 74 | 56 | 20 |
| chr16:70294947\|70296427 | hsa_circ_0003438 | 18 | 16 | 5 | 13 | 13 | 6 | 28 | 26 | 25 | 143 | 89 | 19 |
| chr16:70076254\|70076906 | hsa_circ_0040123 | 12 | 12 | 3 | 5 | 16 | 5 | 19 | 21 | 12 | 111 | 63 | 15 |
| chr16:69404386\|69406258 | #N/A | 89 | 101 | 42 | 84 | 78 | 47 | 37 | 66 | 43 | 541 | 286 | 136 |
| chr16:68155890\|68160513 | hsa_circ_0000711 | 57 | 48 | 13 | 62 | 63 | 28 | 32 | 25 | 13 | 234 | 117 | 28 |
| chr16:68155890\|68157024 | hsa_circ_0005615 | 30 | 27 | 12 | 45 | 45 | 26 | 13 | 21 | 16 | 158 | 105 | 27 |
| chr16:67662273\|67663436 | hsa_circ_0008272 | 85 | 58 | 21 | 58 | 55 | 35 | 70 | 124 | 66 | 671 | 392 | 130 |
| chr16:58594116\|58594266 | hsa_circ_0007079 | 62 | 38 | 13 | 69 | 70 | 30 | 17 | 7 | 2 | 596 | 264 | 28 |
| chr16:58593708\|58594266 | hsa_circ_0000705 | 28 | 23 | 7 | 28 | 27 | 13 | 16 | 25 | 16 | 215 | 122 | 32 |
| chr16:57197913\|57207781 | hsa_circ_0006886 | 43 | 33 | 19 | 18 | 20 | 8 | 28 | 23 | 12 | 244 | 127 | 37 |
| chr16:56419831\|56423287 | hsa_circ_0000704 | 19 | 13 | 11 | 17 | 11 | 10 | 14 | 23 | 3 | 122 | 82 | 18 |
| chr16:53472928\|53481018 | hsa_circ_0000703 | 8 | 5 | 2 | 3 | 11 | 5 | 3 | 6 | 7 | 81 | 21 | 12 |
| chr16:47531310\|47549512 | hsa_circ_0000698 | 16 | 13 | 6 | 15 | 7 | 2 | 10 | 22 | 3 | 108 | 58 | 15 |
| chr16:3900298\|3901010 | hsa_circ_0007637 | 26 | 18 | 10 | 15 | 28 | 14 | 35 | 61 | 23 | 173 | 165 | 52 |
| chr16:354304\|364683 | hsa_circ_0037158 | 3 | 8 | 2 | 2 | 6 | 4 | 6 | 14 | 6 | 44 | 40 | 17 |
| chr16:31733947\|31734674 | hsa_circ_0007059 | 29 | 14 | 4 | 9 | 16 | 4 | 13 | 30 | 8 | 119 | 64 | 45 |
| chr16:31102096\|31102663 | hsa_circ_0006719 | 29 | 11 | 5 | 11 | 14 | 8 | 12 | 19 | 10 | 98 | 81 | 30 |
| chr16:30740287\|30740893 | hsa_circ_0006127 | 26 | 18 | 9 | 30 | 36 | 16 | 21 | 65 | 38 | 210 | 185 | 65 |
| chr16:30507418\|30507887 | #N/A | 10 | 9 | 3 | 7 | 5 | 4 | 11 | 25 | 14 | 137 | 67 | 27 |
| chr16:30495148\|30495584 | hsa_circ_0000690 | 18 | 20 | 8 | 31 | 37 | 9 | 25 | 48 | 19 | 253 | 133 | 38 |
| chr16:24762047\|24776032 | #N/A | 8 | 8 | 8 | 8 | 13 | 5 | 5 | 5 | 3 | 97 | 55 | 9 |
| chr16:24762047\|24769681 | #N/A | 52 | 48 | 35 | 60 | 50 | 44 | 62 | 47 | 23 | 764 | 358 | 55 |
| chr16:23999829\|24046868 | hsa_circ_0000681 | 14 | 11 | 3 | 16 | 13 | 4 | 32 | 27 | 9 | 78 | 86 | 23 |
| chr16:22360600\|22361170 | hsa_circ_0004682 | 10 | 14 | 2 | 4 | 20 | 10 | 12 | 17 | 10 | 92 | 57 | 22 |
| chr16:18852887\|18856973 | hsa_circ_0006434 | 16 | 18 | 2 | 9 | 9 | 4 | 19 | 18 | 11 | 80 | 52 | 18 |
| chr16:18809247\|18810156 | hsa_circ_0004833 | 32 | 37 | 15 | 36 | 46 | 18 | 41 | 22 | 15 | 357 | 183 | 70 |
| chr16:1859239\|1859834 | #N/A | 230 | 201 | 75 | 198 | 223 | 135 | 199 | 165 | 135 | 976 | 817 | 246 |
| chr16:1682213\|1691188 | hsa_circ_0037322 | 3 | 2 | 3 | 5 | 5 | 3 | 5 | 6 | 2 | 28 | 18 | 7 |
| chr16:1675974\|1691188 | hsa_circ_0003592 | 8 | 3 | 8 | 4 | 8 | 3 | 11 | 23 | 6 | 73 | 62 | 23 |
| chr16:1675974\|1682366 | hsa_circ_0005606 | 4 | 9 | 2 | 10 | 4 | 8 | 14 | 29 | 10 | 64 | 56 | 17 |
| chr16:15973661\|15978062 | hsa_circ_0003349 | 23 | 29 | 7 | 11 | 8 | 9 | 12 | 12 | 5 | 254 | 80 | 28 |
| chr16:15758593\|15761296 | hsa_circ_0038054 | 20 | 17 | 3 | 13 | 9 | 6 | 19 | 19 | 13 | 120 | 62 | 27 |
| chr16:15091610\|15092262 | hsa_circ_0038005 | 51 | 35 | 17 | 59 | 38 | 33 | 40 | 26 | 26 | 310 | 147 | 35 |
| chr16:148143\|150507 | hsa_circ_0037130 | 49 | 66 | 19 | 76 | 61 | 23 | 48 | 87 | 50 | 281 | 249 | 82 |
| chr16:14693761\|14698083 | hsa_circ_0037972 | 13 | 11 | 4 | 6 | 12 | 8 | 7 | 6 | 4 | 109 | 62 | 14 |
| chr16:14687158\|14698083 | hsa_circ_0005627 | 5 | 5 | 4 | 9 | 4 | 2 | 10 | 2 | 4 | 39 | 24 | 6 |
| chr16:11214472\|11220003 | hsa_circ_0002988 | 13 | 14 | 2 | 28 | 18 | 7 | 16 | 22 | 14 | 54 | 52 | 11 |
| chr16:11114050\|11220003 | hsa_circ_0007846 | 30 | 29 | 11 | 30 | 36 | 17 | 35 | 22 | 22 | 103 | 69 | 21 |
| chr16:11114050\|11154879 | hsa_circ_0000672 | 43 | 37 | 13 | 45 | 40 | 21 | 40 | 55 | 33 | 277 | 194 | 89 |
| chr16:11114050\|11145498 | hsa_circ_0002086 | 20 | 16 | 10 | 3 | 8 | 3 | 16 | 34 | 12 | 107 | 60 | 23 |
| chr15:93543742\|93558139 | hsa_circ_0037002 | 18 | 16 | 6 | 9 | 8 | 2 | 12 | 5 | 4 | 72 | 32 | 11 |
| chr15:93543742\|93552553 | hsa_circ_0037000 | 30 | 30 | 17 | 7 | 8 | 6 | 3 | 6 | 7 | 67 | 20 | 6 |
| chr15:93540187\|93541851 | hsa_circ_0000655 | 29 | 27 | 7 | 26 | 33 | 15 | 48 | 53 | 27 | 306 | 165 | 45 |
| chr15:93496587\|93499879 | hsa_circ_0036984 | 4 | 8 | 8 | 7 | 5 | 4 | 13 | 16 | 4 | 111 | 60 | 20 |
| chr15:93467551\|93472321 | hsa_circ_0007262 | 30 | 56 | 4 | 29 | 41 | 27 | 77 | 97 | 42 | 400 | 253 | 96 |
| chr15:90984738\|90986710 | hsa_circ_0000652 | 7 | 7 | 5 | 7 | 14 | 5 | 9 | 13 | 3 | 79 | 30 | 13 |
| chr15:90760671\|90764997 | hsa_circ_0000650 | 14 | 9 | 4 | 10 | 11 | 5 | 18 | 21 | 14 | 51 | 71 | 20 |
| chr15:90760671\|90763123 | hsa_circ_0036763 | 5 | 9 | 3 | 5 | 5 | 4 | 16 | 8 | 5 | 62 | 47 | 15 |
| chr15:89656956\|89659752 | hsa_circ_0007099 | 74 | 89 | 39 | 114 | 121 | 54 | 132 | 108 | 59 | 828 | 439 | 152 |
| chr15:85230856\|85234875 | hsa_circ_0036599 | 7 | 9 | 2 | 3 | 6 | 8 | 2 | 6 | 2 | 51 | 12 | 14 |
| chr15:85223944\|85234875 | hsa_circ_0000647 | 20 | 15 | 6 | 13 | 8 | 8 | 25 | 45 | 18 | 260 | 103 | 23 |
| chr15:80412670\|80415142 | hsa_circ_0000643 | 303 | 261 | 98 | 224 | 237 | 75 | 305 | 328 | 190 | 2683 | 1371 | 364 |
| chr15:80390758\|80415142 | hsa_circ_0000642 | 34 | 31 | 11 | 11 | 22 | 6 | 29 | 39 | 16 | 258 | 124 | 32 |
| chr15:77657505\|77681144 | #N/A | 26 | 19 | 9 | 18 | 7 | 7 | 5 | 10 | 3 | 140 | 22 | 9 |
| chr15:76152219\|76165909 | #N/A | 60 | 38 | 17 | 24 | 28 | 9 | 37 | 35 | 25 | 299 | 100 | 29 |
| chr15:72810408\|72853890 | hsa_circ_0000629 | 6 | 3 | 3 | 2 | 8 | 3 | 7 | 20 | 2 | 60 | 42 | 3 |
| chr15:72810408\|72848248 | hsa_circ_0000628 | 9 | 5 | 3 | 3 | 4 | 5 | 8 | 19 | 4 | 88 | 34 | 12 |
| chr15:68434284\|68446033 | hsa_circ_0003916 | 14 | 11 | 7 | 9 | 3 | 5 | 13 | 15 | 9 | 126 | 57 | 23 |
| chr15:68434284\|68439038 | hsa_circ_0008378 | 16 | 6 | 4 | 8 | 8 | 3 | 27 | 12 | 9 | 122 | 34 | 22 |
| chr15:66828271\|66839043 | hsa_circ_0008168 | 26 | 17 | 8 | 10 | 7 | 7 | 9 | 22 | 10 | 214 | 96 | 22 |
| chr15:66048478\|66053776 | #N/A | 20 | 21 | 3 | 11 | 17 | 2 | 14 | 19 | 22 | 163 | 77 | 26 |
| chr15:66044717\|66053776 | hsa_circ_0035957 | 77 | 54 | 17 | 32 | 32 | 15 | 37 | 57 | 29 | 553 | 217 | 82 |
| chr15:66044717\|66048810 | hsa_circ_0035956 | 30 | 22 | 5 | 11 | 17 | 10 | 10 | 25 | 7 | 144 | 51 | 23 |
| chr15:66021410\|66031213 | hsa_circ_0035944 | 47 | 30 | 10 | 25 | 15 | 9 | 23 | 28 | 6 | 287 | 94 | 29 |
| chr15:66021410\|66025156 | hsa_circ_0035943 | 19 | 18 | 4 | 10 | 16 | 11 | 28 | 20 | 11 | 160 | 133 | 38 |
| chr15:66015186\|66025156 | #N/A | 21 | 19 | 12 | 9 | 10 | 5 | 29 | 19 | 7 | 194 | 105 | 46 |
| chr15:65994643\|65998562 | hsa_circ_0035935 | 10 | 6 | 3 | 3 | 11 | 7 | 7 | 13 | 2 | 97 | 54 | 14 |
| chr15:65994643\|65995346 | hsa_circ_0007072 | 71 | 56 | 17 | 40 | 34 | 17 | 70 | 96 | 42 | 696 | 279 | 95 |
| chr15:65471272\|65472542 | hsa_circ_0004374 | 55 | 49 | 26 | 70 | 73 | 17 | 73 | 94 | 41 | 599 | 228 | 105 |
| chr15:65266940\|65275931 | hsa_circ_0003526 | 5 | 5 | 6 | 4 | 5 | 4 | 12 | 19 | 8 | 92 | 53 | 2 |
| chr15:64791492\|64792365 | hsa_circ_0000615 | 60 | 84 | 28 | 31 | 55 | 34 | 66 | 137 | 66 | 428 | 266 | 108 |
| chr15:64404772\|64415745 | hsa_circ_0002178 | 12 | 12 | 5 | 14 | 6 | 4 | 12 | 20 | 15 | 149 | 70 | 12 |
| chr15:64404772\|64411053 | hsa_circ_0003867 | 9 | 7 | 4 | 3 | 9 | 3 | 3 | 13 | 8 | 50 | 39 | 18 |
| chr15:64066893\|64067848 | hsa_circ_0007112 | 13 | 15 | 5 | 4 | 10 | 4 | 5 | 17 | 13 | 78 | 37 | 13 |
| chr15:64004974\|64008672 | hsa_circ_0035803 | 20 | 11 | 3 | 15 | 10 | 10 | 11 | 19 | 9 | 104 | 64 | 13 |
| chr15:63998979\|64008672 | hsa_circ_0035801 | 41 | 38 | 11 | 16 | 32 | 11 | 32 | 73 | 25 | 251 | 149 | 50 |
| chr15:63988323\|64008672 | hsa_circ_0035796 | 77 | 89 | 28 | 44 | 42 | 23 | 66 | 97 | 35 | 419 | 260 | 100 |
| chr15:63982740\|63987093 | #N/A | 3 | 3 | 2 | 3 | 2 | 3 | 4 | 3 | 2 | 30 | 9 | 5 |
| chr15:63845914\|63855207 | hsa_circ_0008153 | 13 | 4 | 7 | 9 | 9 | 4 | 8 | 12 | 15 | 162 | 71 | 35 |
| chr15:62828792\|62833481 | #N/A | 8 | 11 | 3 | 8 | 6 | 4 | 16 | 12 | 6 | 85 | 39 | 7 |
| chr15:62299507\|62306191 | hsa_circ_0000607 | 63 | 50 | 27 | 17 | 23 | 7 | 22 | 29 | 11 | 436 | 155 | 52 |
| chr15:60734615\|60737990 | hsa_circ_0000606 | 56 | 53 | 16 | 50 | 46 | 23 | 16 | 16 | 16 | 317 | 159 | 33 |
| chr15:59204762\|59209198 | hsa_circ_0000605 | 382 | 301 | 109 | 194 | 247 | 141 | 321 | 396 | 243 | 2705 | 1306 | 401 |
| chr15:59204762\|59205895 | hsa_circ_0003713 | 75 | 67 | 27 | 36 | 41 | 24 | 44 | 53 | 19 | 380 | 189 | 69 |
| chr15:56686363\|56687032 | #N/A | 20 | 26 | 12 | 12 | 11 | 2 | 2 | 4 | 4 | 90 | 36 | 18 |
| chr15:50875286\|50884823 | hsa_circ_0035228 | 20 | 23 | 9 | 8 | 10 | 3 | 4 | 2 | 3 | 65 | 24 | 10 |
| chr15:50741597\|50751359 | hsa_circ_0035214 | 6 | 9 | 3 | 4 | 2 | 3 | 7 | 8 | 6 | 51 | 31 | 6 |
| chr15:50592986\|50593565 | hsa_circ_0002551 | 17 | 25 | 9 | 28 | 25 | 9 | 20 | 22 | 5 | 136 | 68 | 17 |
| chr15:49528048\|49531564 | hsa_circ_0008488 | 8 | 10 | 6 | 15 | 5 | 2 | 2 | 4 | 6 | 75 | 28 | 10 |
| chr15:49429347\|49431850 | #N/A | 10 | 3 | 2 | 10 | 6 | 2 | 10 | 9 | 7 | 79 | 34 | 19 |
| chr15:44843074\|44843720 | #N/A | 28 | 20 | 7 | 16 | 12 | 21 | 6 | 5 | 3 | 97 | 25 | 5 |
| chr15:44624186\|44630515 | hsa_circ_0000596 | 20 | 14 | 12 | 9 | 12 | 6 | 7 | 10 | 11 | 149 | 32 | 19 |
| chr15:43692242\|43694048 | hsa_circ_0034972 | 5 | 7 | 2 | 15 | 9 | 6 | 10 | 10 | 2 | 89 | 44 | 13 |
| chr15:43627143\|43628024 | hsa_circ_0034947 | 22 | 26 | 9 | 15 | 22 | 10 | 10 | 31 | 16 | 212 | 87 | 34 |
| chr15:43120126\|43132631 | hsa_circ_0008319 | 6 | 6 | 6 | 4 | 6 | 3 | 4 | 7 | 4 | 53 | 23 | 22 |
| chr15:42851537\|42856055 | #N/A | 22 | 12 | 6 | 18 | 16 | 6 | 10 | 15 | 13 | 187 | 48 | 25 |
| chr15:42553156\|42560230 | hsa_circ_0034803 | 23 | 15 | 8 | 9 | 16 | 6 | 14 | 26 | 7 | 151 | 41 | 20 |
| chr15:41961026\|41962156 | hsa_circ_0000591 | 59 | 41 | 19 | 7 | 14 | 13 | 17 | 19 | 14 | 201 | 90 | 36 |
| chr15:41667910\|41669502 | hsa_circ_0002124 | 186 | 198 | 91 | 144 | 164 | 108 | 93 | 79 | 64 | 773 | 352 | 160 |
| chr15:41663725\|41669502 | #N/A | 14 | 10 | 5 | 10 | 17 | 3 | 12 | 17 | 14 | 142 | 75 | 36 |
| chr15:41648237\|41669502 | hsa_circ_0008346 | 63 | 60 | 22 | 34 | 25 | 11 | 37 | 44 | 23 | 401 | 194 | 62 |
| chr15:41377567\|41379880 | hsa_circ_0004279 | 8 | 7 | 4 | 8 | 11 | 10 | 7 | 5 | 5 | 71 | 29 | 10 |
| chr15:40920268\|40943017 | #N/A | 10 | 16 | 8 | 9 | 2 | 5 | 12 | 2 | 3 | 54 | 37 | 5 |
| chr15:40920268\|40921569 | #N/A | 6 | 7 | 2 | 7 | 8 | 5 | 7 | 9 | 5 | 74 | 32 | 8 |
| chr15:31266517\|31269158 | #N/A | 12 | 13 | 8 | 8 | 16 | 4 | 18 | 18 | 6 | 191 | 64 | 20 |
| chr15:25650608\|25657118 | hsa_circ_0000586 | 24 | 19 | 7 | 23 | 28 | 21 | 23 | 70 | 31 | 290 | 146 | 86 |
| chr15:101104897\|101105470 | hsa_circ_0003007 | 217 | 205 | 48 | 169 | 190 | 116 | 152 | 109 | 81 | 1644 | 941 | 167 |
| chr14:99924616\|99932150 | hsa_circ_0000567 | 92 | 94 | 37 | 60 | 66 | 41 | 90 | 89 | 74 | 720 | 404 | 166 |
| chr14:99723808\|99724176 | hsa_circ_0033144 | 25 | 21 | 5 | 18 | 21 | 4 | 39 | 60 | 37 | 168 | 115 | 31 |
| chr14:97299804\|97327072 | hsa_circ_0000566 | 142 | 108 | 55 | 26 | 24 | 30 | 49 | 43 | 23 | 468 | 122 | 62 |
| chr14:97026986\|97031551 | #N/A | 19 | 9 | 7 | 14 | 19 | 8 | 17 | 39 | 9 | 174 | 98 | 26 |
| chr14:97026986\|97029230 | hsa_circ_0033126 | 75 | 48 | 12 | 38 | 57 | 14 | 11 | 13 | 5 | 759 | 264 | 19 |
| chr14:97022184\|97022750 | hsa_circ_0006717 | 13 | 7 | 4 | 13 | 12 | 9 | 14 | 14 | 5 | 122 | 42 | 15 |
| chr14:96986392\|96991728 | hsa_circ_0002120 | 18 | 20 | 5 | 15 | 9 | 10 | 16 | 34 | 12 | 201 | 57 | 24 |
| chr14:92537279\|92548810 | #N/A | 8 | 5 | 3 | 3 | 5 | 2 | 6 | 7 | 9 | 41 | 23 | 8 |
| chr14:92473984\|92477416 | #N/A | 16 | 16 | 3 | 17 | 10 | 3 | 15 | 10 | 13 | 128 | 49 | 21 |
| chr14:91444650\|91467503 | hsa_circ_0032943 | 7 | 2 | 6 | 7 | 4 | 2 | 6 | 3 | 7 | 54 | 21 | 7 |
| chr14:81297487\|81307112 | hsa_circ_0032825 | 29 | 29 | 5 | 11 | 12 | 14 | 3 | 11 | 9 | 155 | 50 | 28 |
| chr14:81297487\|81304622 | #N/A | 13 | 8 | 3 | 9 | 9 | 5 | 14 | 16 | 13 | 109 | 60 | 18 |
| chr14:76633006\|76662315 | hsa_circ_0005252 | 11 | 4 | 3 | 4 | 4 | 2 | 9 | 8 | 4 | 55 | 24 | 9 |
| chr14:75136352\|75138186 | hsa_circ_0005030 | 8 | 6 | 6 | 6 | 9 | 3 | 16 | 30 | 19 | 99 | 53 | 13 |
| chr14:74495887\|74512833 | #N/A | 18 | 14 | 9 | 8 | 6 | 5 | 5 | 7 | 3 | 129 | 30 | 12 |
| chr14:73614503\|73614802 | hsa_circ_0008521 | 16 | 23 | 8 | 16 | 8 | 5 | 5 | 4 | 3 | 176 | 113 | 18 |
| chr14:73181131\|73198642 | #N/A | 17 | 11 | 4 | 7 | 18 | 12 | 8 | 16 | 5 | 62 | 49 | 19 |
| chr14:71880665\|71996087 | #N/A | 5 | 4 | 5 | 5 | 5 | 2 | 5 | 6 | 6 | 87 | 24 | 14 |
| chr14:71867511\|71884913 | hsa_circ_0002722 | 4 | 5 | 3 | 6 | 2 | 4 | 3 | 2 | 9 | 69 | 27 | 10 |
| chr14:68151732\|68157138 | hsa_circ_0006278 | 12 | 14 | 4 | 7 | 18 | 6 | 13 | 20 | 15 | 105 | 58 | 30 |
| chr14:59730159\|59758024 | #N/A | 13 | 11 | 5 | 12 | 5 | 2 | 14 | 4 | 2 | 96 | 43 | 16 |
| chr14:58785260\|58796887 | hsa_circ_0000542 | 13 | 14 | 3 | 6 | 6 | 7 | 10 | 11 | 4 | 106 | 42 | 15 |
| chr14:55847291\|55848909 | #N/A | 6 | 9 | 4 | 7 | 11 | 9 | 11 | 16 | 10 | 108 | 50 | 19 |
| chr14:55647931\|55650471 | hsa_circ_0000539 | 29 | 31 | 9 | 13 | 17 | 20 | 21 | 27 | 15 | 341 | 114 | 41 |
| chr14:55423752\|55424353 | hsa_circ_0006214 | 32 | 26 | 9 | 25 | 32 | 12 | 7 | 8 | 12 | 124 | 62 | 23 |
| chr14:54875463\|54882648 | hsa_circ_0031978 | 23 | 35 | 11 | 21 | 26 | 10 | 25 | 30 | 17 | 299 | 117 | 30 |
| chr14:53003437\|53011089 | hsa_circ_0031940 | 84 | 78 | 24 | 25 | 26 | 19 | 27 | 32 | 10 | 560 | 198 | 45 |
| chr14:51107468\|51111732 | hsa_circ_0007101 | 3 | 4 | 3 | 2 | 2 | 2 | 3 | 7 | 6 | 45 | 17 | 14 |
| chr14:50933301\|50943024 | #N/A | 24 | 21 | 8 | 9 | 15 | 5 | 20 | 36 | 22 | 288 | 104 | 48 |
| chr14:50616726\|50619891 | #N/A | 8 | 3 | 3 | 8 | 3 | 5 | 14 | 7 | 5 | 74 | 36 | 22 |
| chr14:50616726\|50616948 | hsa_circ_0007695 | 44 | 58 | 26 | 34 | 42 | 26 | 17 | 17 | 21 | 209 | 128 | 43 |
| chr14:50292585\|50298079 | #N/A | 43 | 25 | 13 | 16 | 11 | 9 | 6 | 3 | 2 | 157 | 61 | 13 |
| chr14:50136242\|50141145 | hsa_circ_0004904 | 105 | 113 | 39 | 82 | 105 | 42 | 71 | 45 | 27 | 830 | 373 | 123 |
| chr14:50131344\|50141145 | hsa_circ_0031787 | 16 | 11 | 7 | 9 | 12 | 4 | 13 | 12 | 6 | 155 | 47 | 19 |
| chr14:50130033\|50141145 | hsa_circ_0008002 | 77 | 52 | 13 | 30 | 24 | 9 | 53 | 75 | 28 | 653 | 279 | 100 |
| chr14:45587231\|45599993 | #N/A | 60 | 48 | 27 | 17 | 17 | 17 | 17 | 16 | 9 | 401 | 103 | 51 |
| chr14:39870380\|39871715 | hsa_circ_0005108 | 14 | 3 | 6 | 4 | 2 | 3 | 13 | 7 | 5 | 62 | 26 | 18 |
| chr14:39746138\|39748741 | hsa_circ_0000530 | 17 | 21 | 3 | 13 | 25 | 23 | 15 | 26 | 17 | 164 | 79 | 31 |
| chr14:39648295\|39648666 | #N/A | 131 | 118 | 60 | 78 | 73 | 50 | 61 | 61 | 34 | 710 | 223 | 83 |
| chr14:39627489\|39628754 | hsa_circ_0002395 | 14 | 10 | 9 | 8 | 9 | 5 | 7 | 4 | 5 | 63 | 35 | 12 |
| chr14:39623415\|39628754 | #N/A | 10 | 11 | 2 | 8 | 5 | 6 | 19 | 12 | 3 | 98 | 28 | 9 |
| chr14:39620950\|39628754 | hsa_circ_0031738 | 15 | 12 | 7 | 10 | 12 | 7 | 24 | 16 | 11 | 157 | 48 | 22 |
| chr14:35438376\|35441270 | hsa_circ_0008568 | 12 | 8 | 3 | 4 | 5 | 2 | 5 | 5 | 5 | 32 | 15 | 7 |
| chr14:35331250\|35331528 | hsa_circ_0006137 | 74 | 82 | 25 | 86 | 74 | 37 | 86 | 90 | 46 | 846 | 372 | 161 |
| chr14:35020920\|35024118 | hsa_circ_0007379 | 34 | 24 | 7 | 30 | 31 | 15 | 35 | 28 | 15 | 272 | 134 | 47 |
| chr14:31602444\|31602881 | hsa_circ_0031485 | 8 | 7 | 4 | 12 | 7 | 6 | 19 | 24 | 11 | 93 | 45 | 26 |
| chr14:31424826\|31425448 | hsa_circ_0007656 | 27 | 30 | 8 | 20 | 23 | 13 | 8 | 7 | 5 | 177 | 58 | 14 |
| chr14:31420069\|31425448 | #N/A | 18 | 14 | 3 | 9 | 5 | 3 | 15 | 11 | 4 | 87 | 34 | 12 |
| chr14:31416296\|31425448 | hsa_circ_0031447 | 116 | 75 | 25 | 28 | 37 | 23 | 53 | 65 | 46 | 623 | 193 | 97 |
| chr14:31416296\|31420150 | #N/A | 14 | 19 | 6 | 5 | 3 | 6 | 10 | 5 | 4 | 44 | 22 | 7 |
| chr14:31404369\|31425448 | hsa_circ_0031446 | 48 | 55 | 23 | 34 | 26 | 11 | 27 | 46 | 20 | 328 | 147 | 69 |
| chr14:31398407\|31425448 | #N/A | 7 | 10 | 4 | 4 | 2 | 7 | 6 | 3 | 5 | 37 | 26 | 6 |
| chr14:31388172\|31398517 | #N/A | 11 | 9 | 3 | 9 | 6 | 4 | 5 | 14 | 8 | 82 | 42 | 7 |
| chr14:31371742\|31380368 | hsa_circ_0031433 | 17 | 12 | 6 | 10 | 15 | 2 | 12 | 12 | 10 | 165 | 75 | 30 |
| chr14:31185130\|31204064 | hsa_circ_0000528 | 72 | 64 | 27 | 43 | 52 | 32 | 43 | 50 | 33 | 610 | 220 | 83 |
| chr14:31139462\|31144271 | hsa_circ_0009140 | 50 | 24 | 15 | 19 | 40 | 14 | 56 | 72 | 31 | 606 | 238 | 90 |
| chr14:31050070\|31050322 | #N/A | 48 | 43 | 12 | 24 | 34 | 16 | 6 | 3 | 3 | 440 | 131 | 21 |
| chr14:24680621\|24681035 | hsa_circ_0031359 | 20 | 8 | 5 | 16 | 8 | 5 | 11 | 14 | 6 | 94 | 51 | 22 |
| chr14:24680617\|24681035 | #N/A | 4 | 8 | 3 | 6 | 7 | 2 | 4 | 5 | 8 | 29 | 11 | 7 |
| chr14:23378692\|23380612 | hsa_circ_0000524 | 245 | 237 | 113 | 179 | 207 | 150 | 185 | 135 | 113 | 1365 | 745 | 270 |
| chr14:23375404\|23380612 | hsa_circ_0031241 | 88 | 81 | 27 | 55 | 46 | 18 | 74 | 119 | 68 | 540 | 356 | 122 |
| chr14:23375404\|23378804 | hsa_circ_0004137 | 9 | 8 | 5 | 9 | 13 | 2 | 7 | 12 | 3 | 73 | 27 | 14 |
| chr14:21930509\|21931926 | #N/A | 17 | 17 | 6 | 10 | 23 | 6 | 9 | 2 | 5 | 105 | 38 | 12 |
| chr14:21698478\|21702388 | hsa_circ_0003643 | 27 | 28 | 22 | 17 | 33 | 11 | 49 | 79 | 39 | 411 | 222 | 74 |
| chr14:104037960\|104053701 | #N/A | 10 | 7 | 4 | 4 | 3 | 6 | 7 | 7 | 3 | 80 | 28 | 12 |
| chr14:103923479\|103928798 | hsa_circ_0033480 | 14 | 4 | 2 | 4 | 5 | 8 | 6 | 3 | 5 | 66 | 24 | 9 |
| chr14:103918255\|103928798 | hsa_circ_0033476 | 24 | 16 | 3 | 23 | 15 | 13 | 3 | 4 | 4 | 108 | 56 | 15 |
| chr14:103918255\|103923549 | hsa_circ_0033475 | 32 | 25 | 7 | 30 | 35 | 21 | 9 | 4 | 5 | 513 | 214 | 11 |
| chr14:103894724\|103928798 | #N/A | 22 | 14 | 9 | 23 | 14 | 5 | 17 | 14 | 6 | 232 | 49 | 39 |
| chr14:103871413\|103894777 | hsa_circ_0033472 | 13 | 11 | 4 | 6 | 7 | 8 | 19 | 13 | 7 | 86 | 42 | 20 |
| chr14:103865288\|103894777 | hsa_circ_0008780 | 10 | 18 | 6 | 9 | 11 | 2 | 13 | 23 | 12 | 146 | 57 | 24 |
| chr14:103865288\|103871604 | hsa_circ_0008282 | 127 | 88 | 24 | 59 | 86 | 36 | 100 | 123 | 78 | 968 | 438 | 152 |
| chr14:102661275\|102664184 | hsa_circ_0003512 | 13 | 13 | 5 | 8 | 14 | 3 | 8 | 14 | 5 | 93 | 45 | 23 |
| chr14:102659800\|102661457 | hsa_circ_0000569 | 33 | 33 | 10 | 19 | 30 | 5 | 25 | 17 | 22 | 155 | 86 | 41 |
| chr14:102368056\|102372866 | hsa_circ_0003831 | 29 | 20 | 5 | 20 | 36 | 7 | 36 | 38 | 18 | 257 | 99 | 27 |
| chr13:99890681\|99896878 | hsa_circ_0030720 | 34 | 30 | 13 | 60 | 53 | 24 | 26 | 39 | 23 | 312 | 147 | 31 |
| chr13:96636058\|96651561 | hsa_circ_0030632 | 21 | 12 | 12 | 8 | 9 | 3 | 9 | 22 | 7 | 164 | 55 | 27 |
| chr13:96375496\|96377506 | hsa_circ_0002473 | 21 | 25 | 2 | 15 | 22 | 5 | 19 | 30 | 15 | 198 | 86 | 20 |
| chr13:95813443\|95822882 | hsa_circ_0006659 | 19 | 9 | 6 | 4 | 12 | 3 | 21 | 23 | 6 | 149 | 61 | 35 |
| chr13:78293667\|78327493 | hsa_circ_0000497 | 13 | 6 | 5 | 8 | 8 | 3 | 10 | 10 | 6 | 96 | 45 | 13 |
| chr13:76134889\|76143643 | hsa_circ_0000494 | 10 | 12 | 2 | 13 | 9 | 8 | 16 | 23 | 14 | 142 | 69 | 26 |
| chr13:61013822\|61041513 | hsa_circ_0003441 | 12 | 8 | 7 | 11 | 13 | 4 | 22 | 26 | 18 | 146 | 77 | 30 |
| chr13:61013822\|61034674 | hsa_circ_0004245 | 16 | 32 | 11 | 26 | 19 | 6 | 20 | 27 | 21 | 203 | 110 | 43 |
| chr13:52992127\|53001354 | hsa_circ_0030342 | 21 | 19 | 10 | 26 | 11 | 6 | 21 | 14 | 15 | 233 | 112 | 39 |
| chr13:51501543\|51523641 | hsa_circ_0000489 | 23 | 21 | 4 | 15 | 14 | 8 | 15 | 19 | 13 | 238 | 80 | 26 |
| chr13:50642233\|50649789 | hsa_circ_0030281 | 20 | 26 | 8 | 27 | 20 | 14 | 22 | 12 | 11 | 183 | 58 | 16 |
| chr13:50601499\|50619368 | #N/A | 33 | 33 | 14 | 17 | 30 | 14 | 38 | 55 | 20 | 426 | 170 | 57 |
| chr13:50025689\|50026045 | hsa_circ_0030253 | 16 | 17 | 6 | 12 | 11 | 10 | 17 | 19 | 13 | 213 | 70 | 32 |
| chr13:48916735\|48923159 | hsa_circ_0002365 | 9 | 7 | 2 | 4 | 6 | 2 | 7 | 10 | 7 | 114 | 28 | 20 |
| chr13:46090278\|46093229 | hsa_circ_0003401 | 23 | 9 | 9 | 16 | 13 | 5 | 17 | 16 | 5 | 144 | 72 | 20 |
| chr13:43528084\|43544806 | hsa_circ_0000479 | 35 | 30 | 15 | 20 | 27 | 19 | 27 | 61 | 22 | 422 | 198 | 79 |
| chr13:42040959\|42042974 | #N/A | 29 | 32 | 9 | 39 | 41 | 32 | 14 | 9 | 9 | 361 | 188 | 31 |
| chr13:41943226\|41946966 | #N/A | 12 | 17 | 4 | 9 | 6 | 5 | 9 | 25 | 5 | 128 | 33 | 27 |
| chr13:41826781\|41835051 | hsa_circ_0008230 | 19 | 14 | 6 | 11 | 12 | 3 | 20 | 7 | 7 | 153 | 66 | 42 |
| chr13:41826781\|41828756 | #N/A | 8 | 6 | 5 | 6 | 5 | 4 | 2 | 11 | 7 | 105 | 36 | 13 |
| chr13:41587214\|41593568 | #N/A | 8 | 11 | 3 | 3 | 11 | 5 | 7 | 14 | 5 | 94 | 41 | 9 |
| chr13:41517088\|41518061 | #N/A | 20 | 16 | 2 | 8 | 18 | 6 | 10 | 10 | 3 | 90 | 39 | 17 |
| chr13:41507606\|41508164 | #N/A | 10 | 19 | 3 | 4 | 7 | 5 | 10 | 12 | 6 | 62 | 38 | 13 |
| chr13:37614542\|37625720 | hsa_circ_0000475 | 16 | 22 | 5 | 4 | 8 | 2 | 13 | 11 | 6 | 161 | 80 | 28 |
| chr13:33306238\|33309467 | hsa_circ_0004494 | 9 | 6 | 2 | 6 | 3 | 5 | 12 | 12 | 4 | 61 | 30 | 6 |
| chr13:33091994\|33101669 | hsa_circ_0000471 | 497 | 419 | 167 | 355 | 394 | 172 | 591 | 809 | 449 | 4885 | 2448 | 957 |
| chr13:33054727\|33101669 | hsa_circ_0005723 | 5 | 5 | 3 | 4 | 2 | 2 | 13 | 14 | 7 | 95 | 36 | 13 |
| chr13:32893214\|32900750 | hsa_circ_0029929 | 9 | 10 | 8 | 18 | 11 | 5 | 8 | 12 | 3 | 116 | 44 | 10 |
| chr13:30826068\|30829752 | #N/A | 46 | 34 | 19 | 17 | 42 | 16 | 46 | 47 | 17 | 304 | 149 | 46 |
| chr13:28830429\|28835595 | hsa_circ_0008902 | 16 | 17 | 6 | 17 | 20 | 17 | 24 | 24 | 12 | 127 | 83 | 33 |
| chr13:28748409\|28752072 | hsa_circ_0004372 | 71 | 59 | 23 | 77 | 65 | 38 | 64 | 50 | 26 | 473 | 236 | 68 |
| chr13:28155434\|28155940 | hsa_circ_0029827 | 50 | 29 | 16 | 18 | 25 | 12 | 51 | 33 | 20 | 352 | 171 | 59 |
| chr13:26974590\|26975761 | hsa_circ_0003489 | 114 | 108 | 31 | 100 | 122 | 41 | 131 | 131 | 84 | 1004 | 503 | 139 |
| chr13:25887021\|25889605 | hsa_circ_0029787 | 5 | 5 | 3 | 5 | 2 | 5 | 5 | 5 | 4 | 46 | 24 | 10 |
| chr13:21987791\|21999817 | hsa_circ_0006732 | 56 | 66 | 14 | 66 | 57 | 29 | 56 | 39 | 20 | 561 | 198 | 52 |
| chr13:21974512\|21999817 | hsa_circ_0029708 | 25 | 28 | 8 | 27 | 32 | 8 | 33 | 22 | 10 | 356 | 113 | 45 |
| chr13:21974512\|21995303 | #N/A | 23 | 6 | 4 | 6 | 17 | 9 | 14 | 9 | 3 | 151 | 39 | 12 |
| chr13:21742127\|21742538 | hsa_circ_0000467 | 137 | 102 | 38 | 112 | 124 | 54 | 118 | 120 | 77 | 1518 | 474 | 181 |
| chr13:21735929\|21746820 | hsa_circ_0029696 | 20 | 16 | 10 | 2 | 6 | 2 | 6 | 3 | 4 | 90 | 17 | 10 |
| chr13:21729832\|21732264 | #N/A | 24 | 19 | 8 | 12 | 28 | 10 | 23 | 5 | 6 | 230 | 68 | 23 |
| chr13:20304379\|20356931 | hsa_circ_0029614 | 17 | 11 | 7 | 4 | 12 | 13 | 12 | 11 | 9 | 83 | 44 | 24 |
| chr13:114806476\|114839312 | hsa_circ_0004096 | 5 | 6 | 2 | 5 | 7 | 3 | 10 | 7 | 9 | 50 | 44 | 15 |
| chr13:114806476\|114822949 | hsa_circ_0004790 | 25 | 17 | 8 | 12 | 12 | 11 | 30 | 27 | 18 | 130 | 113 | 42 |
| chr13:114265311\|114277601 | hsa_circ_0031038 | 34 | 29 | 17 | 37 | 43 | 34 | 27 | 11 | 13 | 209 | 154 | 35 |
| chr12:97303530\|97313903 | #N/A | 13 | 5 | 7 | 7 | 10 | 7 | 10 | 5 | 4 | 83 | 30 | 10 |
| chr12:96717726\|96728643 | hsa_circ_0002762 | 71 | 41 | 10 | 47 | 46 | 14 | 65 | 70 | 29 | 475 | 229 | 83 |
| chr12:96692647\|96694138 | hsa_circ_0007881 | 43 | 33 | 11 | 15 | 21 | 14 | 12 | 4 | 3 | 360 | 133 | 22 |
| chr12:94562929\|94580249 | #N/A | 28 | 23 | 7 | 21 | 21 | 7 | 27 | 36 | 22 | 283 | 103 | 55 |
| chr12:938228\|939110 | hsa_circ_0005616 | 228 | 165 | 64 | 150 | 172 | 85 | 47 | 31 | 25 | 1465 | 581 | 69 |
| chr12:922808\|939326 | hsa_circ_0007892 | 8 | 8 | 5 | 11 | 4 | 2 | 22 | 16 | 9 | 90 | 68 | 30 |
| chr12:89860547\|89866052 | hsa_circ_0027702 | 39 | 30 | 17 | 14 | 27 | 11 | 31 | 41 | 12 | 399 | 161 | 68 |
| chr12:89853415\|89866052 | #N/A | 27 | 12 | 4 | 12 | 17 | 4 | 18 | 26 | 11 | 239 | 58 | 33 |
| chr12:72163580\|72167802 | #N/A | 11 | 2 | 3 | 6 | 6 | 2 | 4 | 13 | 6 | 70 | 15 | 8 |
| chr12:72051306\|72054207 | hsa_circ_0006910 | 10 | 13 | 9 | 12 | 13 | 9 | 4 | 5 | 9 | 114 | 36 | 20 |
| chr12:70193989\|70195501 | hsa_circ_0000419 | 44 | 56 | 18 | 42 | 48 | 31 | 26 | 20 | 13 | 323 | 154 | 56 |
| chr12:70149164\|70150443 | #N/A | 30 | 23 | 15 | 22 | 10 | 3 | 30 | 36 | 19 | 242 | 100 | 35 |
| chr12:69983265\|69985939 | hsa_circ_0000418 | 187 | 194 | 59 | 88 | 112 | 61 | 77 | 62 | 46 | 1556 | 419 | 141 |
| chr12:69644909\|69656342 | hsa_circ_0000417 | 26 | 34 | 14 | 12 | 18 | 17 | 4 | 14 | 6 | 55 | 47 | 14 |
| chr12:69222551\|69230529 | hsa_circ_0002770 | 14 | 10 | 6 | 4 | 7 | 4 | 16 | 12 | 17 | 89 | 29 | 14 |
| chr12:69107645\|69108533 | #N/A | 29 | 22 | 15 | 16 | 25 | 11 | 3 | 3 | 4 | 174 | 84 | 29 |
| chr12:68696400\|68696652 | hsa_circ_0027459 | 8 | 10 | 3 | 12 | 9 | 3 | 8 | 6 | 3 | 50 | 24 | 5 |
| chr12:62743002\|62749256 | hsa_circ_0027355 | 40 | 38 | 12 | 35 | 36 | 27 | 24 | 21 | 9 | 149 | 97 | 30 |
| chr12:62719653\|62749256 | hsa_circ_0027354 | 10 | 6 | 9 | 14 | 16 | 4 | 9 | 5 | 7 | 139 | 68 | 21 |
| chr12:62715245\|62749256 | hsa_circ_0027353 | 25 | 13 | 13 | 11 | 20 | 10 | 11 | 15 | 11 | 180 | 66 | 25 |
| chr12:58340778\|58347472 | hsa_circ_0000412 | 36 | 36 | 20 | 22 | 36 | 14 | 64 | 43 | 27 | 366 | 140 | 58 |
| chr12:58339411\|58347472 | #N/A | 20 | 21 | 10 | 12 | 19 | 3 | 21 | 35 | 14 | 233 | 99 | 48 |
| chr12:57463017\|57466695 | hsa_circ_0000411 | 19 | 5 | 11 | 15 | 7 | 4 | 7 | 13 | 6 | 180 | 73 | 11 |
| chr12:57059988\|57064148 | hsa_circ_0027089 | 114 | 93 | 38 | 119 | 143 | 72 | 150 | 65 | 59 | 1103 | 576 | 120 |
| chr12:51449618\|51450285 | hsa_circ_0004313 | 3 | 6 | 3 | 6 | 8 | 3 | 6 | 10 | 4 | 30 | 24 | 5 |
| chr12:50488220\|50490755 | hsa_circ_0006535 | 8 | 9 | 4 | 13 | 12 | 2 | 3 | 4 | 4 | 30 | 29 | 10 |
| chr12:49722710\|49723237 | hsa_circ_0026143 | 3 | 4 | 5 | 3 | 5 | 2 | 4 | 4 | 7 | 21 | 12 | 4 |
| chr12:48465450\|48468576 | hsa_circ_0007838 | 8 | 14 | 6 | 6 | 2 | 3 | 7 | 13 | 7 | 89 | 39 | 13 |
| chr12:47497896\|47501947 | hsa_circ_0004650 | 14 | 9 | 4 | 6 | 6 | 4 | 12 | 18 | 4 | 55 | 33 | 17 |
| chr12:46633462\|46637097 | hsa_circ_0000398 | 11 | 7 | 5 | 6 | 7 | 7 | 18 | 18 | 18 | 135 | 57 | 12 |
| chr12:46622936\|46637097 | hsa_circ_0000396 | 41 | 17 | 7 | 16 | 18 | 5 | 27 | 39 | 16 | 320 | 110 | 44 |
| chr12:42778742\|42792796 | hsa_circ_0025908 | 8 | 19 | 7 | 3 | 9 | 6 | 16 | 22 | 5 | 132 | 56 | 27 |
| chr12:32751431\|32764217 | hsa_circ_0025843 | 21 | 15 | 5 | 9 | 7 | 2 | 7 | 17 | 8 | 134 | 60 | 19 |
| chr12:2929252\|2930958 | hsa_circ_0000374 | 8 | 8 | 2 | 4 | 10 | 6 | 6 | 14 | 4 | 48 | 41 | 16 |
| chr12:28458582\|28544344 | hsa_circ_0008250 | 7 | 8 | 5 | 6 | 6 | 3 | 7 | 15 | 7 | 78 | 37 | 16 |
| chr12:28458582\|28460682 | hsa_circ_0007723 | 25 | 19 | 5 | 17 | 11 | 8 | 26 | 26 | 16 | 249 | 93 | 40 |
| chr12:28408514\|28412375 | hsa_circ_0000387 | 23 | 21 | 6 | 22 | 31 | 17 | 23 | 19 | 20 | 254 | 95 | 28 |
| chr12:28378728\|28412375 | hsa_circ_0000386 | 71 | 64 | 22 | 64 | 74 | 29 | 61 | 106 | 68 | 974 | 324 | 143 |
| chr12:27867713\|27877119 | hsa_circ_0000384 | 6 | 13 | 5 | 6 | 12 | 13 | 12 | 16 | 12 | 125 | 44 | 17 |
| chr12:27183667\|27185614 | #N/A | 24 | 16 | 14 | 18 | 25 | 13 | 20 | 16 | 9 | 183 | 94 | 20 |
| chr12:27107078\|27110676 | #N/A | 52 | 34 | 17 | 26 | 31 | 11 | 40 | 38 | 23 | 441 | 168 | 64 |
| chr12:19615444\|19626289 | hsa_circ_0006420 | 8 | 14 | 5 | 5 | 8 | 2 | 9 | 6 | 6 | 77 | 42 | 17 |
| chr12:1863424\|1863680 | hsa_circ_0025002 | 13 | 11 | 6 | 15 | 12 | 6 | 10 | 4 | 11 | 97 | 51 | 16 |
| chr12:1839139\|1863680 | #N/A | 13 | 16 | 4 | 2 | 10 | 7 | 12 | 9 | 6 | 66 | 34 | 8 |
| chr12:1812052\|1863680 | #N/A | 63 | 35 | 10 | 30 | 47 | 16 | 37 | 71 | 31 | 470 | 221 | 72 |
| chr12:14599922\|14610229 | hsa_circ_0025511 | 10 | 33 | 4 | 21 | 25 | 7 | 11 | 8 | 7 | 79 | 40 | 10 |
| chr12:129299320\|129299615 | hsa_circ_0000462 | 13 | 20 | 3 | 13 | 15 | 4 | 18 | 9 | 11 | 99 | 53 | 21 |
| chr12:124904503\|124915333 | hsa_circ_0029308 | 11 | 9 | 2 | 7 | 6 | 5 | 11 | 4 | 11 | 48 | 35 | 16 |
| chr12:124071294\|124074996 | hsa_circ_0000458 | 95 | 93 | 46 | 73 | 77 | 38 | 97 | 88 | 73 | 783 | 346 | 106 |
| chr12:123983091\|123984083 | hsa_circ_0007552 | 4 | 4 | 3 | 8 | 3 | 2 | 6 | 8 | 3 | 23 | 13 | 5 |
| chr12:123879594\|123880979 | #N/A | 11 | 8 | 3 | 9 | 19 | 8 | 8 | 11 | 6 | 62 | 33 | 12 |
| chr12:123829805\|123834988 | hsa_circ_0002510 | 10 | 7 | 2 | 2 | 7 | 5 | 4 | 6 | 6 | 61 | 32 | 10 |
| chr12:123678933\|123682881 | hsa_circ_0006615 | 15 | 12 | 7 | 17 | 15 | 13 | 15 | 16 | 8 | 173 | 61 | 19 |
| chr12:123030723\|123032516 | hsa_circ_0029098 | 15 | 13 | 5 | 7 | 13 | 3 | 16 | 17 | 8 | 116 | 65 | 19 |
| chr12:122995656\|122999774 | #N/A | 32 | 33 | 14 | 23 | 24 | 14 | 15 | 18 | 14 | 174 | 100 | 18 |
| chr12:122973933\|122977336 | #N/A | 7 | 3 | 5 | 5 | 8 | 5 | 10 | 8 | 10 | 59 | 21 | 5 |
| chr12:121220458\|121222396 | hsa_circ_0003472 | 188 | 198 | 53 | 120 | 147 | 91 | 123 | 107 | 59 | 1276 | 570 | 166 |
| chr12:120995085\|120995485 | hsa_circ_0028899 | 31 | 31 | 13 | 28 | 32 | 17 | 28 | 43 | 25 | 385 | 172 | 76 |
| chr12:120592774\|120593523 | hsa_circ_0000448 | 16 | 8 | 3 | 6 | 11 | 6 | 20 | 22 | 12 | 103 | 73 | 37 |
| chr12:120198743\|120204986 | #N/A | 4 | 3 | 2 | 5 | 7 | 4 | 3 | 4 | 3 | 21 | 14 | 8 |
| chr12:116668338\|116675510 | hsa_circ_0000443 | 63 | 47 | 25 | 54 | 45 | 29 | 94 | 160 | 76 | 885 | 390 | 147 |
| chr12:116668238\|116675510 | hsa_circ_0000442 | 58 | 42 | 18 | 40 | 41 | 14 | 61 | 99 | 55 | 575 | 260 | 87 |
| chr12:116534474\|116549317 | hsa_circ_0000441 | 14 | 7 | 3 | 12 | 5 | 3 | 14 | 10 | 5 | 114 | 54 | 16 |
| chr12:112915455\|112915819 | hsa_circ_0008666 | 13 | 12 | 2 | 9 | 6 | 6 | 4 | 4 | 3 | 35 | 39 | 15 |
| chr12:11273609\|11281962 | #N/A | 3 | 4 | 5 | 7 | 8 | 3 | 9 | 10 | 9 | 88 | 51 | 16 |
| chr12:11273609\|11276786 | hsa_circ_0000377 | 11 | 8 | 3 | 14 | 7 | 3 | 7 | 14 | 7 | 88 | 40 | 18 |
| chr12:112667502\|112670889 | #N/A | 33 | 20 | 14 | 11 | 15 | 13 | 26 | 38 | 25 | 184 | 137 | 43 |
| chr12:112641440\|112642427 | #N/A | 7 | 6 | 2 | 12 | 7 | 8 | 9 | 13 | 10 | 95 | 44 | 14 |
| chr12:112616735\|112617201 | #N/A | 15 | 8 | 2 | 8 | 9 | 8 | 16 | 18 | 14 | 88 | 45 | 21 |
| chr12:112116955\|112121111 | hsa_circ_0006396 | 21 | 13 | 8 | 12 | 13 | 5 | 21 | 12 | 14 | 186 | 76 | 21 |
| chr12:112096540\|112097149 | hsa_circ_0007885 | 20 | 15 | 9 | 17 | 18 | 6 | 15 | 20 | 11 | 145 | 71 | 18 |
| chr12:111990084\|111993723 | hsa_circ_0002457 | 14 | 15 | 3 | 12 | 12 | 6 | 11 | 24 | 9 | 153 | 46 | 17 |
| chr12:111947353\|111948386 | hsa_circ_0002601 | 28 | 25 | 8 | 22 | 22 | 12 | 12 | 20 | 6 | 306 | 99 | 44 |
| chr12:111923075\|111924628 | hsa_circ_0002273 | 17 | 16 | 2 | 30 | 33 | 14 | 18 | 13 | 15 | 121 | 66 | 25 |
| chr12:111923069\|111924628 | hsa_circ_0005756 | 12 | 15 | 6 | 17 | 20 | 12 | 21 | 13 | 10 | 158 | 82 | 27 |
| chr12:111920591\|111924628 | hsa_circ_0005981 | 9 | 13 | 5 | 16 | 31 | 6 | 17 | 18 | 13 | 139 | 67 | 14 |
| chr12:111916874\|111924628 | #N/A | 2 | 9 | 5 | 5 | 13 | 8 | 12 | 17 | 11 | 46 | 28 | 12 |
| chr12:109046048\|109048186 | hsa_circ_0000437 | 207 | 197 | 68 | 169 | 227 | 99 | 183 | 243 | 183 | 1562 | 955 | 248 |
| chr12:102107868\|102110590 | hsa_circ_0006660 | 25 | 30 | 4 | 22 | 23 | 12 | 32 | 49 | 24 | 301 | 133 | 48 |
| chr12:100676721\|100691953 | hsa_circ_0006258 | 22 | 22 | 14 | 11 | 20 | 9 | 12 | 25 | 15 | 283 | 99 | 40 |
| chr12:1003728\|1006847 | hsa_circ_0003251 | 19 | 16 | 2 | 9 | 12 | 8 | 8 | 4 | 5 | 93 | 42 | 17 |
| chr11:95546096\|95546753 | hsa_circ_0024067 | 10 | 21 | 7 | 16 | 13 | 6 | 33 | 45 | 13 | 194 | 96 | 30 |
| chr11:9462002\|9463744 | hsa_circ_0004007 | 13 | 17 | 6 | 6 | 11 | 3 | 15 | 17 | 15 | 112 | 60 | 17 |
| chr11:85961338\|85963282 | hsa_circ_0008999 | 23 | 11 | 5 | 7 | 13 | 5 | 20 | 7 | 4 | 62 | 43 | 19 |
| chr11:85960513\|85963282 | #N/A | 12 | 7 | 3 | 3 | 5 | 2 | 9 | 9 | 5 | 70 | 32 | 8 |
| chr11:85733410\|85742653 | hsa_circ_0023942 | 57 | 51 | 15 | 47 | 59 | 31 | 37 | 36 | 37 | 496 | 144 | 72 |
| chr11:85723324\|85742653 | hsa_circ_0023940 | 24 | 17 | 9 | 10 | 11 | 5 | 10 | 9 | 3 | 166 | 44 | 25 |
| chr11:85722073\|85742653 | hsa_circ_0000347 | 95 | 84 | 29 | 37 | 55 | 17 | 28 | 31 | 15 | 609 | 158 | 68 |
| chr11:85718585\|85742653 | hsa_circ_0023936 | 160 | 102 | 52 | 67 | 56 | 38 | 36 | 26 | 16 | 857 | 218 | 110 |
| chr11:85707869\|85742653 | hsa_circ_0023923 | 111 | 83 | 41 | 23 | 38 | 28 | 26 | 19 | 11 | 503 | 139 | 43 |
| chr11:85707869\|85714494 | hsa_circ_0023919 | 189 | 177 | 53 | 132 | 193 | 75 | 246 | 313 | 182 | 1638 | 986 | 302 |
| chr11:85707869\|85712201 | hsa_circ_0002513 | 8 | 7 | 4 | 10 | 10 | 8 | 13 | 17 | 6 | 94 | 42 | 16 |
| chr11:85692788\|85701442 | #N/A | 15 | 10 | 9 | 8 | 18 | 5 | 16 | 14 | 5 | 153 | 94 | 25 |
| chr11:85685751\|85695016 | hsa_circ_0006629 | 37 | 28 | 10 | 21 | 15 | 9 | 40 | 58 | 29 | 292 | 168 | 58 |
| chr11:85685751\|85692271 | hsa_circ_0023883 | 24 | 14 | 2 | 18 | 16 | 7 | 14 | 10 | 4 | 123 | 57 | 13 |
| chr11:82984690\|82989872 | #N/A | 22 | 17 | 3 | 6 | 13 | 7 | 14 | 12 | 7 | 192 | 59 | 23 |
| chr11:77433733\|77436744 | #N/A | 8 | 6 | 4 | 5 | 5 | 4 | 5 | 2 | 3 | 41 | 27 | 5 |
| chr11:77394755\|77404656 | hsa_circ_0000344 | 101 | 88 | 21 | 60 | 79 | 43 | 96 | 139 | 78 | 892 | 431 | 162 |
| chr11:77336008\|77336863 | hsa_circ_0000343 | 38 | 30 | 15 | 20 | 27 | 22 | 28 | 25 | 28 | 153 | 112 | 35 |
| chr11:77330651\|77336863 | hsa_circ_0023694 | 41 | 41 | 17 | 19 | 33 | 12 | 27 | 15 | 5 | 252 | 121 | 30 |
| chr11:76237506\|76239510 | hsa_circ_0023659 | 24 | 24 | 2 | 5 | 5 | 6 | 6 | 22 | 6 | 139 | 66 | 23 |
| chr11:76174865\|76183884 | hsa_circ_0007458 | 20 | 15 | 6 | 26 | 18 | 15 | 18 | 23 | 14 | 100 | 66 | 13 |
| chr11:755879\|760253 | hsa_circ_0003307 | 19 | 12 | 5 | 8 | 8 | 2 | 13 | 18 | 9 | 121 | 76 | 21 |
| chr11:74500671\|74528759 | hsa_circ_0006705 | 20 | 15 | 6 | 17 | 27 | 5 | 16 | 21 | 3 | 213 | 98 | 27 |
| chr11:73843889\|73844602 | hsa_circ_0023558 | 13 | 15 | 8 | 36 | 37 | 17 | 13 | 13 | 5 | 156 | 91 | 28 |
| chr11:73418465\|73431944 | hsa_circ_0003908 | 28 | 14 | 5 | 17 | 12 | 8 | 21 | 21 | 8 | 165 | 95 | 37 |
| chr11:73418465\|73429763 | hsa_circ_0000339 | 61 | 48 | 22 | 41 | 33 | 26 | 27 | 60 | 26 | 434 | 194 | 66 |
| chr11:72695133\|72700142 | hsa_circ_0005918 | 18 | 17 | 9 | 9 | 11 | 3 | 11 | 9 | 9 | 121 | 54 | 6 |
| chr11:68359044\|68367962 | hsa_circ_0001968 | 10 | 7 | 3 | 13 | 8 | 3 | 20 | 24 | 8 | 149 | 53 | 17 |
| chr11:68350511\|68363686 | #N/A | 7 | 10 | 4 | 4 | 3 | 3 | 6 | 11 | 7 | 103 | 54 | 20 |
| chr11:68334482\|68343511 | hsa_circ_0002038 | 25 | 25 | 8 | 13 | 15 | 7 | 15 | 7 | 16 | 170 | 101 | 32 |
| chr11:68318589\|68331900 | hsa_circ_0007660 | 76 | 85 | 29 | 38 | 70 | 22 | 69 | 63 | 47 | 749 | 321 | 116 |
| chr11:67953248\|67957619 | #N/A | 10 | 21 | 3 | 12 | 7 | 2 | 15 | 31 | 18 | 127 | 79 | 16 |
| chr11:65198240\|65211534 | #N/A | 7 | 13 | 4 | 7 | 14 | 11 | 12 | 13 | 8 | 100 | 67 | 18 |
| chr11:65198240\|65205128 | hsa_circ_0003812 | 27 | 15 | 4 | 7 | 8 | 3 | 7 | 21 | 11 | 73 | 70 | 18 |
| chr11:61133517\|61135470 | hsa_circ_0002058 | 82 | 68 | 27 | 75 | 84 | 30 | 80 | 64 | 35 | 545 | 298 | 96 |
| chr11:61094251\|61097546 | hsa_circ_0003900 | 6 | 4 | 2 | 7 | 5 | 4 | 11 | 16 | 7 | 72 | 52 | 19 |
| chr11:59426339\|59426942 | hsa_circ_0004162 | 19 | 14 | 9 | 7 | 12 | 7 | 17 | 16 | 10 | 148 | 72 | 25 |
| chr11:57258697\|57259335 | hsa_circ_0005204 | 23 | 24 | 3 | 26 | 26 | 9 | 31 | 32 | 20 | 140 | 101 | 38 |
| chr11:47774468\|47776216 | hsa_circ_0004075 | 10 | 14 | 5 | 15 | 17 | 6 | 15 | 25 | 8 | 116 | 42 | 10 |
| chr11:47380395\|47397280 | hsa_circ_0000303 | 8 | 3 | 2 | 8 | 10 | 8 | 11 | 22 | 17 | 49 | 38 | 20 |
| chr11:46829581\|46831424 | #N/A | 17 | 10 | 4 | 6 | 6 | 3 | 6 | 14 | 11 | 116 | 31 | 18 |
| chr11:46098305\|46113774 | hsa_circ_0000296 | 10 | 12 | 6 | 9 | 14 | 13 | 22 | 21 | 18 | 164 | 80 | 27 |
| chr11:3789811\|3797251 | hsa_circ_0007401 | 22 | 7 | 8 | 8 | 24 | 6 | 16 | 14 | 11 | 84 | 41 | 16 |
| chr11:3752621\|3774638 | hsa_circ_0000274 | 7 | 13 | 5 | 12 | 17 | 3 | 18 | 26 | 9 | 140 | 75 | 31 |
| chr11:3752621\|3752808 | hsa_circ_0002350 | 16 | 20 | 6 | 19 | 25 | 11 | 10 | 8 | 10 | 69 | 64 | 10 |
| chr11:36415396\|36424928 | hsa_circ_0005044 | 8 | 12 | 2 | 8 | 10 | 4 | 6 | 11 | 7 | 78 | 51 | 27 |
| chr11:34991686\|35006275 | hsa_circ_0000290 | 9 | 4 | 4 | 7 | 5 | 3 | 6 | 10 | 5 | 44 | 35 | 7 |
| chr11:34978931\|34999729 | hsa_circ_0021712 | 27 | 18 | 5 | 15 | 14 | 4 | 7 | 10 | 7 | 90 | 31 | 13 |
| chr11:34111726\|34113603 | hsa_circ_0004851 | 24 | 37 | 5 | 16 | 10 | 12 | 16 | 21 | 18 | 195 | 83 | 35 |
| chr11:34111726\|34112225 | hsa_circ_0000288 | 10 | 7 | 3 | 3 | 3 | 2 | 2 | 3 | 3 | 33 | 14 | 12 |
| chr11:34107611\|34107960 | hsa_circ_0021644 | 7 | 4 | 3 | 3 | 3 | 7 | 3 | 12 | 7 | 47 | 22 | 6 |
| chr11:33307959\|33309057 | hsa_circ_0000284 | 85 | 75 | 22 | 32 | 32 | 16 | 45 | 47 | 30 | 467 | 191 | 89 |
| chr11:33163213\|33182915 | #N/A | 9 | 13 | 2 | 12 | 18 | 2 | 10 | 12 | 6 | 43 | 26 | 12 |
| chr11:2991033\|2993473 | hsa_circ_0020822 | 39 | 24 | 13 | 16 | 24 | 11 | 40 | 59 | 23 | 309 | 186 | 66 |
| chr11:28056934\|28058211 | #N/A | 15 | 12 | 6 | 14 | 14 | 4 | 3 | 9 | 7 | 97 | 43 | 14 |
| chr11:16339992\|16362798 | #N/A | 6 | 8 | 2 | 3 | 2 | 2 | 6 | 5 | 6 | 37 | 22 | 9 |
| chr11:16286244\|16291614 | #N/A | 12 | 11 | 6 | 2 | 6 | 4 | 5 | 9 | 7 | 84 | 51 | 12 |
| chr11:16205432\|16256217 | #N/A | 37 | 28 | 13 | 31 | 32 | 14 | 35 | 53 | 24 | 390 | 172 | 58 |
| chr11:16205432\|16208501 | hsa_circ_0021350 | 197 | 207 | 62 | 191 | 173 | 88 | 140 | 164 | 84 | 1066 | 748 | 293 |
| chr11:16203703\|16208501 | #N/A | 9 | 11 | 2 | 11 | 8 | 6 | 8 | 23 | 12 | 107 | 72 | 18 |
| chr11:16199592\|16208501 | #N/A | 10 | 2 | 2 | 6 | 3 | 2 | 11 | 6 | 10 | 73 | 46 | 16 |
| chr11:16133349\|16208501 | #N/A | 73 | 63 | 13 | 50 | 81 | 33 | 100 | 103 | 61 | 582 | 369 | 135 |
| chr11:16119155\|16208501 | hsa_circ_0021347 | 26 | 36 | 9 | 21 | 20 | 10 | 22 | 21 | 16 | 196 | 130 | 44 |
| chr11:16117542\|16208501 | #N/A | 35 | 43 | 19 | 24 | 44 | 13 | 40 | 50 | 20 | 299 | 159 | 78 |
| chr11:16117542\|16133472 | #N/A | 23 | 17 | 6 | 29 | 27 | 18 | 17 | 25 | 19 | 224 | 125 | 41 |
| chr11:16117542\|16133469 | #N/A | 32 | 29 | 4 | 21 | 26 | 8 | 30 | 41 | 18 | 222 | 132 | 36 |
| chr11:16117542\|16119234 | hsa_circ_0021346 | 106 | 98 | 47 | 129 | 145 | 78 | 59 | 50 | 35 | 740 | 459 | 154 |
| chr11:16068060\|16119234 | #N/A | 24 | 18 | 5 | 14 | 24 | 6 | 21 | 35 | 20 | 166 | 142 | 29 |
| chr11:16036488\|16119234 | #N/A | 23 | 26 | 7 | 20 | 24 | 8 | 26 | 43 | 20 | 251 | 119 | 49 |
| chr11:14793483\|14810788 | hsa_circ_0000277 | 30 | 30 | 12 | 20 | 26 | 10 | 20 | 47 | 29 | 299 | 134 | 36 |
| chr11:130130751\|130131824 | hsa_circ_0002484 | 26 | 37 | 19 | 19 | 10 | 18 | 16 | 20 | 14 | 184 | 116 | 30 |
| chr11:128638013\|128651918 | hsa_circ_0024834 | 18 | 5 | 7 | 6 | 15 | 7 | 12 | 16 | 7 | 91 | 65 | 27 |
| chr11:124517261\|124518071 | hsa_circ_0000367 | 6 | 5 | 4 | 7 | 2 | 4 | 6 | 6 | 2 | 45 | 23 | 14 |
| chr11:120916383\|120930794 | hsa_circ_0003302 | 32 | 25 | 9 | 20 | 20 | 21 | 31 | 25 | 24 | 251 | 113 | 48 |
| chr11:120346049\|120348235 | hsa_circ_0002955 | 25 | 19 | 2 | 23 | 11 | 5 | 23 | 54 | 32 | 178 | 152 | 50 |
| chr11:120345269\|120348235 | hsa_circ_0002100 | 18 | 28 | 9 | 19 | 18 | 9 | 24 | 54 | 28 | 322 | 179 | 58 |
| chr11:120343759\|120348235 | hsa_circ_0009021 | 41 | 48 | 17 | 32 | 46 | 18 | 61 | 95 | 54 | 536 | 312 | 116 |
| chr11:120335946\|120338017 | #N/A | 12 | 9 | 4 | 9 | 13 | 4 | 6 | 3 | 9 | 90 | 49 | 14 |
| chr11:120310838\|120317794 | hsa_circ_0024627 | 7 | 5 | 2 | 3 | 5 | 2 | 11 | 6 | 4 | 79 | 35 | 5 |
| chr11:120310838\|120316173 | hsa_circ_0024626 | 14 | 18 | 4 | 12 | 29 | 10 | 23 | 25 | 15 | 171 | 113 | 35 |
| chr11:120276827\|120280159 | hsa_circ_0024605 | 45 | 36 | 9 | 44 | 43 | 20 | 11 | 14 | 8 | 369 | 140 | 36 |
| chr11:120276827\|120278532 | hsa_circ_0024604 | 972 | 729 | 327 | 894 | 900 | 476 | 133 | 105 | 79 | 9576 | 3723 | 242 |
| chr11:120259183\|120280159 | #N/A | 10 | 8 | 2 | 8 | 6 | 6 | 7 | 5 | 5 | 29 | 24 | 10 |
| chr11:119144578\|119145663 | hsa_circ_0000362 | 8 | 9 | 3 | 2 | 3 | 4 | 9 | 3 | 2 | 34 | 20 | 9 |
| chr11:118656761\|118657227 | hsa_circ_0004293 | 7 | 9 | 9 | 6 | 13 | 4 | 13 | 12 | 13 | 153 | 85 | 28 |
| chr11:118454524\|118455359 | hsa_circ_0005589 | 14 | 8 | 2 | 4 | 6 | 9 | 16 | 29 | 19 | 116 | 70 | 22 |
| chr11:118451961\|118455359 | hsa_circ_0006374 | 11 | 17 | 2 | 3 | 5 | 2 | 5 | 5 | 5 | 37 | 31 | 10 |
| chr11:117150624\|117150975 | hsa_circ_0002743 | 7 | 6 | 7 | 10 | 11 | 7 | 3 | 7 | 6 | 82 | 49 | 18 |
| chr11:117023157\|117034608 | hsa_circ_0007827 | 9 | 3 | 3 | 9 | 6 | 3 | 16 | 15 | 11 | 102 | 33 | 16 |
| chr11:111622883\|111624301 | hsa_circ_0024271 | 6 | 5 | 2 | 3 | 12 | 2 | 2 | 6 | 2 | 53 | 22 | 8 |
| chr11:108172375\|108175579 | hsa_circ_0005583 | 3 | 9 | 2 | 3 | 4 | 3 | 2 | 7 | 5 | 69 | 26 | 15 |
| chr11:108137898\|108138069 | hsa_circ_0007694 | 23 | 14 | 3 | 12 | 16 | 4 | 15 | 4 | 9 | 108 | 68 | 14 |
| chr11:108098322\|108106561 | hsa_circ_0003641 | 26 | 22 | 10 | 10 | 19 | 5 | 29 | 21 | 33 | 206 | 100 | 37 |
| chr11:108098322\|108100050 | hsa_circ_0024193 | 26 | 26 | 11 | 8 | 23 | 12 | 36 | 47 | 19 | 392 | 148 | 60 |
| chr11:108046973\|108047817 | hsa_circ_0006208 | 84 | 58 | 28 | 62 | 59 | 33 | 12 | 18 | 14 | 378 | 132 | 76 |
| chr11:102233627\|102239279 | hsa_circ_0004103 | 12 | 10 | 6 | 10 | 8 | 11 | 22 | 34 | 16 | 164 | 66 | 37 |
| chr10:99196174\|99197507 | hsa_circ_0005887 | 22 | 19 | 5 | 24 | 41 | 12 | 49 | 45 | 28 | 247 | 159 | 47 |
| chr10:98703870\|98711953 | hsa_circ_0008102 | 23 | 23 | 3 | 20 | 12 | 3 | 22 | 33 | 18 | 121 | 67 | 32 |
| chr10:98303833\|98325183 | hsa_circ_0005801 | 7 | 9 | 4 | 2 | 6 | 4 | 8 | 6 | 4 | 46 | 20 | 8 |
| chr10:96201653\|96234540 | hsa_circ_0008874 | 16 | 7 | 5 | 6 | 7 | 2 | 5 | 20 | 10 | 104 | 23 | 8 |
| chr10:95443813\|95447206 | #N/A | 8 | 6 | 2 | 3 | 11 | 3 | 5 | 4 | 3 | 27 | 8 | 8 |
| chr10:89268093\|89280926 | #N/A | 67 | 51 | 26 | 28 | 45 | 16 | 68 | 48 | 24 | 866 | 242 | 94 |
| chr10:89266025\|89280926 | #N/A | 8 | 14 | 3 | 10 | 6 | 6 | 2 | 7 | 3 | 88 | 36 | 8 |
| chr10:88212977\|88220253 | #N/A | 11 | 8 | 4 | 7 | 6 | 4 | 8 | 15 | 14 | 89 | 34 | 16 |
| chr10:86177527\|86185649 | hsa_circ_0006956 | 24 | 20 | 6 | 8 | 17 | 6 | 17 | 9 | 6 | 99 | 75 | 16 |
| chr10:7839010\|7844817 | hsa_circ_0007292 | 26 | 30 | 11 | 14 | 14 | 11 | 23 | 29 | 18 | 235 | 167 | 40 |
| chr10:74468041\|74475660 | hsa_circ_0000246 | 9 | 10 | 2 | 8 | 7 | 4 | 13 | 16 | 4 | 44 | 28 | 16 |
| chr10:7318854\|7327916 | hsa_circ_0000211 | 14 | 9 | 5 | 14 | 12 | 11 | 8 | 6 | 2 | 79 | 50 | 17 |
| chr10:70719562\|70720005 | hsa_circ_0008865 | 40 | 36 | 22 | 26 | 24 | 9 | 29 | 22 | 11 | 286 | 122 | 44 |
| chr10:70506918\|70507323 | hsa_circ_0018553 | 6 | 7 | 2 | 8 | 6 | 6 | 14 | 2 | 4 | 61 | 31 | 7 |
| chr10:70497602\|70502326 | hsa_circ_0006272 | 44 | 32 | 16 | 36 | 60 | 17 | 50 | 38 | 22 | 285 | 125 | 34 |
| chr10:70227880\|70229920 | hsa_circ_0006151 | 38 | 31 | 7 | 22 | 22 | 2 | 28 | 43 | 20 | 276 | 137 | 61 |
| chr10:70218861\|70229920 | hsa_circ_0009172 | 8 | 8 | 4 | 11 | 6 | 5 | 9 | 7 | 10 | 122 | 46 | 20 |
| chr10:70190193\|70190417 | #N/A | 16 | 21 | 4 | 14 | 17 | 11 | 19 | 9 | 11 | 123 | 65 | 17 |
| chr10:70152895\|70154208 | hsa_circ_0000239 | 21 | 27 | 8 | 15 | 12 | 16 | 19 | 15 | 7 | 226 | 63 | 19 |
| chr10:6001717\|6002530 | hsa_circ_0007403 | 32 | 39 | 11 | 41 | 47 | 34 | 23 | 22 | 25 | 148 | 86 | 49 |
| chr10:5836848\|5842668 | hsa_circ_0002665 | 36 | 31 | 12 | 21 | 17 | 19 | 28 | 29 | 18 | 295 | 126 | 46 |
| chr10:5827105\|5842668 | hsa_circ_0005379 | 14 | 10 | 11 | 4 | 7 | 10 | 3 | 15 | 3 | 126 | 54 | 28 |
| chr10:5815805\|5842668 | hsa_circ_0017586 | 6 | 14 | 5 | 11 | 5 | 5 | 7 | 5 | 3 | 76 | 30 | 8 |
| chr10:52279591\|52350007 | hsa_circ_0018405 | 33 | 31 | 11 | 16 | 22 | 15 | 13 | 7 | 5 | 218 | 98 | 15 |
| chr10:52220433\|52350007 | hsa_circ_0018403 | 19 | 14 | 4 | 10 | 18 | 4 | 15 | 17 | 7 | 136 | 54 | 31 |
| chr10:52193236\|52350007 | hsa_circ_0018401 | 22 | 28 | 13 | 16 | 18 | 16 | 20 | 26 | 17 | 289 | 122 | 50 |
| chr10:52193236\|52226692 | #N/A | 11 | 11 | 3 | 7 | 6 | 2 | 9 | 6 | 7 | 84 | 27 | 17 |
| chr10:51584616\|51585599 | #N/A | 32 | 21 | 11 | 24 | 15 | 14 | 30 | 15 | 13 | 139 | 68 | 26 |
| chr10:50708584\|50714058 | hsa_circ_0018331 | 28 | 40 | 12 | 28 | 37 | 26 | 66 | 53 | 37 | 318 | 134 | 41 |
| chr10:49609655\|49618211 | hsa_circ_0002968 | 33 | 21 | 9 | 10 | 17 | 11 | 25 | 25 | 7 | 295 | 145 | 59 |
| chr10:46224310\|46235694 | hsa_circ_0006465 | 8 | 3 | 2 | 4 | 5 | 4 | 8 | 8 | 4 | 54 | 28 | 7 |
| chr10:46158990\|46159290 | hsa_circ_0018281 | 8 | 5 | 5 | 5 | 8 | 3 | 7 | 2 | 5 | 79 | 21 | 5 |
| chr10:46028558\|46048652 | #N/A | 10 | 3 | 5 | 10 | 13 | 7 | 18 | 9 | 7 | 86 | 46 | 10 |
| chr10:35805451\|35819171 | hsa_circ_0000235 | 8 | 14 | 6 | 3 | 4 | 8 | 12 | 10 | 5 | 85 | 41 | 12 |
| chr10:35349802\|35360267 | hsa_circ_0000234 | 5 | 9 | 7 | 7 | 6 | 2 | 3 | 16 | 4 | 56 | 31 | 14 |
| chr10:32854486\|32873232 | hsa_circ_0008679 | 36 | 26 | 17 | 6 | 22 | 11 | 6 | 7 | 12 | 249 | 76 | 29 |
| chr10:32832228\|32873232 | hsa_circ_0000233 | 34 | 33 | 15 | 8 | 13 | 16 | 3 | 3 | 8 | 147 | 21 | 11 |
| chr10:32759992\|32762951 | hsa_circ_0002351 | 13 | 25 | 5 | 6 | 14 | 10 | 14 | 22 | 15 | 115 | 77 | 14 |
| chr10:32562091\|32562966 | #N/A | 7 | 10 | 3 | 7 | 5 | 7 | 3 | 8 | 2 | 42 | 28 | 10 |
| chr10:32308786\|32310215 | hsa_circ_0006408 | 25 | 24 | 7 | 23 | 17 | 15 | 20 | 16 | 12 | 132 | 52 | 23 |
| chr10:32197100\|32199491 | hsa_circ_0000231 | 7 | 6 | 6 | 4 | 7 | 2 | 8 | 15 | 5 | 78 | 31 | 16 |
| chr10:3201106\|3202522 | hsa_circ_0017510 | 8 | 19 | 2 | 18 | 15 | 8 | 17 | 16 | 11 | 180 | 92 | 20 |
| chr10:31749966\|31750166 | hsa_circ_0002140 | 20 | 14 | 4 | 21 | 24 | 16 | 18 | 17 | 14 | 180 | 86 | 10 |
| chr10:31661947\|31676195 | hsa_circ_0000228 | 24 | 17 | 6 | 18 | 20 | 5 | 32 | 28 | 13 | 271 | 112 | 42 |
| chr10:28872328\|28884970 | hsa_circ_0007503 | 13 | 16 | 10 | 22 | 12 | 7 | 14 | 17 | 5 | 104 | 47 | 11 |
| chr10:28872328\|28879761 | hsa_circ_0018054 | 12 | 5 | 4 | 5 | 9 | 5 | 10 | 11 | 5 | 111 | 29 | 21 |
| chr10:27821436\|27822923 | hsa_circ_0000226 | 23 | 17 | 6 | 14 | 13 | 12 | 16 | 15 | 13 | 151 | 43 | 23 |
| chr10:27453993\|27454468 | hsa_circ_0000225 | 40 | 43 | 16 | 16 | 33 | 24 | 24 | 21 | 11 | 205 | 98 | 32 |
| chr10:27431316\|27434519 | hsa_circ_0005633 | 42 | 49 | 16 | 47 | 42 | 22 | 21 | 15 | 15 | 362 | 126 | 39 |
| chr10:27047991\|27059274 | #N/A | 19 | 20 | 5 | 17 | 15 | 12 | 15 | 10 | 13 | 116 | 51 | 21 |
| chr10:26789748\|26792203 | hsa_circ_0006063 | 13 | 11 | 2 | 15 | 8 | 7 | 22 | 17 | 17 | 111 | 54 | 22 |
| chr10:22880558\|22898646 | hsa_circ_0003583 | 58 | 52 | 12 | 53 | 64 | 35 | 67 | 75 | 41 | 562 | 239 | 79 |
| chr10:22606813\|22607947 | #N/A | 11 | 8 | 4 | 10 | 5 | 3 | 12 | 8 | 2 | 87 | 61 | 15 |
| chr10:22002701\|22016857 | hsa_circ_0007084 | 12 | 15 | 12 | 18 | 14 | 5 | 32 | 37 | 15 | 183 | 109 | 31 |
| chr10:17746430\|17747740 | hsa_circ_0008311 | 45 | 53 | 26 | 25 | 40 | 28 | 38 | 34 | 18 | 358 | 147 | 31 |
| chr10:16794538\|16824083 | hsa_circ_0006577 | 55 | 39 | 22 | 39 | 39 | 23 | 57 | 88 | 36 | 487 | 258 | 73 |
| chr10:16794538\|16806509 | hsa_circ_0017856 | 13 | 17 | 4 | 15 | 11 | 5 | 32 | 32 | 13 | 116 | 93 | 36 |
| chr10:15875629\|15889942 | hsa_circ_0006665 | 19 | 13 | 5 | 13 | 13 | 10 | 18 | 20 | 10 | 151 | 77 | 26 |
| chr10:15858834\|15889942 | hsa_circ_0000219 | 14 | 13 | 6 | 7 | 8 | 8 | 6 | 19 | 11 | 143 | 60 | 10 |
| chr10:13233299\|13234568 | hsa_circ_0004477 | 29 | 24 | 10 | 16 | 21 | 11 | 24 | 49 | 20 | 258 | 132 | 42 |
| chr10:13169745\|13178897 | hsa_circ_0007242 | 20 | 18 | 6 | 14 | 15 | 10 | 23 | 52 | 22 | 211 | 122 | 58 |
| chr10:126631026\|126631876 | hsa_circ_0000268 | 59 | 36 | 17 | 19 | 22 | 13 | 46 | 67 | 30 | 408 | 210 | 74 |
| chr10:126370176\|126370948 | hsa_circ_0000267 | 27 | 19 | 9 | 19 | 23 | 5 | 30 | 27 | 21 | 154 | 143 | 52 |
| chr10:126097111\|126100769 | hsa_circ_0008898 | 19 | 24 | 3 | 11 | 14 | 8 | 25 | 25 | 8 | 231 | 90 | 40 |
| chr10:121275021\|121286936 | hsa_circ_0005516 | 41 | 40 | 9 | 30 | 36 | 11 | 40 | 51 | 34 | 325 | 159 | 77 |
| chr10:120797750\|120797951 | hsa_circ_0004792 | 8 | 7 | 2 | 10 | 3 | 6 | 2 | 3 | 6 | 26 | 16 | 7 |
| chr10:12039671\|12056183 | hsa_circ_0000213 | 12 | 9 | 8 | 5 | 5 | 3 | 4 | 10 | 7 | 82 | 32 | 14 |
| chr10:11643344\|11643979 | hsa_circ_0006664 | 19 | 23 | 9 | 9 | 21 | 10 | 4 | 14 | 18 | 176 | 94 | 39 |
| chr10:11639630\|11643979 | hsa_circ_0003978 | 94 | 62 | 32 | 52 | 89 | 28 | 90 | 97 | 58 | 879 | 372 | 118 |
| chr10:115636280\|115644139 | #N/A | 10 | 5 | 2 | 5 | 2 | 6 | 9 | 11 | 6 | 99 | 30 | 16 |
| chr10:11523769\|11527910 | hsa_circ_0017683 | 20 | 7 | 2 | 15 | 19 | 3 | 19 | 33 | 7 | 208 | 64 | 22 |
| chr10:114220283\|114224416 | hsa_circ_0020045 | 8 | 2 | 7 | 8 | 5 | 4 | 4 | 4 | 7 | 80 | 37 | 20 |
| chr10:112356156\|112358048 | hsa_circ_0000260 | 32 | 21 | 18 | 17 | 13 | 11 | 33 | 33 | 20 | 236 | 103 | 38 |
| chr10:111883775\|111890244 | hsa_circ_0003357 | 15 | 19 | 3 | 3 | 3 | 7 | 4 | 16 | 15 | 101 | 54 | 17 |
| chr10:105767935\|105778666 | hsa_circ_0000259 | 68 | 40 | 25 | 32 | 27 | 24 | 30 | 46 | 17 | 385 | 163 | 52 |
| chr10:105197772\|105198565 | hsa_circ_0000258 | 80 | 81 | 18 | 77 | 56 | 34 | 26 | 32 | 19 | 332 | 235 | 73 |
| chr10:105106995\|105108756 | hsa_circ_0005052 | 15 | 11 | 5 | 9 | 14 | 5 | 4 | 9 | 4 | 149 | 53 | 18 |
| chr10:103427643\|103436193 | hsa_circ_0008362 | 15 | 20 | 2 | 18 | 23 | 8 | 32 | 35 | 34 | 175 | 127 | 47 |
| chr1:9991949\|9995685 | hsa_circ_0007803 | 12 | 8 | 9 | 3 | 12 | 2 | 34 | 23 | 7 | 109 | 52 | 23 |
| chr1:9991949\|9994918 | hsa_circ_0000014 | 87 | 85 | 26 | 92 | 127 | 50 | 93 | 100 | 71 | 916 | 470 | 156 |
| chr1:95609447\|95639445 | hsa_circ_0005720 | 111 | 106 | 37 | 58 | 71 | 42 | 133 | 234 | 123 | 1423 | 715 | 305 |
| chr1:95609447\|95618136 | #N/A | 9 | 5 | 2 | 4 | 3 | 3 | 5 | 11 | 4 | 75 | 16 | 12 |
| chr1:95609447\|95616975 | hsa_circ_0000095 | 970 | 744 | 278 | 671 | 858 | 411 | 1384 | 1801 | 1013 | 13087 | 5470 | 2516 |
| chr1:95606229\|95616975 | #N/A | 11 | 15 | 15 | 17 | 20 | 8 | 29 | 37 | 14 | 211 | 131 | 39 |
| chr1:95603831\|95639445 | #N/A | 11 | 17 | 10 | 6 | 23 | 7 | 36 | 38 | 12 | 168 | 90 | 32 |
| chr1:95603831\|95616975 | #N/A | 25 | 13 | 6 | 14 | 20 | 9 | 28 | 35 | 15 | 222 | 95 | 43 |
| chr1:94360170\|94363454 | hsa_circ_0013222 | 21 | 18 | 5 | 19 | 29 | 6 | 36 | 55 | 22 | 243 | 137 | 65 |
| chr1:93790192\|93791452 | hsa_circ_0013204 | 7 | 5 | 2 | 5 | 6 | 3 | 3 | 5 | 6 | 43 | 21 | 8 |
| chr1:92798948\|92846430 | hsa_circ_0000091 | 9 | 23 | 7 | 17 | 17 | 9 | 23 | 17 | 15 | 208 | 62 | 30 |
| chr1:89236035\|89237562 | hsa_circ_0002008 | 75 | 65 | 33 | 44 | 50 | 22 | 56 | 75 | 56 | 584 | 317 | 104 |
| chr1:87185190\|87190088 | hsa_circ_0013084 | 6 | 10 | 5 | 5 | 5 | 2 | 6 | 5 | 3 | 65 | 21 | 12 |
| chr1:87181407\|87185318 | hsa_circ_0013081 | 3 | 10 | 2 | 5 | 6 | 4 | 8 | 8 | 4 | 67 | 19 | 11 |
| chr1:8716032\|8716500 | #N/A | 14 | 12 | 11 | 16 | 8 | 5 | 2 | 11 | 3 | 44 | 19 | 9 |
| chr1:8601273\|8674745 | hsa_circ_0008501 | 15 | 7 | 2 | 9 | 8 | 8 | 9 | 4 | 7 | 78 | 50 | 16 |
| chr1:8601273\|8617582 | hsa_circ_0002158 | 13 | 7 | 6 | 19 | 12 | 8 | 6 | 17 | 3 | 174 | 57 | 26 |
| chr1:7837220\|7838229 | hsa_circ_0006354 | 48 | 63 | 33 | 77 | 63 | 48 | 42 | 29 | 32 | 399 | 274 | 88 |
| chr1:78177432\|78181553 | hsa_circ_0005374 | 36 | 39 | 14 | 28 | 25 | 14 | 31 | 26 | 23 | 498 | 164 | 68 |
| chr1:78177432\|78180468 | #N/A | 21 | 19 | 4 | 17 | 12 | 3 | 20 | 27 | 11 | 227 | 72 | 28 |
| chr1:76198329\|76200556 | hsa_circ_0012963 | 8 | 3 | 2 | 9 | 3 | 2 | 16 | 15 | 9 | 125 | 43 | 6 |
| chr1:741179\|745550 | hsa_circ_0002333 | 42 | 26 | 11 | 16 | 14 | 20 | 5 | 13 | 10 | 262 | 75 | 19 |
| chr1:70758071\|70781249 | hsa_circ_0000085 | 33 | 24 | 8 | 10 | 17 | 10 | 40 | 33 | 13 | 308 | 120 | 47 |
| chr1:67356837\|67371058 | hsa_circ_0006677 | 23 | 21 | 8 | 20 | 21 | 10 | 24 | 32 | 11 | 287 | 139 | 40 |
| chr1:65830318\|65831879 | hsa_circ_0002454 | 974 | 849 | 269 | 678 | 868 | 314 | 1244 | 1438 | 868 | 9844 | 4728 | 1919 |
| chr1:63944435\|63974241 | hsa_circ_0004586 | 45 | 45 | 16 | 25 | 29 | 17 | 19 | 20 | 11 | 401 | 179 | 52 |
| chr1:63944435\|63955889 | #N/A | 23 | 22 | 5 | 16 | 30 | 16 | 8 | 4 | 5 | 173 | 79 | 10 |
| chr1:61624547\|61624827 | #N/A | 2 | 7 | 3 | 3 | 5 | 12 | 5 | 4 | 7 | 63 | 26 | 6 |
| chr1:61577043\|61624827 | #N/A | 24 | 8 | 3 | 12 | 10 | 10 | 22 | 19 | 5 | 193 | 64 | 27 |
| chr1:51871577\|51874004 | hsa_circ_0004877 | 39 | 30 | 7 | 21 | 22 | 7 | 32 | 59 | 21 | 231 | 174 | 58 |
| chr1:51868107\|51874004 | hsa_circ_0005567 | 48 | 28 | 7 | 16 | 28 | 15 | 43 | 40 | 24 | 307 | 161 | 70 |
| chr1:51121114\|51210447 | hsa_circ_0004619 | 31 | 24 | 8 | 17 | 18 | 9 | 33 | 31 | 17 | 272 | 125 | 58 |
| chr1:50956260\|51001129 | hsa_circ_0008981 | 28 | 26 | 8 | 14 | 14 | 8 | 23 | 21 | 21 | 115 | 69 | 22 |
| chr1:47834141\|47840965 | hsa_circ_0008201 | 17 | 19 | 4 | 9 | 8 | 5 | 12 | 20 | 6 | 126 | 52 | 29 |
| chr1:47753223\|47765824 | hsa_circ_0004280 | 12 | 6 | 3 | 12 | 4 | 3 | 7 | 16 | 8 | 92 | 47 | 20 |
| chr1:47753223\|47761520 | hsa_circ_0012378 | 9 | 9 | 2 | 3 | 7 | 6 | 19 | 12 | 4 | 144 | 40 | 15 |
| chr1:47745913\|47748131 | hsa_circ_0000069 | 90 | 58 | 13 | 36 | 37 | 23 | 26 | 27 | 27 | 332 | 142 | 61 |
| chr1:47638521\|47644121 | #N/A | 10 | 10 | 2 | 8 | 3 | 2 | 4 | 13 | 12 | 61 | 44 | 18 |
| chr1:46105882\|46108171 | hsa_circ_0008774 | 173 | 180 | 40 | 230 | 248 | 142 | 186 | 136 | 146 | 1205 | 630 | 211 |
| chr1:43300709\|43304592 | #N/A | 6 | 17 | 8 | 19 | 20 | 12 | 9 | 7 | 7 | 74 | 45 | 17 |
| chr1:42744019\|42744343 | #N/A | 6 | 5 | 2 | 3 | 6 | 8 | 4 | 5 | 6 | 46 | 24 | 6 |
| chr1:42730786\|42744455 | #N/A | 15 | 10 | 6 | 9 | 9 | 4 | 22 | 24 | 10 | 154 | 69 | 32 |
| chr1:42730786\|42744343 | hsa_circ_0006848 | 48 | 15 | 8 | 18 | 17 | 11 | 25 | 33 | 22 | 286 | 120 | 52 |
| chr1:41536267\|41541123 | hsa_circ_0000061 | 42 | 46 | 14 | 73 | 97 | 30 | 74 | 92 | 42 | 366 | 240 | 69 |
| chr1:35824526\|35827390 | hsa_circ_0011536 | 24 | 16 | 3 | 4 | 5 | 8 | 8 | 3 | 2 | 63 | 29 | 9 |
| chr1:32381496\|32385259 | hsa_circ_0007364 | 11 | 9 | 8 | 7 | 9 | 7 | 12 | 8 | 7 | 72 | 45 | 15 |
| chr1:32375639\|32377427 | hsa_circ_0005674 | 11 | 5 | 2 | 7 | 11 | 2 | 6 | 5 | 6 | 87 | 40 | 9 |
| chr1:31810022\|31811895 | hsa_circ_0009057 | 37 | 23 | 12 | 19 | 23 | 14 | 25 | 14 | 10 | 271 | 134 | 24 |
| chr1:31465237\|31468067 | hsa_circ_0000043 | 6 | 13 | 4 | 10 | 8 | 11 | 21 | 22 | 19 | 138 | 72 | 25 |
| chr1:29481208\|29481422 | hsa_circ_0006602 | 14 | 27 | 6 | 26 | 19 | 12 | 17 | 16 | 10 | 131 | 77 | 29 |
| chr1:29386934\|29424447 | #N/A | 72 | 65 | 18 | 62 | 70 | 25 | 70 | 76 | 54 | 641 | 290 | 89 |
| chr1:29362338\|29391670 | hsa_circ_0011174 | 33 | 25 | 9 | 16 | 20 | 7 | 34 | 29 | 17 | 239 | 116 | 32 |
| chr1:29362338\|29379824 | hsa_circ_0011173 | 25 | 16 | 3 | 21 | 10 | 5 | 22 | 28 | 15 | 138 | 105 | 33 |
| chr1:29362338\|29365938 | hsa_circ_0000042 | 131 | 122 | 26 | 121 | 144 | 57 | 149 | 128 | 100 | 877 | 511 | 153 |
| chr1:29319842\|29344954 | hsa_circ_0000041 | 6 | 6 | 3 | 2 | 3 | 8 | 11 | 6 | 2 | 68 | 6 | 6 |
| chr1:29319842\|29323831 | hsa_circ_0011172 | 44 | 46 | 13 | 26 | 46 | 21 | 58 | 60 | 36 | 462 | 188 | 68 |
| chr1:29313943\|29314417 | hsa_circ_0011167 | 170 | 145 | 72 | 101 | 127 | 58 | 147 | 197 | 117 | 1535 | 740 | 222 |
| chr1:28907072\|28907741 | hsa_circ_0005174 | 22 | 11 | 8 | 17 | 19 | 7 | 11 | 20 | 12 | 68 | 61 | 8 |
| chr1:28897688\|28898412 | hsa_circ_0011156 | 10 | 8 | 2 | 6 | 4 | 6 | 4 | 5 | 4 | 59 | 40 | 6 |
| chr1:28800066\|28802803 | hsa_circ_0000038 | 12 | 10 | 6 | 13 | 12 | 9 | 5 | 6 | 3 | 46 | 30 | 4 |
| chr1:28800066\|28800663 | hsa_circ_0002909 | 16 | 6 | 5 | 17 | 16 | 4 | 14 | 5 | 2 | 53 | 28 | 17 |
| chr1:28362055\|28384605 | hsa_circ_0007895 | 25 | 13 | 11 | 18 | 32 | 14 | 11 | 35 | 33 | 251 | 144 | 43 |
| chr1:28362055\|28374937 | hsa_circ_0008057 | 13 | 23 | 6 | 29 | 24 | 7 | 23 | 26 | 12 | 159 | 69 | 23 |
| chr1:27267948\|27268309 | hsa_circ_0003940 | 5 | 8 | 2 | 11 | 14 | 9 | 13 | 15 | 10 | 54 | 37 | 6 |
| chr1:27056142\|27059283 | hsa_circ_0008494 | 5 | 8 | 2 | 33 | 49 | 23 | 5 | 11 | 6 | 4 | 5 | 3 |
| chr1:26594974\|26596105 | #N/A | 25 | 33 | 5 | 16 | 24 | 21 | 47 | 42 | 35 | 224 | 133 | 43 |
| chr1:25666965\|25687234 | #N/A | 7 | 8 | 2 | 5 | 6 | 2 | 11 | 10 | 8 | 67 | 22 | 5 |
| chr1:25666965\|25683344 | #N/A | 25 | 14 | 5 | 9 | 9 | 12 | 22 | 25 | 11 | 215 | 99 | 40 |
| chr1:25627437\|25628177 | #N/A | 11 | 4 | 5 | 15 | 6 | 3 | 2 | 13 | 9 | 39 | 29 | 10 |
| chr1:25553933\|25554726 | #N/A | 25 | 28 | 11 | 13 | 9 | 21 | 12 | 9 | 8 | 65 | 37 | 12 |
| chr1:24840804\|24841057 | hsa_circ_0003553 | 20 | 17 | 9 | 20 | 18 | 15 | 7 | 12 | 11 | 145 | 65 | 37 |
| chr1:248023919\|248031365 | #N/A | 22 | 14 | 9 | 20 | 18 | 8 | 17 | 53 | 25 | 123 | 109 | 33 |
| chr1:247318868\|247323115 | hsa_circ_0002922 | 6 | 16 | 3 | 4 | 4 | 3 | 10 | 10 | 6 | 68 | 26 | 24 |
| chr1:246754814\|246755243 | hsa_circ_0017310 | 14 | 15 | 7 | 9 | 25 | 6 | 24 | 45 | 26 | 207 | 117 | 51 |
| chr1:245165423\|245180628 | hsa_circ_0008294 | 8 | 9 | 3 | 12 | 7 | 7 | 5 | 8 | 5 | 59 | 28 | 17 |
| chr1:243579004\|243589860 | hsa_circ_0017241 | 10 | 9 | 5 | 4 | 3 | 2 | 7 | 17 | 5 | 107 | 46 | 18 |
| chr1:243456393\|243471479 | hsa_circ_0007442 | 5 | 5 | 4 | 5 | 8 | 5 | 5 | 10 | 5 | 67 | 38 | 14 |
| chr1:235963620\|235964397 | hsa_circ_0017092 | 3 | 10 | 2 | 6 | 13 | 2 | 3 | 3 | 2 | 72 | 27 | 9 |
| chr1:23397718\|23398690 | hsa_circ_0010835 | 25 | 26 | 7 | 20 | 18 | 8 | 9 | 18 | 10 | 126 | 77 | 25 |
| chr1:23356962\|23377013 | hsa_circ_0009061 | 28 | 28 | 11 | 21 | 28 | 14 | 43 | 68 | 11 | 263 | 149 | 62 |
| chr1:231090079\|231097049 | hsa_circ_0000195 | 61 | 80 | 24 | 60 | 52 | 26 | 66 | 49 | 45 | 596 | 284 | 102 |
| chr1:230798887\|230800333 | hsa_circ_0016866 | 14 | 20 | 4 | 16 | 18 | 6 | 6 | 4 | 3 | 101 | 44 | 20 |
| chr1:227381487\|227504947 | #N/A | 21 | 17 | 7 | 7 | 13 | 7 | 11 | 4 | 3 | 124 | 32 | 11 |
| chr1:226453234\|226454033 | hsa_circ_0016707 | 12 | 13 | 3 | 9 | 14 | 19 | 9 | 3 | 7 | 51 | 42 | 13 |
| chr1:226175597\|226176089 | #N/A | 11 | 7 | 2 | 2 | 3 | 3 | 3 | 4 | 5 | 45 | 20 | 5 |
| chr1:224599129\|224601037 | hsa_circ_0005242 | 21 | 21 | 3 | 11 | 15 | 8 | 14 | 11 | 8 | 148 | 64 | 35 |
| chr1:222897434\|222898897 | hsa_circ_0003413 | 66 | 40 | 11 | 30 | 38 | 30 | 16 | 8 | 7 | 370 | 148 | 32 |
| chr1:22047529\|22048257 | hsa_circ_0004808 | 21 | 33 | 6 | 4 | 13 | 11 | 5 | 12 | 7 | 58 | 32 | 16 |
| chr1:22041882\|22048257 | hsa_circ_0000028 | 49 | 38 | 20 | 13 | 12 | 11 | 25 | 12 | 10 | 214 | 59 | 14 |
| chr1:220179448\|220180680 | hsa_circ_0007739 | 26 | 28 | 19 | 19 | 29 | 3 | 26 | 36 | 23 | 355 | 118 | 42 |
| chr1:21329206\|21437876 | hsa_circ_0007227 | 12 | 21 | 5 | 10 | 16 | 7 | 13 | 18 | 12 | 100 | 54 | 17 |
| chr1:213251038\|213290752 | hsa_circ_0004849 | 21 | 16 | 5 | 9 | 19 | 6 | 23 | 21 | 15 | 170 | 62 | 16 |
| chr1:213037067\|213058738 | hsa_circ_0000182 | 11 | 10 | 2 | 3 | 6 | 5 | 14 | 13 | 9 | 108 | 57 | 7 |
| chr1:212218002\|212220759 | hsa_circ_0000179 | 64 | 68 | 19 | 45 | 64 | 26 | 71 | 78 | 43 | 600 | 289 | 139 |
| chr1:21102217\|21102498 | #N/A | 172 | 168 | 48 | 103 | 122 | 61 | 99 | 102 | 75 | 1280 | 603 | 210 |
| chr1:21097423\|21100103 | hsa_circ_0005782 | 125 | 86 | 36 | 80 | 91 | 50 | 104 | 130 | 88 | 890 | 393 | 187 |
| chr1:21091870\|21100103 | hsa_circ_0002238 | 32 | 24 | 8 | 18 | 28 | 8 | 23 | 33 | 11 | 240 | 130 | 24 |
| chr1:21083659\|21100103 | hsa_circ_0000024 | 184 | 133 | 91 | 118 | 129 | 55 | 197 | 204 | 124 | 1423 | 734 | 243 |
| chr1:21076216\|21100103 | hsa_circ_0000023 | 54 | 44 | 22 | 28 | 29 | 9 | 35 | 38 | 25 | 337 | 143 | 44 |
| chr1:207896963\|207911916 | #N/A | 7 | 10 | 4 | 7 | 9 | 5 | 7 | 2 | 3 | 78 | 37 | 12 |
| chr1:207850734\|207857302 | #N/A | 21 | 18 | 7 | 18 | 16 | 8 | 30 | 39 | 20 | 216 | 137 | 32 |
| chr1:207820662\|207851642 | #N/A | 7 | 10 | 5 | 3 | 7 | 2 | 12 | 18 | 6 | 100 | 50 | 20 |
| chr1:207820662\|207828620 | #N/A | 16 | 16 | 3 | 20 | 14 | 12 | 6 | 7 | 5 | 95 | 64 | 7 |
| chr1:207820263\|207828620 | #N/A | 22 | 11 | 6 | 18 | 19 | 10 | 16 | 7 | 10 | 109 | 75 | 16 |
| chr1:205238078\|205239012 | hsa_circ_0003344 | 49 | 53 | 16 | 32 | 43 | 27 | 64 | 66 | 27 | 241 | 248 | 54 |
| chr1:200583446\|200584737 | hsa_circ_0004522 | 13 | 5 | 3 | 4 | 5 | 5 | 7 | 10 | 7 | 91 | 36 | 9 |
| chr1:200550329\|200561368 | #N/A | 16 | 12 | 2 | 9 | 8 | 2 | 5 | 3 | 3 | 60 | 29 | 7 |
| chr1:198201683\|198233365 | hsa_circ_0002528 | 5 | 8 | 2 | 10 | 9 | 2 | 9 | 8 | 6 | 67 | 31 | 7 |
| chr1:197509094\|197564463 | #N/A | 13 | 21 | 4 | 4 | 7 | 5 | 2 | 15 | 4 | 76 | 39 | 14 |
| chr1:193044950\|193046180 | #N/A | 13 | 10 | 8 | 6 | 9 | 4 | 5 | 4 | 6 | 124 | 33 | 17 |
| chr1:187296053\|187298192 | #N/A | 14 | 17 | 8 | 28 | 18 | 16 | 7 | 6 | 3 | 171 | 71 | 21 |
| chr1:187293225\|187298192 | #N/A | 5 | 6 | 5 | 6 | 3 | 4 | 3 | 6 | 3 | 45 | 17 | 3 |
| chr1:187272598\|187298192 | #N/A | 42 | 43 | 25 | 35 | 38 | 18 | 36 | 46 | 28 | 339 | 156 | 33 |
| chr1:187061957\|187298192 | #N/A | 9 | 3 | 3 | 7 | 15 | 5 | 7 | 9 | 5 | 38 | 24 | 9 |
| chr1:185183639\|185200840 | hsa_circ_0004689 | 483 | 360 | 141 | 228 | 289 | 173 | 458 | 555 | 255 | 5142 | 1978 | 733 |
| chr1:185149523\|185154063 | #N/A | 5 | 4 | 4 | 4 | 2 | 5 | 3 | 8 | 8 | 79 | 21 | 17 |
| chr1:185056684\|185062408 | hsa_circ_0002829 | 10 | 10 | 9 | 7 | 7 | 6 | 5 | 9 | 5 | 80 | 48 | 14 |
| chr1:180953813\|180962561 | hsa_circ_0007905 | 27 | 17 | 4 | 15 | 13 | 9 | 14 | 49 | 28 | 220 | 116 | 40 |
| chr1:180031355\|180034407 | hsa_circ_0015489 | 11 | 13 | 8 | 5 | 4 | 3 | 16 | 13 | 7 | 81 | 30 | 16 |
| chr1:179972309\|179975702 | hsa_circ_0003942 | 38 | 32 | 9 | 21 | 26 | 14 | 31 | 29 | 18 | 235 | 94 | 58 |
| chr1:1756836\|1770677 | hsa_circ_0009362 | 20 | 30 | 8 | 19 | 27 | 8 | 7 | 10 | 5 | 269 | 113 | 24 |
| chr1:174938414\|174952042 | #N/A | 29 | 26 | 2 | 17 | 22 | 14 | 25 | 25 | 12 | 140 | 74 | 27 |
| chr1:1747195\|1770677 | hsa_circ_0008702 | 41 | 43 | 9 | 40 | 43 | 7 | 40 | 53 | 48 | 368 | 213 | 75 |
| chr1:1747195\|1756938 | hsa_circ_0000008 | 32 | 36 | 13 | 24 | 17 | 5 | 25 | 24 | 23 | 149 | 105 | 35 |
| chr1:174188639\|174189592 | #N/A | 7 | 7 | 6 | 16 | 6 | 6 | 12 | 4 | 3 | 89 | 29 | 5 |
| chr1:174188263\|174190302 | hsa_circ_0008141 | 11 | 6 | 3 | 3 | 4 | 7 | 4 | 4 | 9 | 57 | 26 | 17 |
| chr1:172525009\|172526934 | hsa_circ_0015262 | 71 | 51 | 17 | 43 | 49 | 35 | 54 | 73 | 44 | 756 | 350 | 126 |
| chr1:172520652\|172526934 | hsa_circ_0000160 | 144 | 105 | 48 | 91 | 97 | 58 | 111 | 125 | 71 | 1129 | 564 | 208 |
| chr1:171537386\|171544267 | hsa_circ_0004412 | 32 | 19 | 12 | 35 | 19 | 10 | 45 | 46 | 39 | 243 | 171 | 46 |
| chr1:171492360\|171496983 | hsa_circ_0003644 | 9 | 6 | 2 | 4 | 10 | 2 | 12 | 13 | 7 | 63 | 45 | 18 |
| chr1:168007609\|168014465 | hsa_circ_0000155 | 43 | 27 | 13 | 15 | 22 | 7 | 14 | 28 | 13 | 230 | 125 | 35 |
| chr1:167971720\|167974031 | hsa_circ_0015124 | 9 | 2 | 7 | 9 | 5 | 4 | 7 | 20 | 5 | 85 | 60 | 12 |
| chr1:167935867\|167944253 | hsa_circ_0009109 | 37 | 35 | 22 | 31 | 38 | 21 | 38 | 29 | 8 | 417 | 199 | 49 |
| chr1:167921038\|167944253 | hsa_circ_0000154 | 34 | 38 | 8 | 16 | 25 | 13 | 37 | 27 | 17 | 334 | 123 | 50 |
| chr1:167301707\|167307495 | #N/A | 18 | 21 | 9 | 20 | 33 | 14 | 42 | 30 | 22 | 237 | 145 | 52 |
| chr1:1647590\|1647917 | #N/A | 12 | 11 | 10 | 13 | 13 | 9 | 13 | 9 | 10 | 49 | 39 | 8 |
| chr1:161748034\|161753886 | hsa_circ_0003039 | 6 | 3 | 4 | 5 | 7 | 4 | 4 | 6 | 3 | 21 | 7 | 10 |
| chr1:16044388\|16047883 | hsa_circ_0000021 | 18 | 23 | 4 | 13 | 7 | 4 | 26 | 8 | 20 | 120 | 96 | 23 |
| chr1:160293221\|160302347 | hsa_circ_0008661 | 31 | 18 | 4 | 23 | 22 | 12 | 29 | 58 | 38 | 243 | 138 | 38 |
| chr1:15964802\|15978390 | hsa_circ_0000020 | 12 | 11 | 3 | 7 | 7 | 6 | 21 | 21 | 11 | 119 | 53 | 33 |
| chr1:15964802\|15970145 | hsa_circ_0000019 | 268 | 207 | 81 | 193 | 185 | 133 | 240 | 278 | 196 | 1637 | 884 | 303 |
| chr1:15956820\|15978390 | hsa_circ_0010052 | 21 | 12 | 4 | 4 | 4 | 2 | 12 | 18 | 5 | 71 | 52 | 19 |
| chr1:158624601\|158624741 | #N/A | 105 | 94 | 24 | 98 | 132 | 63 | 18 | 30 | 19 | 1021 | 516 | 97 |
| chr1:15860732\|15863309 | hsa_circ_0000018 | 28 | 20 | 15 | 18 | 21 | 17 | 23 | 41 | 20 | 321 | 137 | 54 |
| chr1:1584361\|1647917 | #N/A | 20 | 12 | 4 | 13 | 22 | 9 | 17 | 12 | 14 | 45 | 38 | 9 |
| chr1:156303338\|156304709 | hsa_circ_0004680 | 58 | 71 | 15 | 44 | 61 | 28 | 21 | 22 | 16 | 294 | 247 | 72 |
| chr1:155896467\|155896947 | hsa_circ_0008339 | 27 | 18 | 8 | 18 | 18 | 17 | 9 | 10 | 15 | 84 | 51 | 13 |
| chr1:155891166\|155891709 | hsa_circ_0008998 | 19 | 11 | 5 | 21 | 20 | 8 | 13 | 22 | 10 | 113 | 72 | 17 |
| chr1:155874102\|155874595 | hsa_circ_0006575 | 12 | 9 | 7 | 12 | 13 | 5 | 13 | 16 | 9 | 80 | 77 | 37 |
| chr1:155823067\|155823597 | hsa_circ_0000139 | 45 | 27 | 11 | 11 | 24 | 12 | 26 | 50 | 26 | 312 | 153 | 60 |
| chr1:155737216\|155743001 | #N/A | 3 | 3 | 2 | 4 | 3 | 2 | 3 | 8 | 4 | 43 | 17 | 4 |
| chr1:155691308\|155695810 | hsa_circ_0014613 | 27 | 26 | 5 | 26 | 25 | 14 | 44 | 30 | 23 | 267 | 143 | 43 |
| chr1:155646339\|155753880 | #N/A | 95 | 71 | 18 | 64 | 66 | 24 | 89 | 127 | 71 | 598 | 374 | 161 |
| chr1:155646339\|155650247 | #N/A | 15 | 8 | 5 | 14 | 18 | 4 | 18 | 29 | 27 | 116 | 73 | 24 |
| chr1:155646339\|155649303 | hsa_circ_0014606 | 573 | 543 | 157 | 388 | 439 | 181 | 595 | 857 | 412 | 4057 | 2402 | 923 |
| chr1:155644801\|155753880 | hsa_circ_0002816 | 21 | 20 | 7 | 23 | 16 | 7 | 25 | 26 | 25 | 186 | 114 | 43 |
| chr1:155644801\|155650247 | #N/A | 14 | 21 | 10 | 8 | 5 | 8 | 10 | 30 | 14 | 113 | 93 | 40 |
| chr1:155644801\|155649303 | hsa_circ_0003608 | 98 | 91 | 35 | 81 | 103 | 51 | 131 | 205 | 100 | 1034 | 671 | 194 |
| chr1:155640111\|155649303 | #N/A | 4 | 13 | 8 | 6 | 11 | 4 | 7 | 14 | 9 | 83 | 52 | 9 |
| chr1:155638418\|155644887 | hsa_circ_0014601 | 6 | 4 | 2 | 7 | 3 | 4 | 6 | 8 | 8 | 42 | 16 | 14 |
| chr1:155490891\|155491409 | hsa_circ_0014592 | 3 | 5 | 3 | 2 | 4 | 3 | 3 | 2 | 2 | 46 | 15 | 6 |
| chr1:155408118\|155429689 | hsa_circ_0003247 | 38 | 30 | 10 | 15 | 19 | 2 | 19 | 25 | 21 | 210 | 106 | 28 |
| chr1:155408118\|155408859 | hsa_circ_0000137 | 21 | 18 | 12 | 14 | 6 | 7 | 25 | 38 | 18 | 155 | 59 | 37 |
| chr1:155340295\|155340774 | hsa_circ_0007608 | 3 | 5 | 2 | 4 | 5 | 6 | 7 | 8 | 5 | 41 | 19 | 2 |
| chr1:154223517\|154224129 | hsa_circ_0004717 | 9 | 15 | 6 | 7 | 10 | 6 | 11 | 14 | 12 | 108 | 40 | 20 |
| chr1:154207067\|154207767 | #N/A | 6 | 11 | 6 | 19 | 12 | 18 | 19 | 16 | 14 | 32 | 27 | 10 |
| chr1:154145384\|154145677 | hsa_circ_0008368 | 21 | 25 | 10 | 34 | 47 | 19 | 10 | 23 | 14 | 162 | 94 | 17 |
| chr1:151139410\|151139890 | hsa_circ_0000128 | 66 | 65 | 29 | 49 | 76 | 40 | 60 | 65 | 41 | 517 | 295 | 119 |
| chr1:150997087\|150998149 | hsa_circ_0006760 | 24 | 19 | 5 | 11 | 15 | 10 | 36 | 14 | 15 | 213 | 131 | 29 |
| chr1:145594039\|145595220 | hsa_circ_0003693 | 17 | 12 | 2 | 17 | 18 | 8 | 20 | 22 | 8 | 134 | 72 | 23 |
| chr1:145592633\|145595220 | hsa_circ_0007755 | 12 | 8 | 3 | 8 | 9 | 4 | 4 | 18 | 6 | 97 | 47 | 29 |
| chr1:14057495\|14068652 | hsa_circ_0005986 | 17 | 5 | 8 | 6 | 14 | 8 | 10 | 18 | 7 | 112 | 46 | 8 |
| chr1:117944808\|117984947 | hsa_circ_0000119 | 133 | 124 | 59 | 58 | 45 | 39 | 39 | 60 | 34 | 704 | 209 | 101 |
| chr1:117944808\|117963271 | hsa_circ_0000118 | 329 | 272 | 126 | 106 | 142 | 100 | 115 | 127 | 60 | 1344 | 422 | 161 |
| chr1:117944808\|117957453 | hsa_circ_0000117 | 200 | 209 | 92 | 78 | 81 | 58 | 120 | 121 | 52 | 1136 | 486 | 166 |
| chr1:117944808\|117948267 | hsa_circ_0000116 | 96 | 69 | 26 | 17 | 32 | 22 | 27 | 36 | 14 | 493 | 118 | 47 |
| chr1:1158624\|1159348 | hsa_circ_0000002 | 40 | 44 | 21 | 53 | 38 | 25 | 35 | 61 | 50 | 232 | 170 | 70 |
| chr1:115005726\|115007010 | hsa_circ_0004127 | 22 | 20 | 7 | 19 | 23 | 11 | 23 | 38 | 27 | 290 | 133 | 69 |
| chr1:115005726\|115006178 | hsa_circ_0013637 | 8 | 14 | 5 | 9 | 14 | 4 | 8 | 10 | 8 | 65 | 38 | 28 |
| chr1:114450631\|114450813 | hsa_circ_0013615 | 18 | 26 | 9 | 13 | 16 | 18 | 18 | 10 | 6 | 81 | 67 | 18 |
| chr1:114391162\|114397671 | hsa_circ_0000111 | 7 | 6 | 3 | 4 | 6 | 6 | 16 | 18 | 14 | 130 | 57 | 12 |
| chr1:114372214\|114377061 | hsa_circ_0000110 | 46 | 29 | 22 | 26 | 29 | 15 | 57 | 37 | 27 | 294 | 176 | 72 |
| chr1:113196220\|113202401 | hsa_circ_0000109 | 20 | 17 | 6 | 8 | 17 | 4 | 12 | 21 | 16 | 141 | 41 | 21 |
| chr1:11133991\|11137005 | hsa_circ_0002448 | 7 | 18 | 4 | 9 | 3 | 2 | 11 | 8 | 4 | 63 | 28 | 7 |
| chr1:10500404\|10502498 | hsa_circ_0006905 | 14 | 11 | 6 | 6 | 6 | 6 | 6 | 10 | 5 | 51 | 35 | 11 |
| chr1:100889778\|100908552 | hsa_circ_0000097 | 25 | 27 | 5 | 15 | 20 | 6 | 16 | 17 | 10 | 138 | 59 | 20 |
